# Experimental and Computational Study of the Antioxidative Potential of Novel Nitro and Amino Substituted Benzimidazole/Benzothiazole-2-Carboxamides with Antiproliferative Activity

**DOI:** 10.3390/antiox8100477

**Published:** 2019-10-12

**Authors:** Maja Cindrić, Irena Sović, Marija Mioč, Lucija Hok, Ida Boček, Petra Roškarić, Kristina Butković, Irena Martin-Kleiner, Kristina Starčević, Robert Vianello, Marijeta Kralj, Marijana Hranjec

**Affiliations:** 1 Department of Organic Chemistry, Faculty of Chemical Engineering and Technology, University of Zagreb, Marulićev trg 19, HR-10000 Zagreb, Croatia; maleksic@fkit.hr (M.C.); isovic@fkit.hr (I.S.); ibocek@fkit.hr (I.B.); petra.roskaric@fkit.hr (P.R.); kristina.butkovic@fkit.hr (K.B.); 2 Division of Molecular Medicine, Ruđer Bošković Institute, Bijenička cesta 54, HR-10000 Zagreb, Croatia; marija.cosic@irb.hr (M.M.); irena.martin.kleiner@irb.hr (I.M.-K.); 3 Division of Organic Chemistry and Biochemistry, Ruđer Bošković Institute, Bijenička cesta 54, HR-10000 Zagreb, Croatia; lucija.hok@irb.hr; 4 Department of Chemistry and Biochemistry, Faculty of Veterinary Medicine, University of Zagreb, Heinzelova 55, HR-10000 Zagreb, Croatia; kristina.starcevic@vef.hr

**Keywords:** benzimidazoles, benzothiazoles, carboxamides, antiproliferative activity, antioxidative activity, ROS, DFT calculations

## Abstract

We present the synthesis of a range of benzimidazole/benzothiazole-2-carboxamides with a variable number of methoxy and hydroxy groups, substituted with nitro, amino, or amino protonated moieties, which were evaluated for their antiproliferative activity in vitro and the antioxidant capacity. Antiproliferative features were tested on three human cancer cells, while the antioxidative activity was measured using 1,1-diphenyl-picrylhydrazyl (DPPH) free radical scavenging and ferric reducing antioxidant power (FRAP) assays. Trimethoxy substituted benzimidazole-2-carboxamide **8** showed the most promising antiproliferative activity (IC_50_ = 0.6–2.0 µM), while trihydroxy substituted benzothiazole-2-carboxamide **29** was identified as the most promising antioxidant, being significantly more potent than the reference butylated hydroxytoluene BHT in both assays. Moreover, the latter also displays antioxidative activity in tumor cells. The measured antioxidative capacities were rationalized through density functional theory (DFT) calculations, showing that **29** owes its activity to the formation of two [O•∙∙∙H–O] hydrogen bonds in the formed radical. Systems **8** and **29** were both chosen as lead compounds for further optimization of the benzazole-2-carboxamide scaffold in order to develop more efficient antioxidants and/or systems with the antiproliferative activity.

## 1. Introduction

Being one of the well-known privileged building substructures in medicinal chemistry, benzimidazoles and benzothiazoles have important roles as the constituents of various biologically important systems [1,2]. Among versatile pharmacological features, the most important ones for the rational design of novel bioactive compounds are antitumor, antimicrobial, antiviral, anti-inflammatory activities [3,4,5,6,7,8]. We have described the antitumor potential of various benzimidazole and/or benzothiazole derivatives bearing amidino, carboxamido, amino, halogen, cyano, amino, or nitro substituents placed at different positions on the mentioned scaffolds. The results showed that amidino substituents, located at the termini of the molecule, have a significant influence for the interaction with biological targets causing enhanced antitumor activity [9,10]. 

It has been widely demonstrated that oxidative stress participates in all stages of chemical carcinogenesis [11,12]. The former refers to an increased production of reactive oxygen species (ROS), which could stimulate damage of many essential macromolecules such as DNA, proteins, and lipids [13,14]. Indeed, it has been proven that there is an implication of ROS in the cell signaling pathway of many chronic diseases, like diabetes mellitus, atherosclerosis, myocardial infarction, arthritis, inflammation, or neurodegenerative diseases [15,16]. At high levels, ROS can easily react with DNA causing genomic instability, with membrane lipids leading to alterations of the membrane permeability, while reaction with the proteins could result in the oxidative modification of their structure [17,18]. In the last few decades, the synthesis and application of new antioxidants are gaining importance due to the development of more promising and effective compounds relative to the standard antioxidants such as β-carotene, vitamin A, vitamin C etc. 

Recently, several studies described the antioxidative potential of various benzimidazoles and benzothiazoles [19,20], including our work on the methoxy amidino substituted benzimidazoles with antiproliferative activity [21]. Our focus was to inspect the effect of the number of methoxy groups and the type of amidine substituent on the antioxidative and antiproliferative features, with an important conclusion that systems with the highest antioxidant activities are less cytotoxic and do not inactivate the cancer cell line proliferation. We also showed the antioxidative potency of 2-aryl substituted benzimidazoles and benzothiazoles having a flexible number of hydroxy groups and different types of amidino substituents, which strongly affected their antioxidative activity and reducing power [22]. Salicylanilide and benzamide derivatives, besides a broad range of multipurpose biological activities, have also been studied as systems with good antioxidative potential [23]. We recently investigated the antioxidative potency of a small library of *N*-arylbenzamides substituted with a differing number of methoxy and hydroxy groups, having either amino or amino protonated moieties, significantly differing in their antioxidative activity [24]. Computational analysis was used to explain the observed trends and confirmed that monocationic molecules bearing amino protonated groups were better antioxidants than their free amino substituted analogues.

In this work, we further broadened our investigations by evaluating the influence of other heteroaromatic benzazole nuclei on antioxidative potentials. Thus, we designed and prepared novel benzamide derivatives, having either benzimidazole or benzothiazole scaffolds, substituted with either nitro, amino, or amino protonated moieties, having a variable number of methoxy and hydroxy groups. We inspected their antioxidative potential, as well as the antiproliferative activity. Specifically, we analyzed the influence of the type and the number of the substituents on the benzazole scaffold in order to choose lead compound(s), which will be further optimized as promising antioxidants and/or potential antitumor agents. The obtained results will be discussed in the context of our previous results for the biological activity of analogous 2-aryl substituted benzazoles, as well as salicylanilide and benzamide derivatives bearing the same substituents.

## 2. Materials and Methods 

### 2.1. Chemistry

#### 2.1.1. General Methods

All chemicals were purchased from commercial suppliers such as Aldrich, Fluka, and Acros. Melting points were determined on the Original Keller Mikroheitztisch apparatus (Reichert, Wien), SMP11 Bibby and Büchi 535 apparatus. ^1^H and APT ^13^C NMR spectra were attained on Varian Gemini 300 or Varian Gemini 600 spectrophotometers at either 300, 600, 150, or 75 MHz, respectively. All NMR spectra were recorded in DMSO-*d_6_* solutions with TMS as an internal standard. Chemical shifts are reported in ppm (δ) relative to TMS. All compounds were routinely checked by thin layer chromatography (TLC) using precoated Merck silica gel 60F-254 plates and the spots were detected under UV light (254 nm). Column chromatography (CC) was performed using silica gel (0.063–0.2 mm) Fluka; glass column was slurry-packed under gravity.

#### 2.1.2. General Method for the Preparation of Methoxy Substituted Nitro Benzamides **6**–**11**

To a solution of methoxy substituted benzoyl chlorides **1–3** in dry toluene, a solution of 2-amino-5(6)-nitrobenzimidazole **4** and 2-amino-6-nitrobenzothiazole **5** in dry toluene was added drop wise, followed by the addition of Et_3_N, and refluxed for 20 h. After cooling, the resulting products were filtered off and recrystallized from ethanol/DMF to have the matching benzamides. 

##### 2-methoxy-N-[5(6)-nitrobenzimidazol-2-yl]benzamide **6**

**6** was prepared using the above described method from 2-methoxybenzoyl chloride **1** (1.45 g, 8.52 mmol) and 2-amino-5(6)-nitrobenzimidazole **4** (1.52 g, 8.52 mmol) in dry toluene (30 mL) followed by the addition of Et_3_N (1.70 mL, 11.92 mmol) to obtain 1.75 g (66%) of light yellow powder; mp 238–240 °C; ^1^H NMR (300 MHz, DMSO) (δ/ppm): 12.89 (s, 1H, NH_benzim._), 11.65 (s, 1H, NH_amide_), 8.37 (s, 1H, H_arom._), 8.08 (dd, *J_1_* = 8.8 Hz, *J_2_* = 2.2 Hz, 1H, H_arom._), 7.79 (dd, *J_1_* = 7.6 Hz, *J_2_* = 1.5 Hz, 1H, H_arom._), 7.70–7.56 (m, 2H, H_arom._), 7.25 (d, *J* = 8.4 Hz, 1H, H_arom._), 7.13 (t, *J* = 7.5 Hz, 1H, H_arom._), 3.96 (s, 3H, OCH_3_); APT ^13^C NMR (75 MHz, DMSO) (δ/ppm): 165.8, 157.6, 134.1, 130.7, 122.5, 121.2, 112.8, 56.7; Anal. Calcd. for C_15_H_12_N_4_O_4_: C, 57.69; H, 3.87; N, 17.94. Found: C, 57.85; H, 3.55; N, 18.20%.

##### 2,4-dimethoxy-N-[5(6)-nitrobenzimidazol-2-yl]benzamide **7**

**7** was prepared using the above described method from 2,4-dimethoxybenzoyl chloride **2** (1.26 g, 6.26 mmol) and 2-amino-5(6)-nitrobenzimidazole **4** (1.12 g, 6.26 mmol) in dry toluene (30 mL) followed by the addition of Et_3_N (1.22 mL, 8.76 mmol) to obtain 1.50 g (70%) of light yellow powder; mp > 250 °C; ^1^H NMR (300 MHz, DMSO) (δ/ppm): 12.85 (s, 1H, NH_benzim._), 11.21 (s, 1H, NH_amide_), 8.39–8.31 (m, 1H, H_arom._), 8.07 (dd, *J_1_* = 8.8 Hz, *J_2_* = 2.3 Hz, 1H, H_arom._), 7.90 (d, *J* = 8.6 Hz, 1H, H_arom._), 7.69–7.58 (m, 1H, H_arom._), 6.78–6.73 (m, 2H, H_arom._), 4.02 (s, 3H, OCH_3_), 3.88 (s, 3H, OCH_3_); APT ^13^C NMR (150 MHz, DMSO) (δ/ppm): 164.3, 159.2, 132.6, 117.8, 116.6, 112.6, 111.8, 106.5, 98.7, 56.5, 55.7; Anal. Calcd. for C_16_H_14_N_4_O_5_: C, 56.14; H, 4.12; N, 16.37. Found: C, 56.36; H, 4.32; N, 16.15%.

##### 3,4,5-trimethoxy-N-[5(6)-nitrobenzimidazol-2-yl]benzamide **8**

**8** was prepared using the above described method from 3,4,5-trimethoxybenzoyl chloride **3** (1.88 g, 8.15 mmol) and 2-amino-5(6)-nitrobenzimidazole **4** (1.45 g, 8.15 mmol) in dry toluene (30 mL) followed by the addition of Et_3_N (1.60 mL, 11.41 mmol) to obtain 0.85 g (28%) of light yellow powder; mp 244–248 °C; ^1^H NMR (300 MHz, DMSO) (δ/ppm): 12.89 (s, 1H, NH_benzim._), 12.34 (s, 1H, NH_amide_), 8.38 (s, 1H, H_arom._), 8.09 (dd, *J_1_* = 8.8 Hz, *J_2_* = 2.3 Hz, 1H, H_arom._), 7.65 (d, *J* = 8.9 Hz, 1H, H_arom._), 7.52 (s, 2H, H_arom._), 3.90 (s, 6H, OCH_3_), 3.76 (s, 3H, OCH_3_); APT ^13^C NMR (100 MHz, DMSO) (δ/ppm): 152.7 (2C), 141.3, 127.1, 105.9 (2C), 60.1, 56.1 (2C); Anal. Calcd. for C_17_H_16_N_4_O_6_: C, 54.84; H, 4.33; N, 15.05. Found: C, 54.66; H, 4.21; N, 15.25%.

##### 2-methoxy-N-(6-nitrobenzothiazol-2-yl)benzamide **9**

**9** was prepared using the above described method from 2-methoxybenzoyl chloride **1** (1.85 g, 10.98 mmol) and 2-amino-6-nitrobenzothiazole **5** (2.15 g, 10.98 mmol) in dry toluene (30 mL) followed by the addition of Et_3_N (2.10 mL, 14.99 mmol) to obtain 2.25 g (62%) of light grey powder; mp 248–250 °C; ^1^H NMR (300 MHz, DMSO) (δ/ppm): 12.47 (s, 1H, NH_amide_), 9.08 (d, *J* = 2.3 Hz, 1H, H_arom._), 8.29 (dd, *J_1_* = 8.9 Hz, *J_2_* = 2.4 Hz, 1H, H_arom._), 7.91 (d, *J* = 8.9 Hz, 1H, H_arom._), 7.73 (dd, *J_1_* = 7.6 Hz, *J_2_* = 1.6 Hz, 1H, H_arom._), 7.60 (t, *J* = 7.1 Hz, 1H, H_arom._), 7.23 (d, *J* = 8.4 Hz, 1H, H_arom._), 7.11 (t, *J* = 7.4 Hz, 1H, H_arom._), 3.93 (s, 3H, OCH_3_); APT ^13^C NMR (151 MHz, DMSO) (δ/ppm): 165.6, 163.2, 157.3, 153.4, 143.1, 133.9, 132.3, 130.3, 121.8, 121.3, 120.7, 120.6, 119.1, 112.3, 56.1; Anal. Calcd. for C_15_H_11_N_3_O_4_S: C, 54.71; H, 3.37; N, 12.76. Found: C, 54.86; H, 3.52; N, 12.92%.

##### 2,4-dimethoxy-N-(6-nitrobenzothiazol-2-yl)benzamide **10**

**10** was prepared using the above described method from 2,4-dimethoxybenzoyl chloride **2** (1.40 g, 6.96 mmol) and 2-amino-6-nitrobenzothiazole **5** (1.36 g, 6.96 mmol) in dry toluene (30 mL) followed by the addition of Et_3_N (1.36 mL, 9.73 mmol) to obtain 1.55 g (62%) of light grey powder; mp > 250 °C; ^1^H NMR (300 MHz, DMSO) (δ/ppm): 11.98 (s, 1H, NH_amide_), 9.09 (d, *J* = 2.4 Hz, 1H, H_arom._), 8.30 (dd, *J_1_* = 8.9 Hz, *J_2_* = 2.4 Hz, 1H, H_arom._), 7.92 (d, *J* = 8.9 Hz, 1H, H_arom._), 7.85 (d, *J* = 8.6 Hz, 1H, H_arom._), 6.77 (d, *J* = 2.1 Hz, 1H, H_arom._), 6.74 (dd, *J_1_* = 8.7 Hz, *J_2_* = 2.2 Hz, 1H, H_arom._), 4.01 (s, 3H, OCH_3_), 3.88 (s, 3H, OCH_3_); APT ^13^C NMR (75 MHz, DMSO) (δ/ppm): 165.1, 164.8, 163.8, 160.0, 153.9, 143.5, 133.2, 132.9, 122.3, 121.0, 119.5, 113.0, 107.0, 99.2, 57.0, 56.3; Anal. Calcd. for C_16_H_13_N_3_O_5_S: C, 53.48; H, 3.65; N, 11.69. Found: C, 53.66; H, 3.50; N, 11.90%.

##### 3,4,5-trimethoxy-N-(6-nitrobenzothiazol-2-yl)benzamide **11**

**11** was prepared using the above described method from 3,4,5-trimethoxybenzoyl chloride **3** (1.51 g, 6.55 mmol) and 2-amino-6-nitrobenzothiazole **5** (1.28 g, 6.55 mmol) in dry toluene (30 mL) followed by the addition of Et_3_N (1.30 mL, 9.16 mmol) to obtain 2.46 g (96%) of grey powder; mp > 250 °C; ^1^H NMR (300 MHz, DMSO) (δ/ppm): 13.24 (s, 1H, NH_amide_), 9.10 (d, *J* = 2.4 Hz, 1H, H_arom._), 8.31 (dd, *J_1_* = 8.9 Hz, *J_2_* = 2.4 Hz, 1H, H_arom._), 7.92 (d, *J* = 8.9 Hz, 1H, H_arom._), 7.55 (s, 2H, H_arom._), 3.90 (s, 6H, OCH_3_), 3.77 (s, 3H, OCH_3_); APT ^13^C NMR (100 MHz, DMSO) (δ/ppm): 164.5, 152.8 (2C), 143.0, 141.6, 132.2, 126.0, 121.8, 120.4, 119.1, 106.0 (2C), 60.1, 56.1 (2C); Anal. Calcd. for C_17_H_15_N_3_O_6_S: C, 52.44; H, 3.88; N, 10.79. Found: C, 52.68; H, 3.62; N, 10.93%.

#### 2.1.3. General Method for the Preparation of Methoxy Substituted Amino Benzamides **12**–**17**

Methoxy substituted nitro benzamides **6**–**11**, and a solution of SnCl_2_ × 2H_2_O in MeOH and concentrated HCl (1:2) were refluxed for 0.5 h. After cooling, the mixture was concentrated at a reduced pressure and dissolved in water. Such a solution was treated with 20% NaOH to pH 14. The product was filtered off and washed with water to obtain methoxy substituted amino benzamides.

##### *N*-[5(6)-aminobenzimidazol-2-yl]-2-methoxybenzamide **12**

**12** was prepared using the above described method from **6** (1.03 g, 3.31 mmol), SnCl_2_ × 2H_2_O (5.22 g, 23.13 mmol), HCl_concd_ (6.0 mL) and MeOH (12.0 mL) to obtain 0.11 g (12%) of brown powder; mp 218–220 °C; ^1^H NMR (300 MHz, DMSO) (δ/ppm): 11.39 (bs, 2H, NH_benzim._, NH_amide_), 7.76 (dd, *J_1_* = 7.6 Hz, *J_2_* = 1.5 Hz, 1H, H_arom._), 7.52 (t, *J* = 7.0 Hz, 1H, H_arom._), 7.18 (d, *J* = 8.3 Hz, 1H, H_arom._), 7,11–7,03 (m, 2H, H_arom._), 6.64 (s, 1H, H_arom._), 6.40 (dd, *J_1_* = 8.4 Hz, *J_2_* = 2.0 Hz, 1H, H_arom._), 4.67 (s, 2H, NH_2_), 3.92 (s, 3H, OCH_3_); APT ^13^C NMR (125 MHz, DMSO) (δ/ppm): 157.0, 143.8, 133.1, 130.2, 120.7, 112.3, 110.4, 56.1; Anal. Calcd. for C_15_H_14_N_4_O_2_: C, 63.82; H, 5.00; N, 19.85. Found: C, 63.99; H, 5.28; N, 19.60%.

##### *N*-[5(6)-aminobenzimidazol-2-yl]-2,4-dimethoxybenzamide **13**

**13** was prepared using the above described method from **7** (1.00 g, 2.92 mmol), SnCl_2_ × 2H_2_O (4.61 g, 20.44 mmol), HCl_concd_ (5.0 mL) and MeOH (10.0 mL) to obtain 0.46 g (51%) of yellow powder; mp 179–183 °C; ^1^H NMR (300 MHz, DMSO) (δ/ppm): 11.76 (s, 1H, NH_benzim._), 10.63 (s, 1H, NH_amide_), 7.93 (d, *J* = 8.5 Hz, 1H, H_arom._), 7.11 (d, *J* = 8.1 Hz, 1H, H_arom._), 6.79–6.70 (m, 2H, H_arom._), 6.67 (s, 1H, H_arom._), 6.44 (dd, *J_1_* = 8.4, *J_2_* = 2.0 Hz, 1H, H_arom._), 4.72 (s, 2H, NH_2_), 4.03 (s, 3H, OCH_3_), 3.87 (s, 3H, OCH_3_); APT ^13^C NMR (75 MHz, DMSO) (δ/ppm): 164.5, 159.5, 144.2, 133.0, 110.9, 107.0, 99.2, 57.0, 56.2; Anal. Calcd. for C_16_H_16_N_4_O_3_: C, 61.53; H, 5.16; N, 17.94. Found: C, 61.69; H, 5.30; N, 17.70%.

##### *N*-[5(6)-aminobenzimidazol-2-yl]-3,4,5-trimethoxybenzamide **14**

**14** was prepared using the above described method from **8** (1.00 g, 2.68 mmol), SnCl_2_ × 2H_2_O (5.00 g, 22.29 mmol), HCl_concd_ (9.5 mL) and MeOH (9.5 mL) to obtain 0.83 g (90%) of light green powder; mp 198–201 °C; ^1^H NMR (300 MHz, DMSO) (δ/ppm): 11.93 (s, 2H, NH_benzim.,_ NH_amide_), 7.48 (s, 2H, H_arom._), 7.10 (d, *J* = 8.40 Hz, 1H, H_arom._), 6.64 (s, 1H, H_arom._), 6.46 (d, *J* = 8.40 Hz, 1H, H_arom._), 4.85 (s, 2H, NH_2_), 3.86 (s, 6H, OCH_3_), 3.73 (s, 3H, OCH_3_); APT ^13^C NMR (75 MHz, DMSO) (δ/ppm): 152.9 (2C), 144.9 (2C), 140.8, 131.3, 110.7, 106.1 (2C), 97.3, 60.6, 56.4 (2C); Anal. Calcd. for C_17_H_18_N_4_O_4_: C, 59.64; H, 5.30; N, 16.37. Found: C, 59.82; H, 5.55; N, 16.50%.

##### *N*-(6-aminobenzothiazol-2-yl)-2-methoxybenzamide **15**

**15** was prepared using the above described method from **9** (1.50 g, 4.55 mmol), SnCl_2_ × 2H_2_O (7.20 g, 31.91 mmol), HCl_concd_ (8.0 mL) and MeOH (16.0 mL) to obtain 0.62 g (50%) of yellow powder; mp 210–214 °C; ^1^H NMR (300 MHz, DMSO) (δ/ppm): 11.69 (s, 1H, NH_amide_), 7.76 (dd, *J_1_* = 7.6 Hz, *J_2_* = 1.7 Hz, 1H, H_arom._), 7.58 (t, *J* = 7.0 Hz, 1H, H_arom._), 7.44 (d, *J* = 8.6 Hz, 1H, H_arom._), 7.23 (d, *J* = 8.3 Hz, 1H, H_arom._), 7.11 (t, *J* = 7.5 Hz, 1H, H_arom._), 7.04 (d, *J* = 2.1 Hz, 1H, H_arom._), 6.73 (dd, *J_1_* = 8.6 Hz, *J_2_* = 2.2 Hz, 1H, H_arom._), 5.20 (s, 2H, NH_2_), 3.95 (s, 3H, OCH_3_); APT ^13^C NMR (125 MHz, DMSO) (δ/ppm): 164.0, 157.1, 152.6, 145.9, 139.6, 133.5, 133.2, 130.3, 121.6, 120.9, 120.7, 114.6, 112.3, 104.0, 56.1; Anal. Calcd. for C_15_H_13_N_3_O_2_S: C, 60.19; H, 4.38; N, 14.04. Found: C, 60.44; H, 4.57; N, 14.23%.

##### *N*-(6-aminobenzothiazol-2-yl)-2,4-dimethoxybenzamide **16**

**16** was prepared using the above described method from **10** (1.00 g, 2.78 mmol), SnCl_2_ × 2H_2_O (4.39 g, 19.45 mmol), HCl_concd_ (5.0 mL) and MeOH (10.0 mL) to obtain 0.79 g (98%) of light brown powder; mp 208–210 °C; ^1^H NMR (300 MHz, DMSO) (δ/ppm): 11.26 (s, 1H, NH_amide_), 7.85 (d, *J* = 8.6 Hz, 1H, H_arom._), 7.41 (d, *J* = 8.6 Hz, 1H, H_arom._), 7.02 (d, *J* = 2.1 Hz, 1H, H_arom._), 6.76 – 6.67 (m, 3H, H_arom._), 5.16 (s, 2H, NH_2_), 4.00 (s, 3H, OCH_3_), 3.86 (s, 3H, OCH_3_); APT ^13^C NMR (150 MHz, DMSO) (δ/ppm): 163.8, 159.1, 145.6, 139.8, 133.3, 132.5, 120.6, 114.4, 106.3, 104.1, 98.7, 56.4, 55.7; Anal. Calcd. for C_16_H_15_N_3_O_3_S: C, 58.35; H, 4.59; N, 12.76. Found: C, 58.55; H, 4.42; N, 12.96%.

##### *N*-(6-aminobenzothiazol-2-yl)-3,4,5-trimethoxybenzamide **17**

**17** was prepared using the above described method from **11** (1.50 g, 3.85 mmol), SnCl_2_ × 2H_2_O (6.09 g, 26.99 mmol), HCl_concd_ (5.0 mL) and MeOH (10.0 mL) to obtain 0.73 g (61%) of yellow powder; mp > 250 °C; ^1^H NMR (300 MHz, DMSO) (δ/ppm): 12.53 (s, 1H, NH_amide_), 7.50 (s, 2H, H_arom._), 7.45 (d, *J* = 8.6 Hz, 1H, H_arom._), 7.05 (d, *J* = 2.1 Hz, 1H, H_arom._), 6.75 (dd, *J_1_* = 8.6 Hz, *J_2_* = 2.2 Hz, 1H, H_arom._), 5.21 (s, 2H, NH_2_), 3.89 (s, 6H, OCH_3_), 3.75 (s, 3H, OCH_3_); APT ^13^C NMR (75 MHz, DMSO) (δ/ppm): 153.2, 146.4, 133.5, 127.4, 121.2, 115.0, 106.1 (2C), 104.4, 60.6, 56.6 (2C); Anal. Calcd. for C_17_H_17_N_3_O_4_S: C, 56.81; H, 4.77; N, 11.69. Found: C, 56.64; H, 4.52; N, 11.52%.

#### 2.1.4. General Method for the Preparation of Amino Substituted Benzamides as Hydrochloride Salts **18**–**23** and **32**

A suspension of the corresponding amino substituted benzamides **12–17** and **31** in absolute ethanol saturated with HCl_(g)_ was stirred at room temperature for 24 h. Diethyl ether was added to the reaction mixture and the resulting precipitate was filtered off and washed with diethyl ether to obtain hydrochlorides salts.

##### *N*-[5(6)-aminobenzimidazol-2-yl]-2-methoxybenzamide hydrochloride **18**

**18** was prepared using the above described method from **12** (0.03 g, 0.08 mmol) in absolute ethanol saturated with HCl_(g)_ (5.0 mL) to obtain 0.02 g (85%) of light grey powder; mp 148–152 °C; ^1^H NMR (300 MHz, DMSO) (δ/ppm): 12.03 (s, 2H, NH_benzim._, NH_amide_), 9.82 (bs, 3H, NH_3_^+^), 7.92 (dd, *J_1_* = 7.7 Hz, *J_2_* = 1.7 Hz, 1H, H_arom._), 7.75–7.62 (m, 3H, H_arom._), 7.31 (d, *J* = 8.2 Hz, 2H, H_arom._), 7.18 (t, *J* = 7.6 Hz, 1H, H_arom._), 4.05 (s, 3H, OCH_3_); APT ^13^C NMR (75 MHz, DMSO) (δ/ppm): 165.0, 157.9, 145.8, 135.2, 133.2, 131.2, 131.1, 129.7, 121.5, 120.8, 118.0, 115.1, 113.0, 107.7, 56.8; Anal. Calcd. for C_15_H_15_N_4_O_2_Cl: C, 56.52; H, 4.74; N, 17.58. Found: C, 56.74; H, 4.52; N, 17.82%.

##### *N*-[5(6)-aminobenzimidazol-2-yl]-2,4-dimethoxybenzamide hydrochloride **19**

**19** was prepared using the above described method from **13** (0.04 g, 0.15 mmol) in absolute ethanol saturated with HCl_(g)_ (5.0 mL) to obtain 0.02 g (48%) of light rose powder; mp > 250 °C; ^1^H NMR (300 MHz, DMSO) (δ/ppm): 9.62 (bs, 3H, NH_3_^+^), 7.75 (d, *J* = 8.4 Hz, 1H, H_arom._), 7.73 (d, *J* = 1.9 Hz, 1H, H_arom._), 7.61 (s, 1H, H_arom._), 7.54 (d, *J* = 7.9 Hz, 1H, H_arom._),7.31 (dd, *J_1_* = 8.6 Hz, *J_2_* = 1.8 Hz, 1H, H_arom._), 7.02 (d, *J* = 7.9 Hz, 1H, H_arom._), 3.92 (s, 3H, OCH_3_), 3.78 (s, 3H, OCH_3_); APT ^13^C NMR (150 MHz, DMSO) (δ/ppm): 165.8, 153.3, 146.8, 142.3, 132.2, 130.9, 128.6, 127.0, 122.1, 118.8, 115.2, 108.9, 106.8, 60.7, 58.7; Anal. Calcd. for C_16_H_17_N_4_O_3_Cl: C, 55.10; H, 4.91; N, 16.06. Found: C, 55.34; H, 4.74; N, 16.24%.

##### *N*-(6-aminobenzimidazol-2-yl)-3,4,5-trimethoxybenzamide hydrochloride **20**

**20** was prepared using the above described method from **14** (0.08 g, 0.23 mmol) in absolute ethanol saturated with HCl_(g)_ (5.0 mL) to obtain 0.08 g (91%) of beige powder; mp > 250 °C; ^1^H NMR (300 MHz, DMSO) (δ/ppm): 9.51 (bs, 4H, NH_amide_, NH_3_^+^), 7.79 (s, 1H, H_arom._), 7.77 (d, *J* = 8.8 Hz, 2H, H_arom._), 7.63 (s, 2H, H_arom._), 7.37 (dd, *J_1_* = 8.6 Hz, *J_2_* = 1.9 Hz, 1H, H_arom._), 3.93 (s, 6H, OCH_3_), 3.78 (s, 3H, OCH_3_); APT ^13^C NMR (75 MHz, DMSO) (δ/ppm): 165.6, 153.6 (2C), 146.3, 142.2, 131.4, 130.0, 128.9, 126.7, 119.3, 115.3, 109.1, 106.8 (2C), 60.7, 56.8 (2C); Anal. Calcd. for C_17_H_19_N_4_O_4_Cl: C, 53.90; H, 5.06; N, 14.79. Found: C, 53.76; H, 5.28; N, 14.95%.

##### *N*-(6-aminobenzothiazol-2-yl)-2-methoxybenzamide hydrochloride **21**

**21** was prepared using the above described method from **15** (0.05 g, 0.15 mmol) in absolute ethanol saturated with HCl_(g)_ (5.0 mL) to obtain 0.05 g (93%) of beige powder; mp > 250 °C; ^1^H NMR (300 MHz, DMSO) (δ/ppm): 12.16 (s, 1H, NH_amide_), 10.53 (bs, 3H, NH_3_^+^), 8.09 (s, 1H, H_arom._), 7.87 (d, *J* = 8.6 Hz, 1H, H_arom._), 7.77 (d, *J* = 6.8 Hz, 1H, H_arom._), 7.61 (t, *J* = 7.5 Hz, 1H, H_arom._), 7.50 (d, *J* = 8.4 Hz, 1H, H_arom._), 7.25 (d, *J* = 8.4 Hz, 1H, H_arom._), 7.13 (t, *J* = 7.4 Hz, 1H, H_arom._), 3.96 (s, 3H, OCH_3_); APT ^13^C NMR (75 MHz, DMSO) (δ/ppm): 165.6, 159.4, 157.7, 148.4, 134.6, 133.0, 130.9, 127.8, 122.0 (2C), 121.4, 121.3, 116.9, 112.8, 56.6; Anal. Calcd. for C_15_H_14_N_3_O_2_SCl: C, 53.65; H, 4.20; N, 12.51. Found: C, 53.48; H, 4.04; N, 12.76%.

##### *N*-(6-aminobenzothiazol-2-yl)-2,4-dimethoxybenzamide hydrochloride **22**

**22** was prepared using the above described method from **16** (0.05 g, 0.14 mmol) in absolute ethanol saturated with HCl_(g)_ (5.0 mL) to obtain 0.05 g (94%) of beige powder; mp > 250 °C; ^1^H NMR (300 MHz, DMSO) (δ/ppm): 11.7 (s, 1H, NH_amide_), 10.5 (bs, 3H, NH_3_^+^), 8.08 (d, *J* = 1.9 Hz, 1H, H_arom._), 7.87 (dd, *J_1_* = 8.6 Hz, *J_2_* = 3.5 Hz, 2H, H_arom._), 7.48 (dd, *J_1_* = 8.6 Hz, *J_2_* = 2.0 Hz, 1H, H_arom._), 6.80–6.67 (m, 2H, H_arom._), 4.02 (s, 3H, OCH_3_), 3.88 (s, 3H, OCH_3_); APT ^13^C NMR (150 MHz, DMSO) (δ/ppm): 164.4, 163.8, 159.3, 159.0, 148.0, 132.7, 132.6, 127.2, 121.6, 121.2, 116.7, 112.6, 106.5, 98.7, 56.5, 55.8; Anal. Calcd. for C_16_H_16_N_3_O_3_SCl: C, 52.53; H, 4.41; N, 11.49. Found: C, 52.69; H, 4.65; N, 11.72%.

##### *N*-(6-aminobenzothiazol-2-yl)-3,4,5-trimethoxybenzamide hydrochloride **23**

**23** was prepared using the above described method from **17** (0.05 g, 0.15 mmol) in absolute ethanol saturated with HCl_(g)_ (5.0 mL) to obtain 0.05 g (87%) of light brown powder; mp 248–250 °C; ^1^H NMR (300 MHz, DMSO) (δ/ppm): 13.04 (s, 1H, NH_amide_), 10.66 (bs, 3H, NH_3_^+^), 8.10 (s, 1H, H_arom._), 7.87 (d, *J* = 8.3 Hz, 1H, H_arom._), 7.54–7.49 (m, 3H, H_arom._), 3.89 (s, 6H, OCH_3_), 3.76 (s, 3H, OCH_3_); APT ^13^C NMR (100 MHz, DMSO) (δ/ppm): 165.2, 163.7, 160.2, 152.8, 141.4, 132.4, 127.3, 126.3, 121.7, 121.1, 116.7, 105.9, 60.1, 56.1; Anal. Calcd. for C_17_H_18_N_3_O_4_SCl: C, 51.58; H, 4.58; N, 10.62. Found: C, 51.40; H, 4.76; N, 10.79%.

##### *N*-[5(6)-aminobenzimidazol-2-yl]-2-hydroxy-4-methoxybenzamide **32**

**32** was prepared using the above described method from **31** (0.08 g, 0.27 mmol) in absolute ethanol saturated with HCl_(g)_ (5.0 mL) to obtain 0.05 g (59%) of light green powder; mp 250 °C; ^1^H NMR (300 MHz, DMSO) (δ/ppm): 10.64 (bs, 4H, OH, NH_3_^+^), 7.98 (d, *J* = 9.1 Hz, 1H, H_arom._), 7.67 (d, *J* = 8.3 Hz, 1H, H_arom._), 7.66 (d, *J* = 2.0 Hz, 1H, H_arom._), 7.34 (dd, *J_1_* = 8.6 Hz, *J_2_* = 1.9 Hz, 1H, H_arom._), 6.11–6.58 (s, 2H, H_arom._), 3.81 (s, 3H, OCH_3_); APT ^13^C NMR (75 MHz, DMSO) (δ/ppm): 165.0, 161.0, 132.7, 130.4, 129.2, 128.4, 119.3, 114.1, 108.2, 107.4, 101.5, 56.0; Anal. Calcd. for C_15_H_15_N_4_O_3_Cl: C, 53.82; H, 4.52; N, 16.74. Found: C, 53.69; H, 4.37; N, 16.86%.

#### 2.1.5. General Method for the Preparation of Hydroxy Substituted Amino Benzamides **24**–**31**

To a stirring solution of the matching methoxy substituted amino benzamides in absolute dichloromethane at −70 °C under argon, boron tribromide was added after 15 min. After stirring at −70 °C for 2 h, the mixture was further stirred at −18 °C for 2 days. Methanol was added to the mixture and the solvent was concentrated at a reduced pressure. The solid was suspended in water and filtered off to obtain hydroxy substituted amino benzamides.

##### 2-hydroxy-N-[5(6)-nitrobenzimidazol-2-yl]benzamide **24**

**24** was prepared using the above described method from **6** (0.40 g, 1.28 mmol) and BBr_3_ (3.84 mL, 3.84 mmol) in absolute CH_2_Cl_2_ (20.0 mL) to obtain 0.37 g (97%) of light yellow powder; mp > 250 °C; ^1^H NMR (300 MHz, DMSO) (δ/ppm): 8.34 (d, *J* = 2.2 Hz, 1H, H_arom._), 8.16 (dd, *J_1_* = 8.8 Hz, *J_2_* = 2.3 Hz, 1H, H_arom._), 8.03 (dd, *J_1_* = 7.8 Hz, *J_2_* = 1.6 Hz, 1H, H_arom._), 7.65 (d, *J* = 8.8 Hz, 1H, H_arom._), 7.47 (t, *J* = 6.8 Hz, 1H, H_arom._), 7.03–6.93 (m, 2H, H_arom._); APT ^13^C NMR (100 MHz, DMSO) (δ/ppm): 158.6, 150.9, 142.8, 134.5, 130.4, 119.2, 118.8, 118.0, 117.2, 113.2, 108.9; Anal. Calcd. for C_14_H_10_N_4_O_4_: C, 56.38; H, 3.38; N, 18.79. Found: C, 56.52; H, 3.62; N, 18.55%.

##### 2-hydroxy-4-methoxy-N-[5(6)-nitrobenzimidazol-2-yl]benzamide **25**

**25** was prepared using the above described method from **7** (0.40 g, 1.17 mmol) and BBr_3_ (7.02 mL, 7.02 mmol) in absolute CH_2_Cl_2_ (20.0 mL) to obtain 0.34 g (93%) of light yellow powder; mp > 250 °C; ^1^H NMR (300 MHz, DMSO) (δ/ppm): 8.34 (d, *J* = 2.1 Hz, 1H, H_arom._), 8.15 (dd, *J_1_* = 8.8 Hz, *J_2_* = 2.2 Hz, 1H, H_arom._), 7.99 (d, *J* = 8.8 Hz, 1H, H_arom._), 7.64 (d, *J* = 8.8 Hz, 1H, H_arom._), 6.58 (dd, *J_1_* = 8.9 Hz, *J_2_* = 2.3 Hz, 1H, H_arom._), 6.52 (d, *J* = 2.2 Hz, 1H, H_arom._), 3.81 (s, 3H, OCH_3_); APT ^13^C NMR (100 MHz, DMSO) (δ/ppm): 164.5, 160.6, 142.8, 132.0, 118.8, 113.2, 110.4, 108.9, 107.0, 101.1, 55.5; Anal. Calcd. for C_15_H_15_N_4_O_5_: C, 54.88; H, 3.68; N, 17.07. Found: C, 54.64; H, 3.80; N, 17.24%.

##### 3,5-dihydroxy-4-methoxy-N-[5(6)-nitrobenzimidazol-2-yl]benzamide **26**

**26** was prepared using the above described method from **8** (0.30 g, 0.80 mmol) and BBr_3_ (7.20 mL, 7.20 mmol) in absolute CH_2_Cl_2_ (20.0 mL) to obtain 0.07 g (28%) of yellow powder; mp 202–205 °C; ^1^H NMR (300 MHz, DMSO) (δ/ppm): 12.21 (bs, 2H, NH_benzim._, NH_amide_), 8.38 (d, *J* = 2.2 Hz, 1H, H_arom._), 8.10 (dd, *J_1_* = 8.8 Hz, *J_2_* = 2.3 Hz, 1H, H_arom._), 7.65 (d, *J* = 8.8 Hz, 1H, H_arom._), 7.43 (d, *J* = 1.9 Hz, 1H, H_arom._), 7.29 (d, *J* = 2.0 Hz, 1H, H_arom._), 3.88 (s, 3H, OCH_3_); APT ^13^C NMR (100 MHz, DMSO) (δ/ppm): 165.8, 150.2, 147.8, 145.7, 142.4, 139.6, 121.4, 118.2, 114.3, 110.1, 109.8, 108.1, 106.3, 104.2, 56.2; Anal. Calcd. for C_15_H_12_N_4_O_6_: C, 52.33; H, 3.51; N, 16.27. Found: C, 52.50; H, 3.76; N, 16.05%.

##### 2-hydroxy-N-(6-nitrobenzothiazol-2-yl)benzamide **27**

**27** was prepared using the above described method from **9** (0.40 g, 1.21 mmol) and BBr_3_ (3.64 mL, 3.64 mmol) in absolute CH_2_Cl_2_ (20.0 mL) to obtain 0.28 g (74%) of white powder; mp > 250 °C; ^1^H NMR (300 MHz, DMSO) (δ/ppm): 12.35 (bs, 1H, OH), 11.78 (bs, 1H, NH_amide_), 9.09 (d, *J* = 2.3 Hz, 1H, H_arom._), 8.30 (dd, *J_1_* = 8.9 Hz, *J_2_* = 2.4 Hz, 1H, H_arom._), 7.99 (dd, *J_1_* = 7.9 Hz, *J_2_* = 1.5 Hz, 1H, H_arom._), 7.91 (d, *J* = 8.9 Hz, 1H, H_arom._), 7.55–7.50 (m, 1H, H_arom._), 7.11–6.99 (m, 2H, H_arom._); APT ^13^C NMR (100 MHz, DMSO) (δ/ppm): 143.1, 135.0, 130.6, 121.9, 119.8, 119.2, 117.2, 116.7; Anal. Calcd. for C_14_H_9_N_3_O_4_S: C, 53.33; H, 2.88; N, 13.33. Found: C, 53.52; H, 2.71; N, 13.56%. 

##### 2-hydroxy-4-methoxy-N-(6-nitrobenzothiazol-2-yl)benzamide **28**

**28** was prepared using the above described method from **10** (0.40 g, 1.11 mmol) and BBr_3_ (6.67 mL, 6.67 mmol) in absolute CH_2_Cl_2_ (20.0 mL) to obtain 0.36 g (99%) of dark yellow powder; mp > 250 °C; ^1^H NMR (300 MHz, DMSO) (δ/ppm): 12.11 (bs, 1H, OH), 9.07 (s, 1H, NH_amide_), 8.30 (dd, *J_1_* = 8.9 Hz, *J_2_* = 2.3 Hz, 1H, H_arom._), 8.01 (d, *J* = 8.9 Hz, 1H, H_arom._), 7.89 (d, *J* = 9.8 Hz, 1H, H_arom._), 6.63 (d, *J* = 8.9 Hz, 1H, H_arom._), 6.58 (s, 1H, H_arom._), 6.46 (d, *J* = 6.8 Hz, 1H, H_arom._), 3.82 (s, 3H, OCH_3_); APT ^13^C NMR (100 MHz, DMSO) (δ/ppm): 164.7, 163.8, 143.0, 142.9, 132.4, 132.1, 121.9, 119.1, 108.8, 107.3, 102.7, 101.3, 55.5; Anal. Calcd. for C_15_H_11_N_3_O_5_S: C, 52.17; H, 3.21; N, 12.17. Found: C, 51.95; H, 3.37; N, 12.02%. 

##### 3,4,5-trihydroxy-N-(6-nitrobenzothiazol-2-yl)benzamide **29**

**29** was prepared using the above described method from **11** (0.40 g, 0.80 mmol) and BBr_3_ (9.30 mL, 9.30 mmol) in absolute CH_2_Cl_2_ (20.0 mL) to obtain 0.28 g (77%) of yellow powder; mp > 250 °C; ^1^H NMR (300 MHz, DMSO) (δ/ppm): 12.89 (s, 1H, NH_amide_), 9.33 (s, 2H, OH), 9.16 (s, 1H, OH), 9.07 (d, *J* = 2.3 Hz, 1H, H_arom._), 8.30 (dd, *J_1_* = 8.9 Hz, *J_2_* = 2.4 Hz, 1H, H_arom._), 7.91 (d, *J* = 8.9 Hz, 1H, H_arom._), 7.17 (s, 2H, H_arom._); APT ^13^C NMR (125 MHz, DMSO) (δ/ppm): 166.1, 164.6, 153.6, 145.7 (2C), 142.9, 138.8, 132.3, 121.7, 121.0, 120.4, 118.9, 108.2 (2C); Anal. Calcd. for C_14_H_9_N_3_O_6_S: C, 48.42; H, 2.61; N, 12.10. Found: C, 48.65; H, 2.43; N, 12.33%.

##### *N*-[5(6)-aminobenzimidazol-2-yl]-2-hydroxybenzamide **30**

**30** was prepared using the above described method from **12** (0.07 g, 0.25 mmol) and BBr_3_ (0.75 mL, 0.75 mmol) in absolute CH_2_Cl_2_ (20.0 mL) to obtain 0.02 g (29%) of grey powder; mp > 250 °C; ^1^H NMR (300 MHz, DMSO) (δ/ppm): 14.20 (bs, 1H, OH), 12.39 (bs, 2H, NH_benzim._, NH_amide_), 7.96 (d, *J* = 6.5 Hz, 1H, H_arom._), 7.32 (t, *J* = 6.8 Hz, 1H, H_arom._), 7.12 (d, *J* = 8.5 Hz, 1H, H_arom._), 6.87–6.76 (m, 2H, H_arom._), 6.63 (d, *J* = 1.8 Hz, 1H, H_arom._), 6.51 (dd, *J_1_* = 8.5 Hz, *J_2_* = 1.9 Hz, 1H, H_arom._), 5.15 (s, 2H, NH_2_); APT ^13^C NMR (100 MHz, DMSO) (δ/ppm): 172.1, 160.5, 150.1, 145.2, 133.0, 130.0, 129.7, 119.8, 117.8, 116.90, 112.2, 111.0, 96.0; Anal. Calcd. for C_14_H_12_N_4_O_2_: C, 62.68; H, 4.51; N, 20.88. Found: C, 62.90; H, 4.38; N, 20.66%.

##### *N*-[5(6)-aminobenzimidazol-2-yl]-2-hydroxy-4-methoxybenzamide **31**

**31** was prepared using the above described method from **13** (0.35 g, 1.12 mmol) and BBr_3_ (6.72 mL, 6.72 mmol) in absolute CH_2_Cl_2_ (20.0 mL) to obtain 0.16 g (47%) of grey powder; mp > 250 °C; ^1^H NMR (600 MHz, DMSO) (δ/ppm): 14.44 (bs, 1H, OH), 12.33 (s, 1H, NH_benzim._), 12.30 (s, 1H, NH_amide_), 7.87 (d, *J* = 8.7 Hz, 1H, H_arom._), 7.11 (d, *J* = 8.5 Hz, 1H, H_arom._), 6.62 (d, *J* = 1.7 Hz, 1H, H_arom._), 6.50 (dd, *J_1_* = 8.5 Hz, *J_2_* = 1.9 Hz, 1H, H_arom._), 6.42 (dd, *J_1_* = 8.7 Hz, *J_2_* = 2.1 Hz, 1H, H_arom._), 6.37 (s, 1H, H_arom._), 5.08 (s, 2H, NH_2_), 3.77 (s, 3H, OCH_3_).; APT ^13^C NMR (100 MHz, DMSO) (δ/ppm): 164.5, 160.4, 132.7, 132.2, 130.0, 128.8, 127.7, 118.7, 113.7, 110.4, 108.7, 107.6, 107.0, 102.6, 101.0, 55.5; Anal. Calcd. for C_15_H_14_N_4_O_3_: C, 60.40; H, 4.73; N, 18.78. Found: C, 60.21; H, 4.56; N, 18.94%.

### 2.2. Biological Activity

#### 2.2.1. Antiproliferative Activity in Vitro

The experiments were performed on four human cell lines, including HCT 116 (colon carcinoma), H 460 (lung carcinoma), MCF-7 (breast carcinoma), and HEK 293 (human embryonic kidney cells), in line with previously published experimental procedures [10,25]. Briefly, the cells were grown in DMEM medium with the addition of 10% fetal bovine serum (FBS), 2 mM l-glutamine, 100 U/mL penicillin, and 100 µg/mL streptomycin, and cultured as monolayers at 37 °C in a humidified atmosphere with 5% CO_2_. Cells were seeded at 2 × 10^3^ cells/well in a standard 96-well microtiter plates and left to attach for 24 h. The next day, a test compound was added in five serial 10-fold dilutions. The rate of cell growth was evaluated after 72 h of incubation with MTT assays. The obtained results are expressed as IC_50_ values, calculated from the concentration-response curve using linear regression analysis by fitting the test concentrations that give PG values above and below the reference value (i.e., 50%). Each test was performed in quadruplicate in at least two individual experiments.

#### 2.2.2. Antioxidative Activity

##### Determination of the Reducing Activity of the Stable Radical 1,1-diphenyl-picrylhydrazyl (DPPH) 

The reducing activity of investigated systems was achieved by the DPPH method according to previously described procedures with modifications to assure the use in a 96-well microplate. Briefly, equal volumes of various concentrations of tested molecules (dissolved in DMSO) were added to the solution of DPPH (final concentration 50 µM in absolute ethanol). Ethanol and DMSO were used as control solutions in line with earlier reports [21,24,26]. 

##### Determination of Ferric Reducing/Antioxidant Power (FRAP assay)

The FRAP method was carried out according to previously described procedures with some modifications to be compatible with an assay on a 96-well microplate. All tests were done in triplicate, while the obtained results were averaged and reported as Fe^2+^ equivalents (Fe^2+^ µmol).

#### 2.2.3. Antioxidative Activity Assay in Cells

For the antioxidative activity assay, 2.5 × 10^4^ cells were seeded into 96-well microtiter plates and left to attach for 24 h. The next day, cells were washed with PBS and incubated in FBS-free DMEM medium with 25 µM DCFH-DA fluorescence dye [27]. After 45 min of incubation, medium was discarded, and cells were washed with PBS. After the washing step, cells were incubated with 100 µM *tert*-Butyl hydroperoxide (TBHP) alone or in combination with antioxidative agents (50 mM *N*-Acetyl-l-cysteine—NAC, or 10 µM tested compounds) in PBS, for 1 h at 37 °C. DCFH-DA fluorescence was recorded on a microplate fluorimeter reader (Tecan) with excitation beam of 485 nm, while the emitted fluorescence was collected at 535 nm. All tests were presented as medians of three independent measurements, done in triplicates. One-way ANOVA with Tukey’s post-hoc test was used for statistical analysis, * *p* < 0.05, ** *p* < 0.01, *** *p* < 0.001.

### 2.3. Computational Details

All geometrical parameters were optimized employing the density functional theory (DFT) B3LYP functional (unrestricted UB3LYP for the radicals), together with the 6–31+G(d) basis set, followed by the vibrational analysis using the Gaussian 16 software [28]. Thermal corrections were extracted from the matching uncorrected vibrational frequencies, also used to confirm the obtained structures as true minima by the lack of imaginary frequencies. The final electronic energies were refined through single-point calculations with a highly flexible 6–311++G(2df,2pd) basis set. Solvent effects were considered through the implicit SMD polarizable continuum model with all parameters for pure ethanol (ε = 24.852), giving the B3LYP/6–311++G(2df,2pd)//(SMD)/B3LYP/6–31+G(d) model employed here, being fully in line with our earlier reports [24]. As such, all computational values correspond to differences in Gibbs free energies obtained at a room temperature of 298 K and a normal pressure of 1 atm. The choice of this computational setup was additionally prompted by its success in modeling mechanisms of various antioxidants [29], and in reproducing kinetic and thermodynamic parameters of a variety of organic and enzymatic reactions [30,31,32]. According to the literature, there are several mechanisms that relate to the antioxidative properties of molecules [33]. Here, we inspected the two most frequent, and usually thermodynamically most preferred antioxidant mechanisms, namely hydrogen atom transfer (HAT), and single electron transfer (SET) that is commonly followed by proton transfer (SET-PT). All these mechanisms result in the formation of the same antioxidant radical. 

HAT is a major route where the H atom (hydrogen radical, H•) is directly transferred from an antioxidant (**M**) to a free radical accompanied by the homolytic M–H bond cleavage. The capacity of this process is governed by the M–H bond dissociation energy (BDE), calculated as:

**M**–H → **M**• + H• **BDE** = *G*(**M**•) + *G*(H•) − *G*(**M**–H).

Lower BDE values point to a lower stability of the corresponding M–H bond, suggesting that it can be easily broken. Therefore, the lower BDE parameter indicates a better antioxidant property of the investigated compound **M**.

Scavenging the free radicals can also be accomplished by donating an electron from a system **M** in the SET-PT process. This process is driven by the adiabatic ionization energy (IE) necessary to eject a single electron from **M**, calculated as:

**M** → **M**•^+^ + e^−^ IE = *G*(**M**•^+^) − *G*(**M**).

Analogously to BDE, the lower IE value signifies a better antioxidant property of a system **M**.

## 3. Results and Discussion

### 3.1. Chemistry

**Scheme 1:**
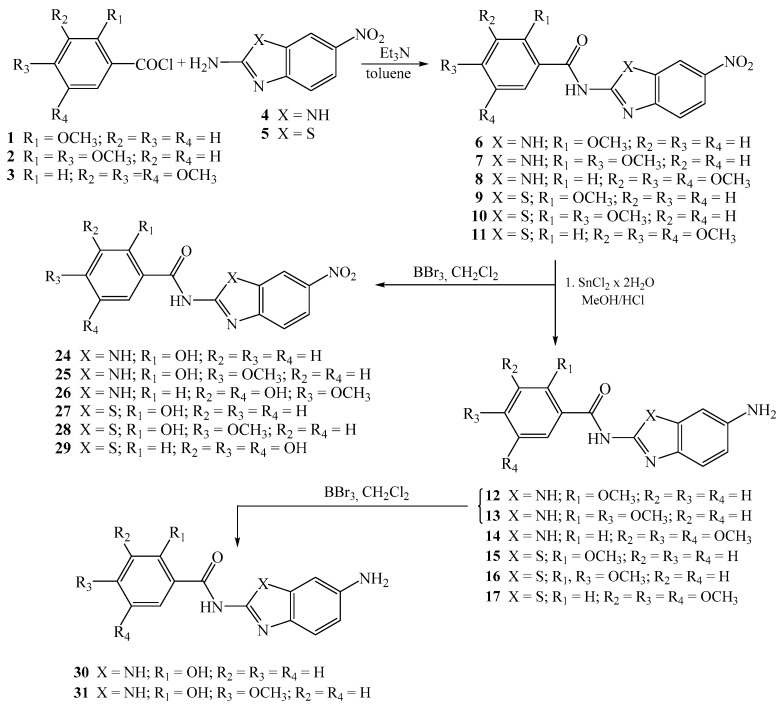
Synthesis of 2-benzimidazole/2-benzothiazole carboxamides.

**Scheme 2:**
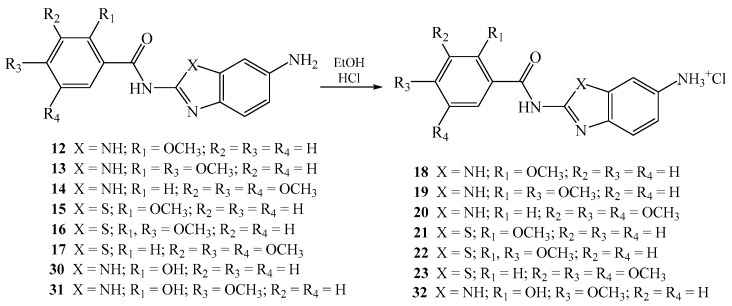
Synthesis of 2-benzimidazole/2-benzothiazole carboxamides as hydrochloride salts.

Novel nitro **6**–**11** and **24**–**29**, amino **12**–**17** and **30**–**31**, and amino protonated **18**–**23** and **32** 2-benzimidazole/2-benzothiazole carboxamides were synthesized as presented in Scheme 1 and Scheme 2, using well described and conventional organic synthetic methods. 

Starting from the corresponding benzoyl-chlorides **1–3**, in the reaction with nitro substituted 2-aminobenzimidazole/benzothiazole **4–5** in absolute toluene using triethylamine, carboxamides **6–11** were obtained in moderate reaction yields [34]. Amino substituted carboxamides **12–17** were afforded through the reduction of nitro analogues with SnCl_2_ × 2H_2_O in methanol, while their amino protonated analogues **18–23** were prepared in absolute ethanol with gaseous HCl. To obtain the matching hydroxy substituted carboxamides **24–29** with nitro groups, the removal of methoxy protecting groups was achieved with boron tribromide in absolute dichloromethane at −75 °C. 

Additionally, hydroxy substituted amino benzimidazole-2-carboxamides **30–31** were prepared by using the same method while the synthesis of amino protonated analogue **32** was accomplished with gaseous HCl in absolute ethanol. The structure of all newly prepared systems was determined by both ^1^H and APT ^13^C NMR spectroscopies and elemental analysis. NMR analysis relied on the values of H–H coupling constants and chemical shifts in the corresponding NMR spectra. Reduction of the nitro group into the amino moiety was monitored by the appearance of the signals related to amino protons in the range 5.5–6.5 ppm in the ^1^H NMR spectra.

### 3.2. Biological Evaluation

#### 3.2.1. Antiproliferative Activity in Vitro

All newly prepared compounds were first tested against HCT116, MCF-7 and H 460 cancer cell lines to assess their antiproliferative activity in vitro. The results are presented in Table 1 and are compared to a known antiproliferative agent etoposide. In addition, we selected 12 representative derivatives, which either showed the most prominent antiproliferative and/or radical scavenging activities (see Section 3.2.2.), or belong to different classes of molecules, and evaluated their cytotoxic activity on non-cancerous cells using human embryonic kidney cell line HEK 293.

The prepared systems were designed with the aim of systematically evaluating structural and electronic effects on antiproliferative features. The obtained results indicate that benzazole nuclei exert a significant effect on the studied activities. Generally, benzothiazole-2-carboxamides **15–17**, **22–23**, and **27–29** revealed a better activity relative to their benzimidazole analogues **12–14, 19–20**, and **24–26**, with the exception of the methoxy substituted nitro derivatives **6–11**, where the opposite effect was noticed. Among **6–11**, methoxy-substituted benzimidazole analogues displayed enhanced activity but without significant selectivity among tested cell lines. The only exception was provided by the most active nitro derivative **8**, having three methoxy moieties that showed the most prominent antiproliferative activity and a selective activity against HCT116 cell line in the submicromolar range (IC_50_ = 0.60 ± 0.03 µM), while being significantly less active in the non-tumor cell line (IC_50_ = 2.0 ± 0.5 µM). Surprisingly, its amino **14** and amino protonated analogue **20** did not reveal any activity towards HCT116 and H 460 cell lines, while it showed only moderate activity towards the MCF-7 cell line. A higher number of methoxy groups slightly improved the antiproliferative activity in nitro-benzimidazole derivatives **6–8**, but this was not observed with benzothiazole analogues **9–11**. Amino substituted benzimidazoles having only methoxy groups **12–14** showed a decreased activity, while their benzothiazole analogues **15–17** exhibited a slight improvement of activity relative to their nitro analogues **9–11**. There was no noteworthy influence of the amino protonated groups in benzimidazole **18–20** and benzothiazole derivatives **21–23** compared to amino substituted analogues, but some selectivity against the MCF-7 cell line still remained. Furthermore, converting one methoxy to the hydroxy group in benzimidazole derivatives **24–25**, relative to their nitro analogues **6–7**, led to the decrease of the activity. Oppositely, the introduction of one or two hydroxy groups in the structure of benzothiazole derivatives **27–29** improved the antiproliferative activity compared to its methoxy analogues **9–11**. 

In summary, the results showed that 12 selected compounds had a relatively similar cytotoxic profile in tumor cells in comparison to non-tumor cells. The exception was compound **8**, which had the most pronounced and selective activity towards HCT116 cells, while significantly lower activity (≈3 times) towards HEK293 cells. Contrary to this, the reference compound etoposide was similarly, or even more cytotoxic towards the HCT116 cell line. In addition, compound **21** which had strong antiproliferative activity towards tumor cells (IC_50_ = 3–10 µM) showed significantly lower cytotoxic activity towards HEK 293 cells (IC_50_ = 25 µM).

#### 3.2.2. Antioxidative Capacity of Benzimidazole/Benzothiazole Derivatives

To determine the antioxidant potency, and the reducing activity of the stable radical 1,1-diphenyl-picrylhydrazyl (DPPH) and ferric reducing/antioxidant power (FRAP) parameters were evaluated, respectively. Results were compared to a standard compound BHT (Table 2).

The DPPH method is based on the ability of studied systems to donate a hydrogen atom or an electron to DPPH, and it has been broadly employed to evaluate the free radical scavenging capacity of various compounds. The obtained results indicate that several compounds showed excellent DPPH quenching ability, surpassing the activity of a reference BHT (IC_50_ = 25 ± 4 µM). The most pronounced antioxidative capacity is shown by the trimethoxy substituted benzothiazole-2-carboxamide **23** having the amino protonated group (IC_50_ = 1.5 ± 0.5 µM) and trihydroxy substituted benzothiazole-2-carboxamide **29** bearing nitro group (IC_50_ = 2.00 ± 0.15 µM). Their analogue having the amino group **17** showed a lower quenching ability (IC_50_ = 40.4 ± 0.4 µM). One notices a persistent trend with systems bearing an amino protonated group, being more active and showing the improvement of the radical scavenging activity relative to their amino substituted analogues. For example, methoxy substituted benzothiazole-2-carboxamide **21** (IC_50_ = 12.0 ± 1.1 µM) displayed the significant improvement of radical trapping activity compared to its amino analogue **15**. Nitro substituted **6–11**, both benzimidazole/benzothiazole derivatives, showed very low quenching ability among all tested compounds in comparison to their amino analogues. Furthermore, the obtained results pointed to the influence of the variable number of the methoxy groups. Therefore, amino substituted compounds having three methoxy groups showed better quenching ability among studied systems.

By using the FRAP assay, we investigated the reducing power of investigated systems, which was monitored by the corresponding changes in the absorbance at 593 nm. This method is based on the ability of systems to reduce the ferric tripyridyl triazine complex (TPTZ) to the ferrous state (Fe^2+^), which can be seen by an intense blue color. Results in Table 2 revealed that most of the tested compounds showed lower reducing power relative to the BHT standard. The most promising feature was shown by the trihydroxy substituted benzothiazole-2-carboxamide **29** having the nitro group (6139.2 ± 3.0 mmol Fe^2+^/mmol_C_), which is threefold higher in comparison to standard BHT. Good reducing ability was shown by the trimethoxy substituted benzimidazole-2-carboxamide **14** bearing the amino group (1102.5 ± 14.1 mmolFe^2+^/mmol_C_). Amino and amino protonated analogues **6–11** showed improved reducing power with very little differences among them, indicating that their protonation state is less important here. Additionally, the increased number of methoxy groups, as well as the introduction of one or two hydroxy groups, did not cause any improvement in the measured reducing power.

#### 3.2.3. Antioxidant Ability in Cells

**Figure 1:**
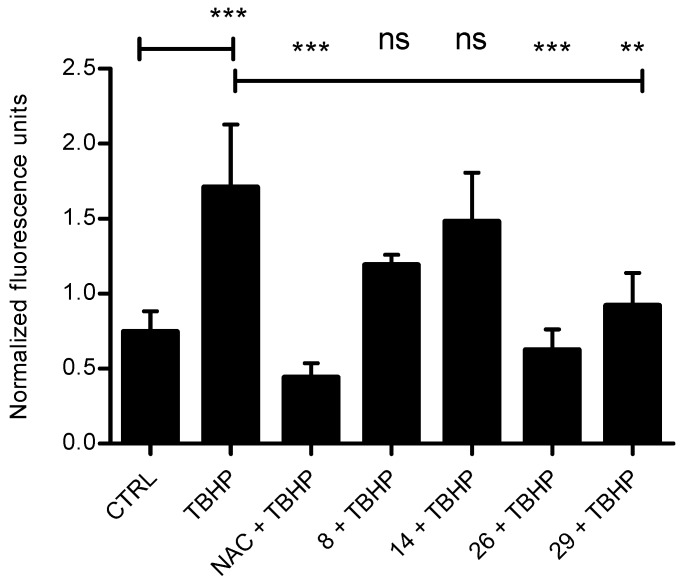
Antioxidative activity of selected systems. HCT 116 cells were treated with the combination of *tert*-Butyl hydroperoxide (TBHP, 100 uM) and *N*-Acetyl-l-cysteine (NAC, 50 mM) or tested compounds **8**, **14**, **26**, and **29** (10 µM) for 1 h. The level of reactive oxygen species (ROS) was measured with fluorescent dye 2′,7′-dichlorodihydrofluorescein diacetate (DCFH-DA) using fluorimeter. Treatment with 100 uM TBHP alone was used as a control for ROS induction. Data presented here are the results of three independent measurements, done in triplicates. One-way ANOVA with Tukey’s post-hoc test was used for statistical analysis, ** *p* < 0.01, *** *p* < 0.001. ns stands for “not significant”.

In order to test the antioxidant activity of selected systems in tumor cells (Figure 1), we treated HCT 116 cells with *tert*-Butyl hydroperoxide (TBHP), a substance commonly used for inducing oxidative stress in cells and tissues, alone or in a combination with a known antioxidative agent *N*-Acetyl-l-cysteine (NAC) or tested compounds [35]. We measured the formation of oxidative stress by-products using 2′,7′-dichlorodihydrofluorescein diacetate (DCFH-DA). We selected system **8** showing pronounced antiproliferative/cytotoxic activity, but no antioxidant capacity in DPPH/FRAP assays, **29** showing exceptionally pronounced antioxidative activity in DPPH/FRAP tests, **14** showing negligible antiproliferative but strong antioxidant capacity in both DPPH/FRAP assays, and **26** showing similarly strong antiproliferative and radical trapping activity in DPPH assay, but rather modest reducing power in FRAP assay. None of the compounds influenced the basal level of ROS in the cells. Interestingly, when oxidative stress was induced, systems **26** and **29** significantly reduced the ROS levels, comparably to NAC, thus confirming their antioxidant capacity obtained in DPPH/FRAP assays. On the other hand, no effect was obtained upon treatment with either **8** or **14** (Figure 1). We additionally tested the impact of the selected compounds on cellular and mitochondrial ROS production in HCT 116 cells using fluorescent dyes, DCFH-DA for cellular and MitoSOX for mitochondrial ROS detection (Supplementary Materials, Figures S53 and S54). 

The results confirmed that the compounds did not induce oxidative stress in cells or mitochondria after 1 h of treatment. Consequently, the antiproliferative activity of tested compounds is not related to their ability to induce oxidative stress. Furthermore, compound **14** has negligible cytotoxic, as well as antioxidative activity in cells, unlike in vitro assays. It is known that antioxidant activities of compounds differ in diverse assays, due to different specificities for different conditions, e.g., pH, solvents, or substances hydrophobicity [36]. This also might be attributed to the low penetrating ability of **14** through the cell membrane. 

### 3.3. Computational Analysis

**Figure 2:**
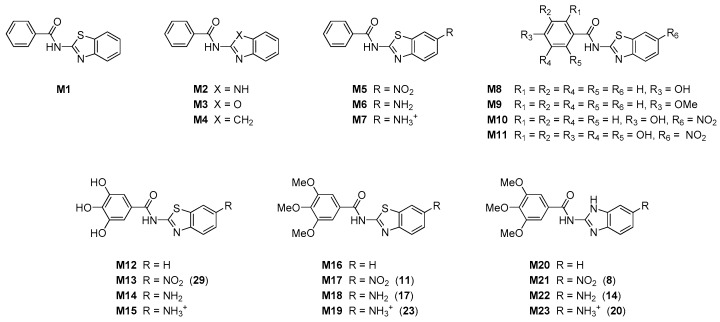
Schematic representation of molecules examined computationally, with numbers in round brackets relating to experimentally studied systems.

Computational analysis was performed to offer a further insight into the structure and properties of investigated compounds, and to provide the rationalization of the measured antioxidant features. Given that experimentally characterized systems are structurally very similar and show a relatively narrow span of antioxidant activities, we decided to proceed with a set of model systems **M1**–**M23** (Figure 2), chosen to closely represent the examined set of molecules **6**–**32**. This would allow enough structural and electronic information to offer some general conclusions about the studied compounds in order to aid in the design of even more potent antioxidants based on the employed organic framework.

The calculated bond dissociation energies (BDE) and ionization energies (IE) for **M1**–**M23** are given in Table 3. Both sets of data consider thermodynamic aspects of their reactivities, while overlooking kinetic features [25]. Yet, this is a sensible assumption, since, within such a set of similar systems, it is plausible to expect that kinetic aspects of H-atom or electron transfer reactions are to a large degree similar and are not crucial for determining the antioxidative activities, as already discussed in the literature [37,38]. 

As clarified in the Computational Details section (see later), systems having lower BDEs show better antioxidant features through the H-atom transfer pathway, while lower IE values indicate better antioxidants via the single electron transfer mechanism. Systems with protonated amino groups have lower BDEs than their unionized analogues as a rule. This is seen in all matching pairs, **M7**–**M6**, **M15**–**M14**, **M19**–**M18**, and **M23**–**M22**, where protonated analogues have between 17 and 30 kcal mol^−1^ lower BDEs than neutrals. This is sensible, since it is easier to abstract an H-atom from a charged and more acidic cationic system than it is from a neutral compound. Additionally, following the H-atom abstraction in the protonated system, the formed radical cation can delocalize both the positive charge and the unpaired electron spin density into the attached aromatic system, which both contribute to lowering the BDEs [24], as already noticed by Liu and Bordwell [39]. For example, BDEs for the monocationic Me–NH_3_^+^, c-C_6_H_11_–NH_3_^+^, and Ph–NH_3_^+^ in acetonitrile are 114.6, 113.6, and 84.9 kcal mol^−1^ [39], which signifies the importance of the aromatic fragment. Analogously, neutral molecules are easier to ionize since it is less demanding to eject an electron from a neutral **M** to get a radical cation **M**•**^+^** than it is from an already cationic **MH^+^** to give a doubly charged radical **MH•^++^**. Given that, for all compounds, BDE is always lower than IE (Table 3), we will focus on differences in BDEs unless stated otherwise. This suggests the H-atom transfer as the likely major antioxidative pathway, in agreement with earlier reports on various phenolic antioxidants [37,40,41,42] or compounds with other X–H bond energetics (X = C, N, O, S) [43]. 

We will start the analysis with the unsubstituted parent **M1**, which will serve as a reference point for other derivatives. Its BDE value is 82.7 kcal mol^−1^, related to the abstraction of the amide N–H hydrogen atom. Interestingly, this is significantly lower than that for the analogous bisphenylamide, Ph–CO–NH–Ph, for which our earlier calculations gave BDE = 85.3 kcal mol^−1^ [24]. This suggests that amide *N*-benzothiazole is a more suitable building block than *N*-phenyl in tailoring more efficient antioxidants, which justifies the employed design strategy. The reason for that is a considerable delocalization of the unpaired electron-density within the thiazole fragment following the H-atom abstraction, which works towards reducing the BDE. The latter is evident in the geometric parameters, where bonds involving N(amide)–C(benzothiazole), C(benzothiazole)–S, and C(benzothiazole)–N change from 1.382, 1.772, and 1.301 Å in neutral **M1**, respectively, to 1.305, 1.818, and 1.363 Å in radical, in the same order. At the same time, the matching N(amide)–C(amide) and C(amide)–O(amide) bonds undergo much smaller change, from 1.376 and 1.237 Å in neutral **M1**, being practically intact at 1.396 and 1.240 Å in radical, strongly indicating that the benzothiazole moiety takes a predominant role in accommodating an unpaired electron in the radical. 

This also suggests that substituents at this aromatic fragment will have a stronger influence on the antioxidative features than those placed at the phenyl group on the other side of the system. This is evident by considering, for example, BDEs for **M9** and **M6**, where the electron-donating methoxy group placed on phenyl in **M9** reduces the BDE (**M1**) value only by 0.5 kcal mol^−1^, while the analogous attachment of the electron-donating amino group on benzothiazole in **M6** exerts the same BDE-lowering effect, yet by as much as 7.1 kcal mol^−1^. Still, BDE (**M1**) = 82.7 kcal mol^−1^ is among the highest here, indicating that **M1** itself is a very poor antioxidant. Furthermore, our calculations for the reference **BHT** give BDE (**BHT**) = 65.8 kcal mol^−1^ in ethanol, which appears in the right range as it is well-matched to values of 79.9 [44], 76.9 [45], and 72.4 [46] kcal mol^−1^ in heptane, benzene, and toluene, respectively. A notably lower BDE (**BHT**) in ethanol is consistent with a noted reduction in the O–H BDE values with the solvent polarity [37] that follows a trend in the matching dielectric constants of ε = 1.9, 2.2, 2.4, and 16.2, in the same order. This further confirms the poor antioxidant features of **M1** as its BDE value is 16.9 kcal mol^−1^ higher, thus less favorable than that for **BHT**. 

To evaluate the suitability of the benzothiazole moiety in **M1**, we carried out calculations on its benzoimidazole (**M2**), benzoxazole (**M3**), and indole (**M4**) analogues. It turns out that all of these show improved antioxidative features, especially the indole system **M4**, whose BDE value is 10.1 kcal mol^−1^ lower than that of **M1**. These results direct the attention toward utilizing these organic skeletons in future synthetic attempts, which will be addressed in our subsequent studies. This trend nicely explains why **14** is around four times a more potent antioxidant than **17**, and is in line with results for benzoimidazoles **M20**–**M23** that all have lower BDEs than their analogous benzothiazoles **M16**–**M19**. Addition of the nitro group, as in **M5**, increases BDE to 86.0 kcal mol^−1^, while the similar substitution with the amino moiety, as in **M6**, reduces BDE to 75.6 kcal mol^−1^, being 7.1 kcal mol^−1^ lower than BDE (**M1**), thereby facilitating its antioxidant feature. This emphasizes the favorable effect of the electron-donating moieties, also seen with methoxy derivatives later. This is consistent with previous reports on the ability of electron-donating groups to reduce BDEs, as, for example, *p*-NMe_2_ lowers the BDE of phenol by 10.1 kcal mol^−1^ [47]. Furthermore, Jonsson et al. reported N–H BDEs for aniline, 4-CN-aniline, and 4-OMe-aniline of 89.1, 91.8, and 87.2 kcal mol^−1^ in water, respectively [48], firmly tying in with the mentioned observation. In addition, this notion rationalizes why amino-substituted **30** and **31** are better antioxidants than their nitro analogues **24** and **25**, being further supported by lower BDEs calculated for **M6**, **M14**, **M18**, and **M22** relative to **M5**, **M13**, **M17**, and **M21**, respectively. Particularly noteworthy is the last pair, where the amino-substituted **M22** is almost 15 kcal mol^−1^ a more potent antioxidant than the nitro-substituted **M21**, which is significant. 

One notices that in **M6** the most favorable site for the H-atom abstraction remains the amide moiety, as the calculated BDE for the aniline N–H unit is 77.8 kcal mol^−1^, thus is 2.2 kcal mol^−1^ higher. Still, a simple protonation of **M6** to **M7** exerts a dramatic influence on its ability to donate a H- atom, as the calculated BDE (**M7**) reduces to 48.3 kcal mol^−1^. To put this number in a proper context, let us recall that BDE (**BHT**) = 65.8 kcal mol^−1^, making **M7** a significantly (17.5 kcal mol^−1^) more potent antioxidant. This trend is also evident in all other amino-protonated derivatives, systems **M7**, **M15**, **M19**, and **M23** are significantly more efficient in removing the H-atom than their unionized analogues **M6**, **M14**, **M18**, and **M22** (Table 3). This nicely rationalizes why systems **18**–**23** are, as a rule, much better antioxidants than **12**–**17** (Table 2), with **23** having the lowest DPPH value of 1.5 ± 0.5 μM. With this in mind, it remains a pity that all of our synthetic efforts to prepare amino-protonated derivatives of the most potent nitro-substituted antioxidants studied here have failed, leaving this as a synthetic challenge for future studies. In **M7**, the H-atom abstraction occurs on the cationic amino moiety, with the calculated BDE for the amide group being much higher at 92.5 kcal mol^−1^. This is a persistent trend in all amino-protonated derivatives, which owe their pronounced antioxidative activity to the ease of the H-abstraction from the protonated –NH_3_^+^ moiety. Insertion of the electron-donating methoxy groups on the phenyl ring does not exert a significant effect on the calculated parameters. For example, *p*-OMe group in **M9** reduces BDE (**M1**) only by 0.5 kcal mol^−1^ to BDE (**M9**) = 82.2 kcal mol^−1^. Furthermore, analogous tri-substitution, as in **M16**, even reverses this trend leading to a less potent compound with BDE (**M16**) = 83.3 kcal mol^−1^. Thus, one concludes that electron-donating substituents on the phenyl ring are not significantly (if at all) promoting the antioxidative features, which is experimentally supported, for example, by **13** and **26** having higher DPPH values than **31** and **29**, respectively. On the other hand, the introduction of the hydroxy group –OH exerts a different, but favorable behavior. Monosubstituted **M8** is already by 4.8 kcal mol^−1^ a better antioxidant than **M1**, with a necessary observation that the most favorable site of the H-atom abstraction moves to the hydroxy moiety. This is because the O–H group is typically easier to undergo a homolytic cleavage than the amide N–H [49], thus exerting a strong emphasis for the design of more effective antioxidants. This notion fully agrees with demonstrated antioxidative features of many phenols and polyhydroxy aromatics described elsewhere [50]. Here, this is evident in systems **26**, **29**–**31**, which are shown as much more potent than analogous **8**, **11**–**13**, and computationally reproduced for **M12**–**M14**, having significantly lower BDEs than **M16**–**M18**. Still, the introduction of the nitro group to **M8**, as in **M10**, lowers its antioxidative capacity by 1.1 kcal mol^−1^, in line with the already presented negative effect of the electron-withdrawing substituents. Interestingly, poly-hydroxylation of **M10** overcomes the unfavorable effect of the nitro group and gives system with BDE (**M11**) = 70.4 kcal mol^−1^, being much lower than BDE (**M8**) = 77.9 kcal mol^−1^, which features a network of hydrogen bonds among the –OH groups in both neutral and radical system, and loses its H-atom from the aromatic C3-position. Still, according to previous reports, multiple hydrogen bonds in antioxidants work toward preventing an efficient cleavage of the matching O–H bonds, unless being only a favorable five-center interaction among vicinal groups as in tri-substituted **M12**–**M15** [47,51]. 

A particular case is provided by **M12**, bearing three hydroxy groups on the same phenyl ring. Its BDE (**M12**) = 64.3 kcal mol^−1^ is much lower than in all neutral systems discussed so far. The reason for its increased antioxidative potency is because, in **M12**•, the formed *para*-phenoxy radical center forms stable hydrogen bonding pattern with both neighboring hydroxy moieties at the [O•·····H–O] distances of 2.229 and 2.214 Å, which stabilize the system. This structural element has already been elucidated as accountable for the increased radical scavenging ability of some naturally occurring antioxidants having two or three aromatic hydroxy groups [47], such as gallic acid, for which the calculated BDE is 77.0 kcal mol^−1^, and is much lower than BDE (phenol) = 82.9 kcal mol^−1^ [50] or in the related bisphenylamides [24]. The rather low BDEs for these catechols are due to the electron-donating effect of the second (and third) OH group and to the increase in strength (by several kcal mol^−1^) [49] of the intramolecular hydrogen bonding on going from the neutral system to the radical [52]. Additionally, a close vicinity of hydroxy groups is essential for the phenoxy radical stability as, for example, the calculated BDE for 1,2,3-trihydroxybenzene is by 15 kcal mol^−1^ lower than that for its 1,3,5-trihydroxy analogue in toluene [46]. The nitro derivative of **M12**, namely system **29** (or **M13**) has been underlined here as the most potent antioxidant. Yet, our calculations show this is despite the fact that the introduction of the nitro group increases the BDE value of **M12** by 0.8 kcal mol^−1^ to 65.1 kcal mol^−1^. 

Regarding future directions, let us once again recall the demonstrated favorable effect of the electron-donating amino groups, which enhance the antioxidative capacities. In this context, it should be mentioned that the calculated BDE for the amino analogue **M14** is reduced to 63.8 kcal mol^−1^, while that for the amino-protonated derivative **M15** is as low as 46.4 kcal mol^−1^ (Table 3). With this, the latter value would likely drop the measured DPPH value for **M15** to a nano range, making its synthesis strongly suggested. Nevertheless, the design of multifunctional biologically active systems presented here clearly led to compounds exhibiting significantly improved antioxidative activities relative to analogous bisphenylamides reported earlier [24], thus justifying the employed strategy. It is in that context that we are convinced that the presented results offer useful guidelines in designing improved systems and direct the attention towards employing the benzimidazole and benzothiazole scaffolds in this direction.

## 4. Conclusions

This work reports on the synthesis, computational analysis, and biological evaluation of various benzimidazole/benzothiazole-2-carboxamides substituted with a variable number of methoxy and/or hydroxy moieties, and bearing nitro, amino, or amino protonated groups. The prepared systems were evaluated for their in vitro antiproliferative activity against three cancer and one non-tumor cell line using etoposide as a standard drug, while their antioxidative capacity was determined by measuring the radical scavenging ability and reducing power. Most of the tested compounds revealed modest antiproliferative activity without a significant selectivity between tested cell lines. However, the most prominent activity was demonstrated by the trimethoxy substituted benzimidazole-2-carboxamide **8** bearing nitro group, which was particularly selective towards the HCT116 cell line (IC_50_ = 0.60 ± 0.03 µM), while having significantly lower activity towards HEK293 cells (IC_50_ = 2.0 ± 0.3 µM). In contrast, radical scavenging assay showed that systems with amino protonated group are more active over their unionized analogues, which was rationalized by computations. Obtained results are fully in line with earlier reports on benzamide derivatives. 

Trihydroxy substituted benzothiazole-2-carboxamide **29** revealed the most promising radical scavenging capacity (IC_50_ = 2.00 ± 0.15 µM; mmolFe^2+^/mmol_C_ 6139.2 ± 3.0). Similarly, strong antioxidative and radical scavenging activities were demonstrated by **26**, which were also confirmed in tumor cells. Computational analysis showed that **29** owes its pronounced antioxidative capacity to the stabilizing hydrogen bond involving the formed *para*-phenoxy radical center with the neighboring hydroxy groups in **29**•. A strong antiproliferative, as well as antioxidant activity of **26** and **29** should be further studied in order to delineate more precise biological mechanisms of their activity. 

Based on the SAR study, system **8** as the most prominent antiproliferative agent, and **29** as the most promising antioxidant were chosen as lead compounds for further structure modifications of the benzazole-2-carboxamide scaffold to afford more efficient antioxidants and/or systems with the antiproliferative activity. At least as the antioxidants are concerned, this conclusion was firmly supported by the computational analysis, which confirmed **29** as a good starting point toward even more effective compounds through several pathways including the substitution of its nitro group with amino or amino-protonated moieties in particular, and the replacement of the benzothiazole nuclei with either benzimidazole, benzoxazole, or indole scaffolds. 

## Figures and Tables

**Table 1:** IC_50_^a^ values for tested compounds (in µM).

Cpd	R	R_1_	R_2_	R_3_	R_4_	X	IC_50_ (µM)			
							HCT116	MCF–7	H 460	HEK 293
**6**	NO_2_	OMe	H	H	H	NH	6 ± 1	4.0 ± 0.3	6 ± 5	3.0 ± 0.6
**7**	NO_2_	OMe	H	OMe	H	NH	3.0 ± 0.2	2.0 ± 0.9	4 ± 2	2.0 ± 0.3
**8**	NO_2_	H	OMe	OMe	OMe	NH	0.60 ± 0.03	1.0 ± 0.2	2.0 ± 0.2	2.0 ± 0.5
**9**	NO_2_	OMe	H	H	H	S	44 ± 25	9 ± 1	46 ± 3	n.t. ^b^
**10**	NO_2_	OMe	H	OMe	H	S	9 ± 7	5 ± 2	20 ± 3	5.0 ± 1.0
**11**	NO_2_	H	OMe	OMe	OMe	S	37.0 ± 0.8	17 ± 9	23 ± 4	n.t.
**12**	NH_2_	OMe	H	H	H	NH	48 ± 4	53 ± 9	39 ± 11	n.t.
**13**	NH_2_	OMe	H	OMe	H	NH	17 ± 8	20 ± 4	24 ± 8	n.t.
**14**	NH_2_	H	OMe	OMe	OMe	NH	76 ± 24	43 ± 15	>100	43.0 ± 2.0
**15**	NH_2_	OMe	H	H	H	S	6 ± 4	3 ± 2	3.0 ± 0.9	n.t.
**16**	NH_2_	OMe	H	OMe	H	S	6 ± 1	5.0 ± 0.3	43 ± 29	11.0 ± 8.0
**17**	NH_2_	H	OMe	OMe	OMe	S	28 ± 4	5 ± 3	≥100	n.t.
**18**	NH_3_^+^Cl^−^	OMe	H	H	H	NH	33 ± 7	23 ± 2	36 ± 8	n.t.
**19**	NH_3_^+^Cl^−^	OMe	H	OMe	H	NH	≥100	34 ± 24	>100	n.t.
**20**	NH_3_^+^Cl^−^	H	OMe	OMe	OMe	NH	≥100	34 ± 30	>100	n.t.
**21**	NH_3_^+^Cl^−^	OMe	H	H	H	S	10 ± 8	3.0 ± 0.7	4.0 ± 0.1	25.0 ± 1.5
**22**	NH_3_^+^Cl^−^	OMe	H	OMe	H	S	8 ± 2	3.0 ± 0.2	50 ± 8	11.0 ± 1.0
**23**	NH_3_^+^Cl^−^	H	OMe	OMe	OMe	S	50 ± 11	12 ± 24	34 ± 9	n.t.
**24**	NO_2_	OH	H	H	H	NH	61.0 ± 0.1	42 ± 7	42 ± 20	n.t.
**25**	NO_2_	OH	H	OMe	H	NH	31 ± 4	28 ± 6	18.00 ± 0.01	n.t.
**26**	NO_2_	H	OH	OMe	OH	NH	4.0 ± 0.1	3 ± 1	3.0 ± 0.5	3.0 ± 0.7
**27**	NO_2_	OH	H	H	H	S	6 ± 1	2.0 ± 0.6	3.0 ± 0.2	3.0 ± 1.0
**28**	NO_2_	OH	H	OMe	H	S	7.0 ± 0.4	10 ± 8	8 ± 1	4.0 ± 2.0
**29**	NO_2_	H	OH	OH	OH	S	7 ± 1	4.0 ± 0.7	2 ± 1	6.0 ± 4.0
**30**	NH_2_	OH	H	H	H	NH	46.0 ± 0.2	30.0 ± 0.8	>100	n.t.
**31**	NH_2_	OH	H	OMe	H	NH	37 ± 7	≥100	75 ± 27	n.t.
**32**	NH_3_^+^Cl^−^	OH	H	OMe	H	NH	50 ± 13	80 ± 4	≥100	n.t.
**Etoposide**							5 ± 2	1.0 ± 0.7	0.10 ± 0.04	1.6 ± 0.4

^a^ IC_50_ values signify a concentration that causes 50% growth inhibition. ^b^ not tested (n.t.).

**Table 2:** IC_50_ values for 1,1-diphenyl-picrylhydrazyl (DPPH) free radical scavenging and ferric reducing/antioxidant power (FRAP) activities.

Cpd	R	R_1_	R_2_	R_3_	R_4_	X	FRAP ^a^ mmolFe^2+^/mmol_C_	DPPH ^b^ IC_50_/μM
**6**	NO_2_	OCH_3_	H	H	H	NH	123.5 ± 2.8	– ^c^
**7**	NO_2_	OCH_3_	H	OCH_3_	H	NH	139.6 ± 16.9	–
**8**	NO_2_	H	OCH_3_	OCH_3_	OCH_3_	NH	89.7 ± 6.2	–
**9**	NO_2_	OCH_3_	H	H	H	S	6.7 ± 0.6	–
**10**	NO_2_	OCH_3_	H	OCH_3_	H	S	–	–
**11**	NO_2_	H	OCH_3_	OCH_3_	OCH_3_	S	155.5 ± 3.0	–
**12**	NH_2_	OCH_3_	H	H	H	NH	256.3 ± 2.8	28.90 ± 1.03
**13**	NH_2_	OCH_3_	H	OCH_3_	H	NH	238.4 ± 2.1	17.71 ± 2.81
**14**	NH_2_	H	OCH_3_	OCH_3_	OCH_3_	NH	1102.5 ± 14.1	10.70 ± 0.23
**15**	NH_2_	OCH_3_	H	H	H	S	163.4 ± 4.7	–
**16**	NH_2_	OCH_3_	H	OCH_3_	H	S	203.6 ± 5.8	–
**17**	NH_2_	H	OCH_3_	OCH_3_	OCH_3_	S	235.4 ± 2.8	40.4 ± 0.4
**18**	NH_3_^+^Cl^−^	OCH_3_	H	H	H	NH	267.2 ± 5.0	9.850 ± 0.003
**19**	NH_3_^+^Cl^−^	OCH_3_	H	OCH_3_	H	NH	259.1 ± 0.6	13.3 ± 0.8
**20**	NH_3_^+^Cl^−^	H	OCH_3_	OCH_3_	OCH_3_	NH	267.4 ± 1.4	8.06 ± 0.03
**21**	NH_3_^+^Cl^−^	OCH_3_	H	H	H	S	214.3 ± 3.4	12.0 ± 1.1
**22**	NH_3_^+^Cl^−^	OCH_3_	H	OCH_3_	H	S	244.2 ± 3.0	4.00 ± 0.04
**23**	NH_3_^+^Cl^−^	H	OCH_3_	OCH_3_	OCH_3_	S	241.3 ± 1.6	1.5 ± 0.5
**24**	NO_2_	OH	H	H	H	NH	39.1 ± 2.0	–
**25**	NO_2_	OH	H	OCH_3_	H	NH	150.4 ± 3.4	–
**26**	NO_2_	H	OH	OCH_3_	OH	NH	244.9 ± 4.8	8.1 ± 1.9
**27**	NO_2_	OH	H	H	H	S	–	–
**28**	NO_2_	OH	H	OCH_3_	H	S	39.8 ± 1.6	–
**29**	NO_2_	H	OH	OH	OH	S	6139.2 ± 3.0	2.00 ± 0.15
**30**	NH_2_	OH	H	H	H	NH	272.6 ± 7.1	42.5 ± 0.5
**31**	NH_2_	OH	H	OCH_3_	H	NH	258.4 ± 6.4	9.2 ± 0.6
**32**	NH_3_^+^Cl^−^	OH	H	OCH_3_	H	NH	304.3 ± 6.4	29.5 ± 0.1
**BHT**							2089 ± 60	25 ± 4

^a^ Values are presented as means ± standard deviation. Values lower than those for a standard BHT system indicate more potent antioxidative activity; ^b^ Results collected after 30 min of the incubation of tested compounds with 100 μM DPPH; ^c^ Very low activity.

**Table 3:** Bond dissociation energies (BDE) and ionization energies (IE) in ethanol calculated by the B3LYP/6–311++G(2df,2pd)//(SMD)/B3LYP/6–31+G(d) model (in kcal mol^−1^).

System	BDE	Site of the M–H Cleavage	IE
**M1**	82.7	amide N–H	174.3
**M2**	81.0	amide N–H	169.3
**M3**	79.4	amide N–H	177.3
**M4**	72.6	C3-indole C–H	170.3
**M5**	86.0	amide N–H	186.8
**M6**	75.6	amide N–H	154.8
**M7**	48.3	benzothiazole N–H	247.3
**M8**	77.9	C4-phenolic O–H	171.8
**M9**	82.2	amide N–H	170.3
**M10**	79.0	C4-phenolic O–H	183.6
**M11**	70.4	C3-phenolic O–H	172.6
**M12**	64.3	C4-phenolic O–H	170.4
**M13**	65.1	C4-phenolic O–H	181.2
**M14**	63.8	C4-phenolic O–H	151.7
**M15**	46.4	benzothiazole N–H	227.6
**M16**	83.3	amide N–H	166.7
**M17**	86.5	amide N–H	173.2
**M18**	75.1	amide N–H	152.0
**M19**	45.9	benzothiazole N–H	212.3
**M20**	82.9	amide N–H	162.3
**M21**	86.6	amide N–H	171.8
**M22**	71.8	amide N–H	146.2
**M23**	45.5	benzene N–H	213.7

## Supplementary Materials

The following are available online at https://www.mdpi.com/2076-3921/8/10/477/s1, Figures S1–S52: NMR spectra of novel compounds, Figure S53: Impact of compounds on cellular ROS production, Figure S54: Impact of compounds on mitochondrial ROS production; Materials and methods: Cell culturing, Cellular ROS measurement assay, Mitochondrial ROS measurement assay.
