# Supplementary material for: Experimental and Computational Study of the Antioxidative Potential of Novel Nitro and Amino Substituted Benzimidazole/Benzothiazole-2-Carboxamides with Antiproliferative Activity
Source: Antioxidants (Basel). 2019 Oct 12;8(10):477. doi: 10.3390/antiox8100477 (PMC6826492; doi:10.3390/antiox8100477)
Supplement: Supplementary file 1 [file antioxidants-08-00477-s001.pdf]

Supporting Information

Experimental and Computational Study of the Antioxidative Potential of Novel Nitro and Amino Substituted Benzimidazole/Benzothiazole-2-Carboxamides with Antiproliferative Activity

Maja Cindrić[1], Irena Sović[1], Marija Mioč[2], Lucija Hok[3], Ida Boček[1], Petra Roškarić[1], Kristina Butković[1], Irena Martin-Kleiner[2], Kristina Starčević[4], Robert Vianello[3*], Marijeta Kralj[2*] and Marijana Hranjec[1*]

[1] Department of Organic Chemistry, Faculty of Chemical Engineering and Technology, University of Zagreb, Marulićev trg 19, HR-10000 Zagreb, Croatia

[2] Division of Molecular Medicine, Ruđer Bošković Institute, Bijenička cesta 54, HR-10000 Zagreb, Croatia

[3] Division of Organic Chemistry and Biochemistry, Ruđer Bošković Institute, Bijenička cesta 54, HR-10000 Zagreb, Croatia

[4] Department of Chemistry and Biochemistry, Faculty of Veterinary Medicine, University of Zagreb, Heinzelova 55, HR-10000 Zagreb, Croatia

* Correspondence: robert.vianello@irb.hr (R.V.); marijeta.kralj@irb.hr (M.K.); mhranjec@fkit.hr (M.H.)

Contents of Supporting Information:

1. Figures S1-S52 are NMR spectra of novel compounds (pages S2–S27)
2. Figure S53: Impact of compounds on cellular ROS production (page S28)
3. Figure S54: Impact of compounds on mitochondrial ROS production (page S28)
4. Materials and methods: *Cell culturing, Cellular ROS measurement assay , Mitochondrial ROS measurement assay* (page S29)

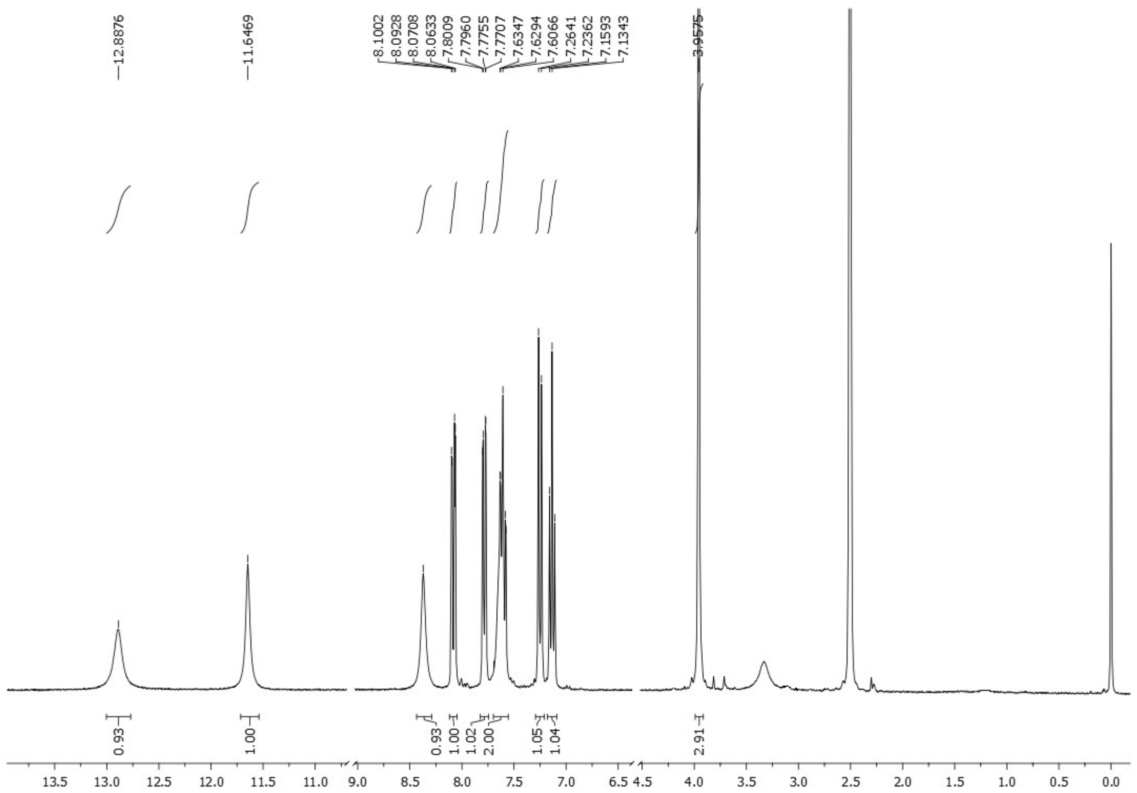

Figure S1. ^{1}H NMR spectrum (DMSO-d_6, 300 MHz) of ***2-methoxy-N-[5(6)-nitrobenzimidazol-2-yl]benzamide 6***.

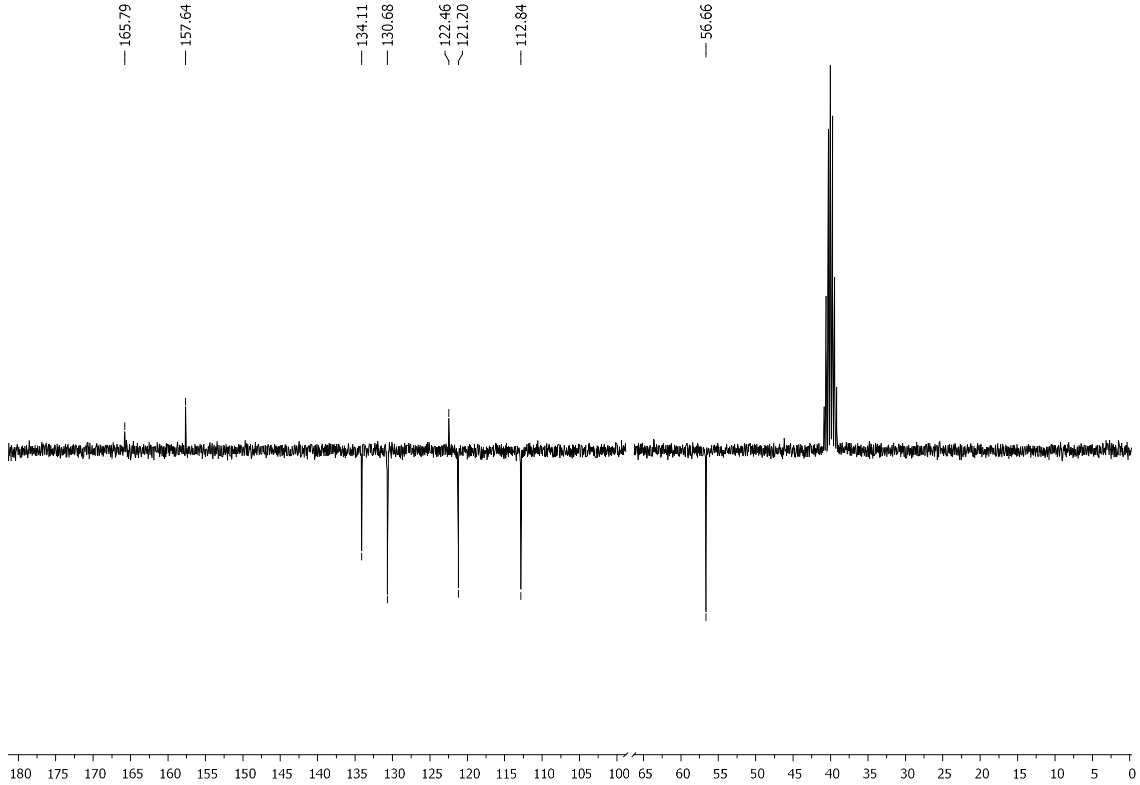

Figure S2. ^{13}C NMR spectrum (DMSO-d_6, 75 MHz) of ***2-methoxy-N-[5(6)-nitrobenzimidazol-2-yl]benzamide 6***.

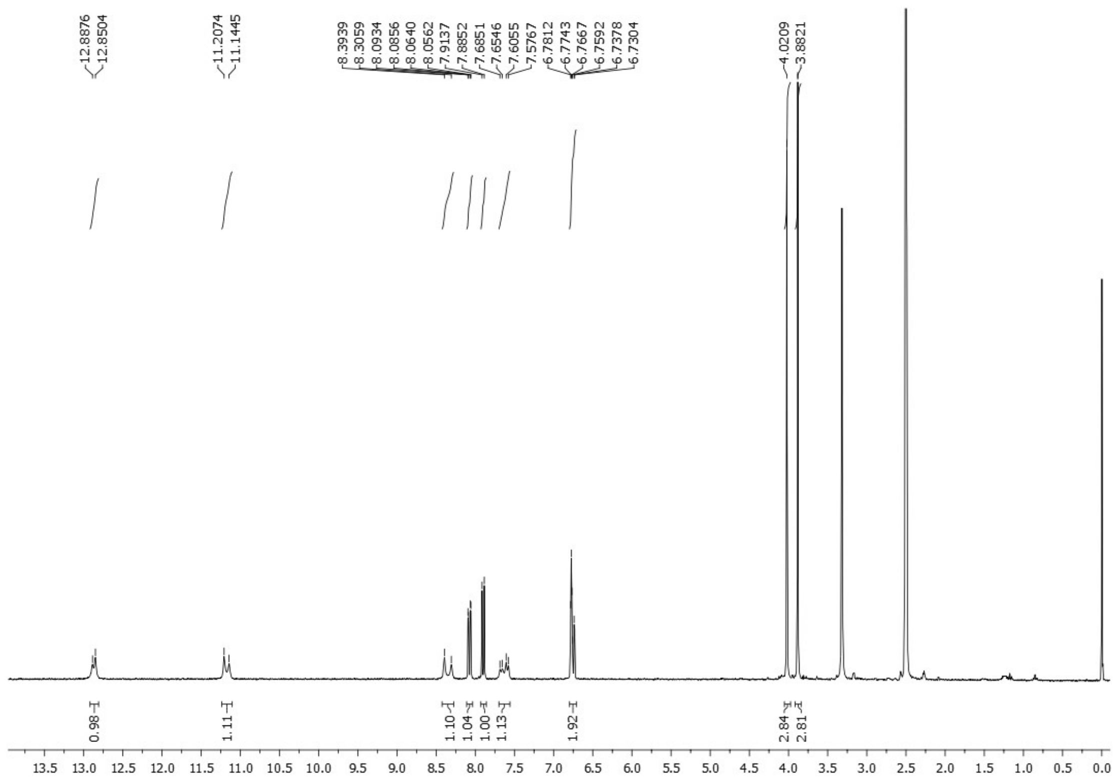

Figure S3. ^{1}H NMR spectrum (DMSO-d_6, 300 MHz) of ***2,4-dimethoxy-N-[5(6)-nitrobenzimidazol-2-yl]benzamide*** *7*.

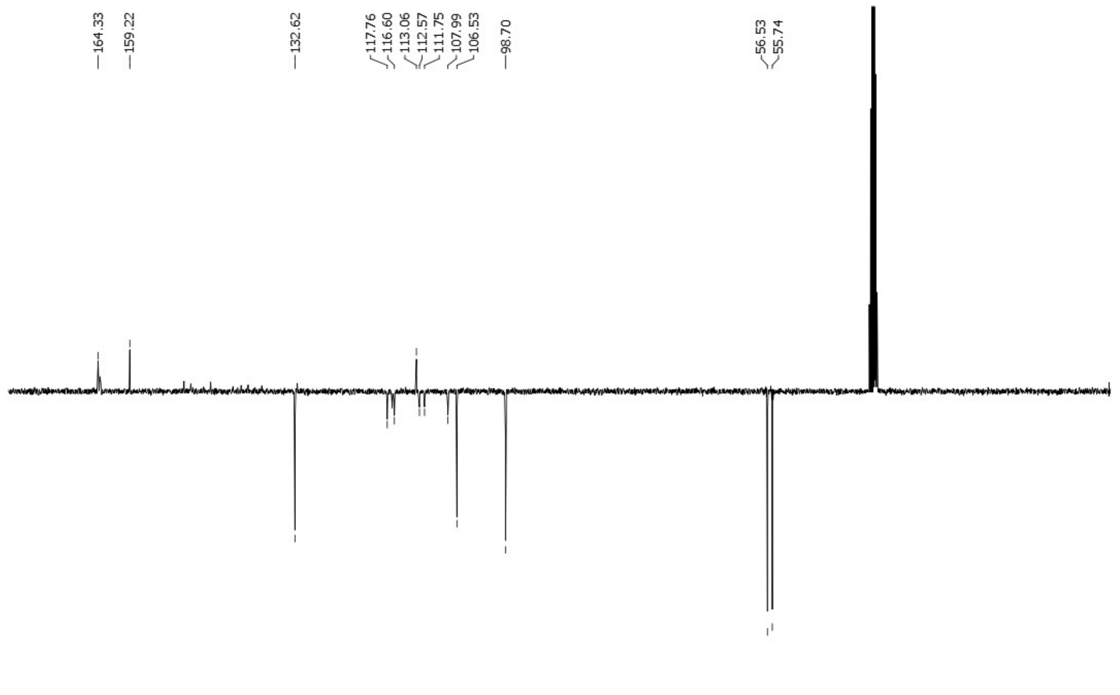

Figure S4. ^{13}C NMR spectrum (DMSO-d_6, 100 MHz) of ***2,4-dimethoxy-N-[5(6)-nitrobenzimidazol-2-yl]benzamide*** *7*.

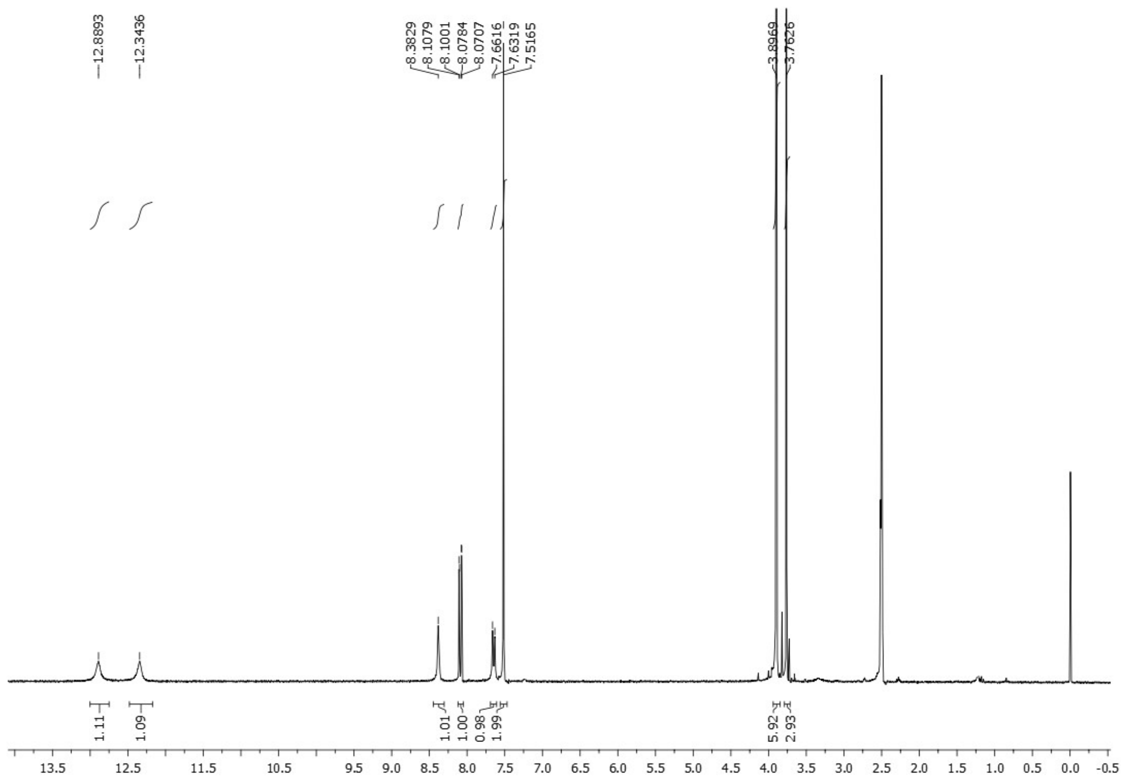

Figure S5. ^{1}H NMR spectrum (DMSO-d_6, 300 MHz) of ***3,4,5-trimethoxy-N-[5(6)-nitrobenzimidazol-2-yl]benzamide 8***.

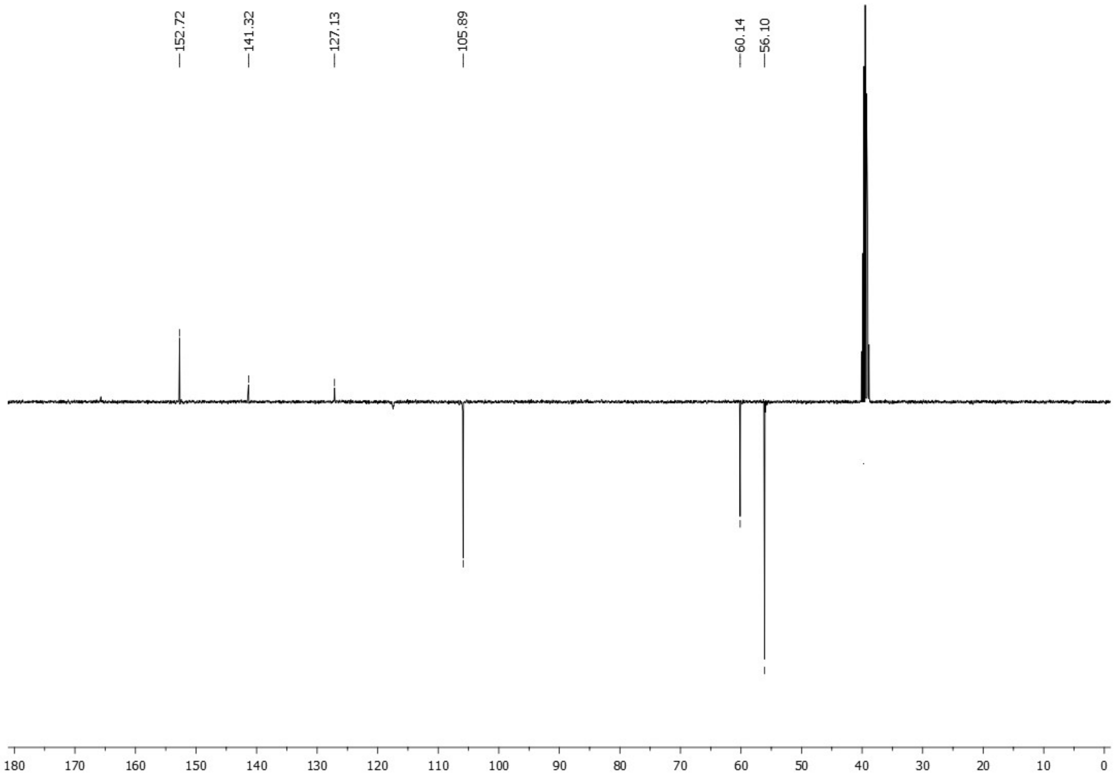

Figure S6. ^{13}C NMR spectrum (DMSO-d_6, 100 MHz) of ***3,4,5-trimethoxy-N-[5(6)-nitrobenzimidazol-2-yl]benzamide 8***.

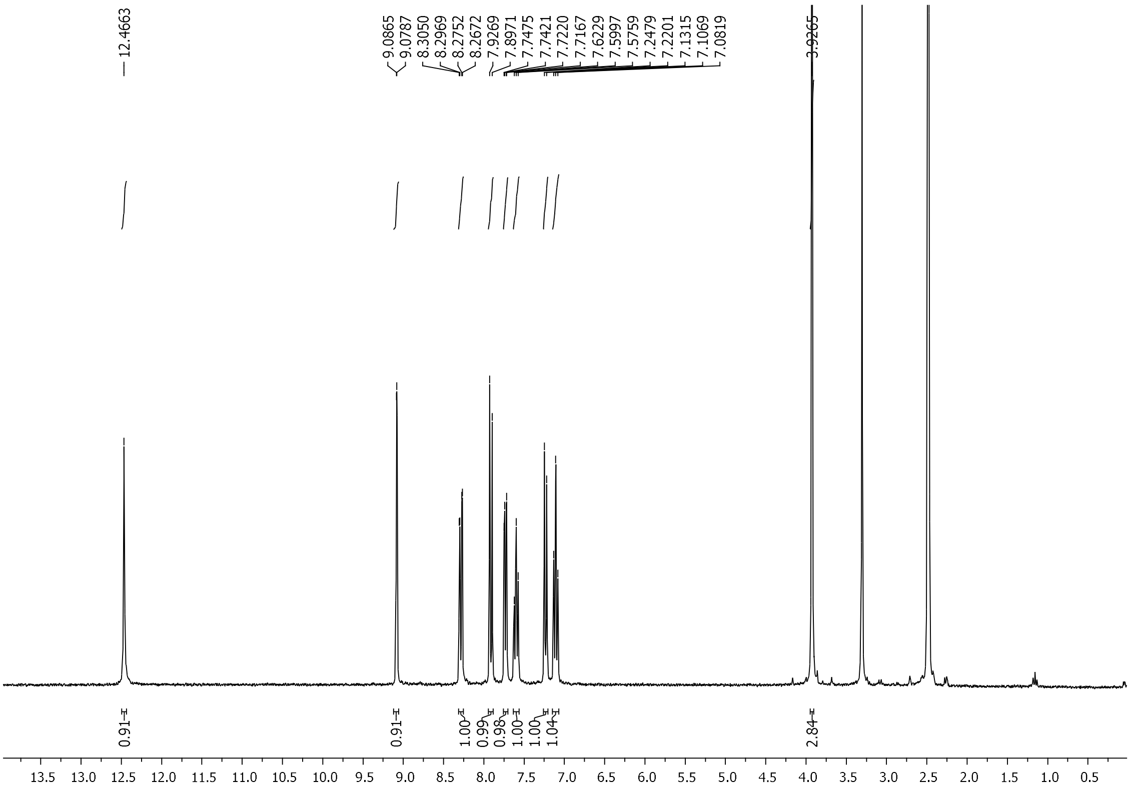

Figure S7. ^{1}H NMR spectrum (DMSO-d_6, 300 MHz) of ***2-methoxy-N-(6-nitrobenzothiazol-2-yl)benzamide 9***.

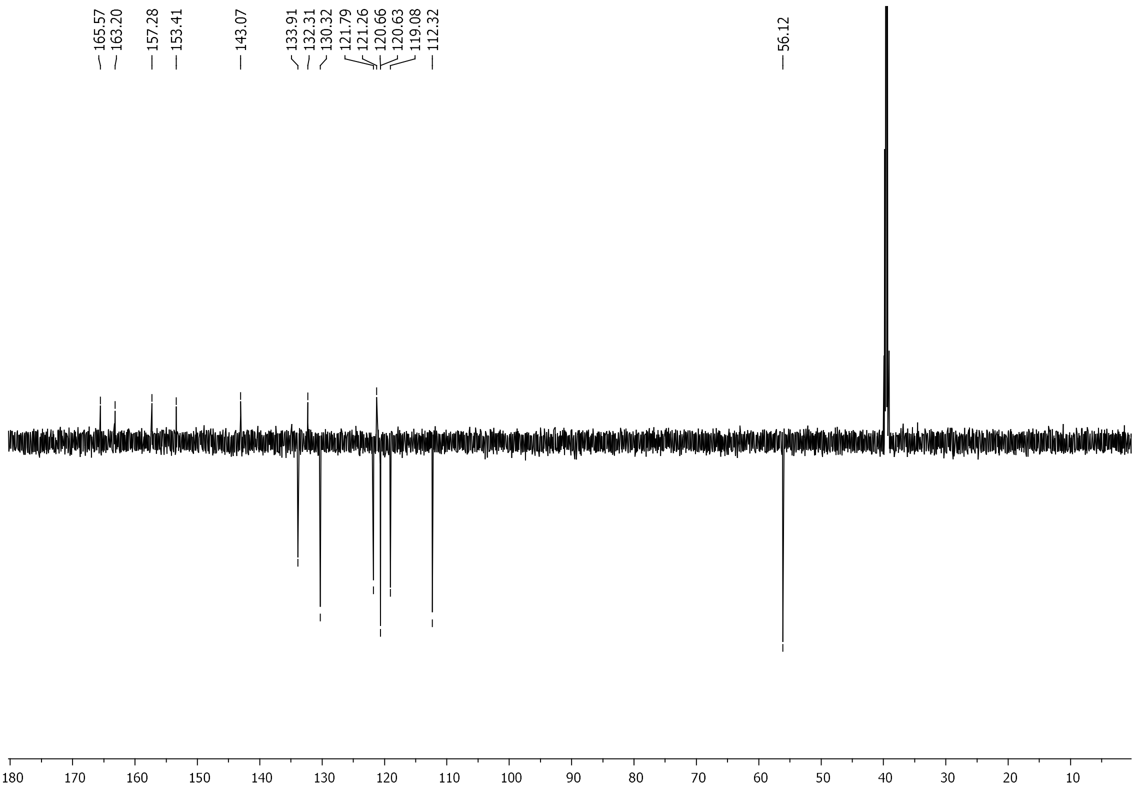

Figure S8. ^{13}C NMR spectrum (DMSO-d_6, 100 MHz) of ***2-methoxy-N-(6-nitrobenzothiazol-2-yl)benzamide 9***.

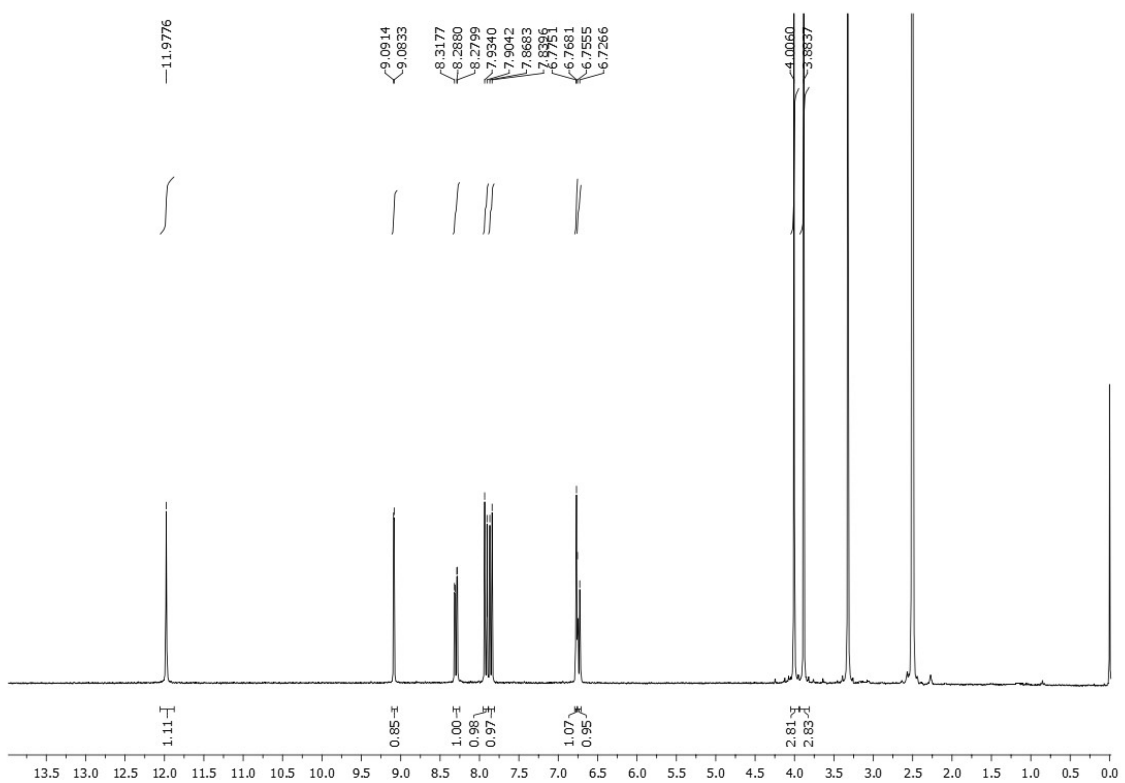

Figure S9. ^{1}H NMR spectrum (DMSO-d_6, 300 MHz) of ***2,4-dimethoxy-N-(6-nitrobenzothiazol-2-yl)benzamide 10***.

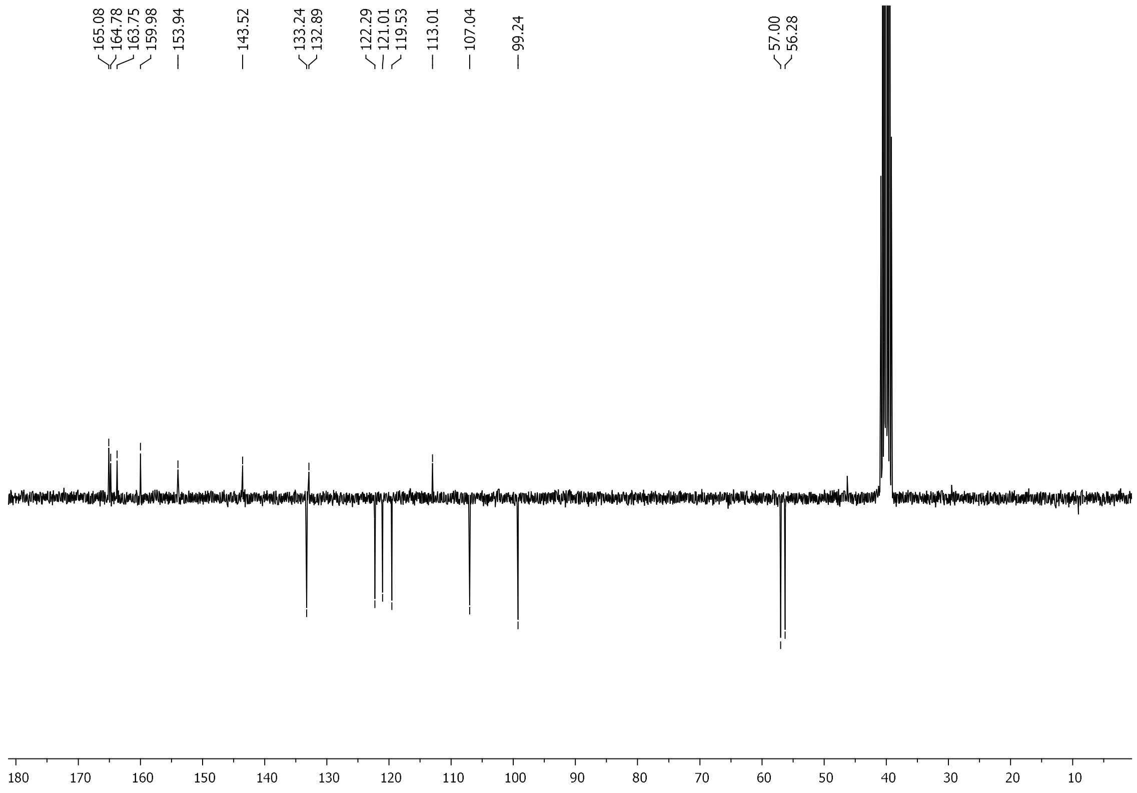

Figure S10. ^{13}C NMR spectrum (DMSO-d_6, 75 MHz) of ***2,4-dimethoxy-N-(6-nitrobenzothiazol-2-yl)benzamide 10***.

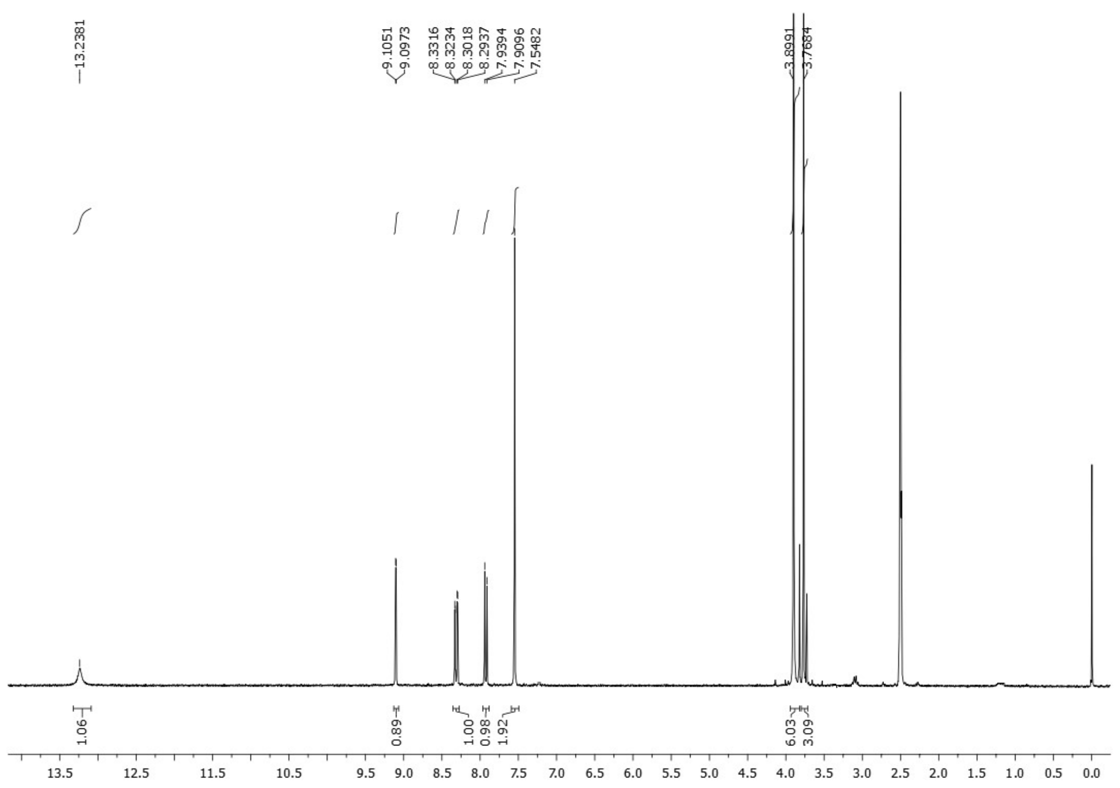

Figure S11. ^{1}H NMR spectrum (DMSO-d_6, 300 MHz) of ***3,4,5-trimethoxy-N-(6-nitrobenzothiazol-2-yl)benzamide 11***.

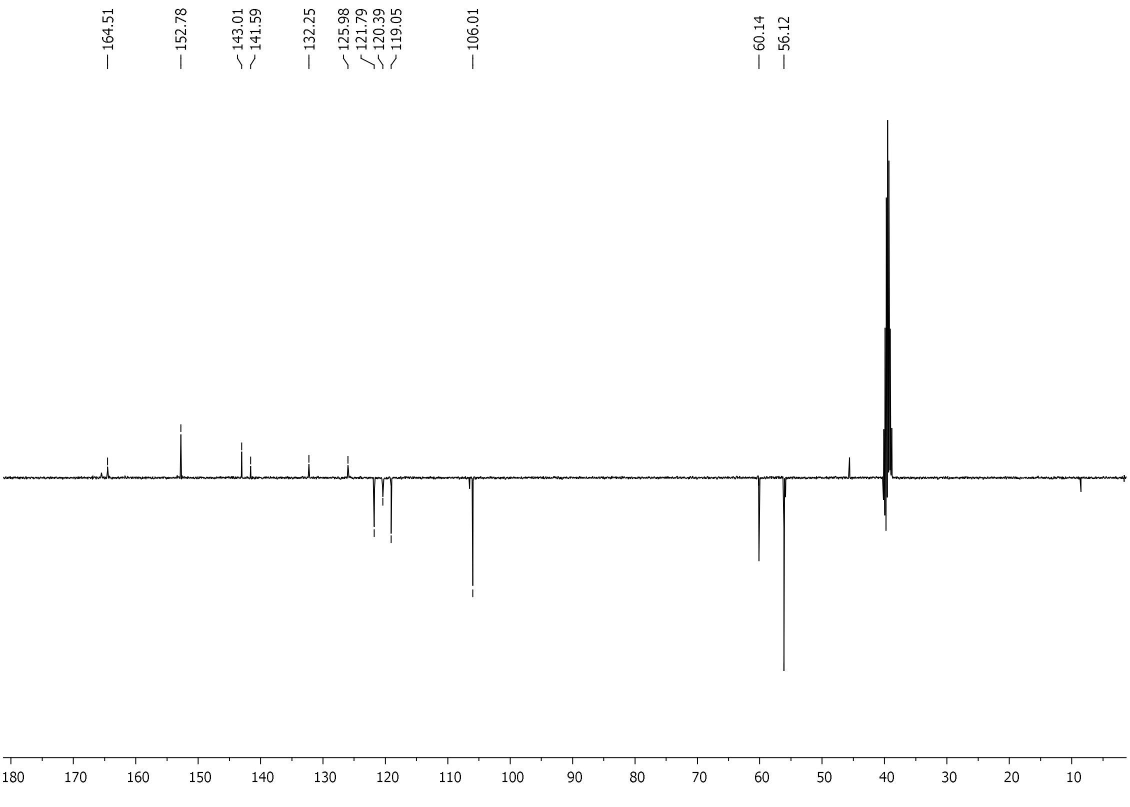

Figure S12. ^{13}C NMR spectrum (DMSO-d_6, 100 MHz) of ***3,4,5-trimethoxy-N-(6-nitrobenzothiazol-2-yl)benzamide 11***.

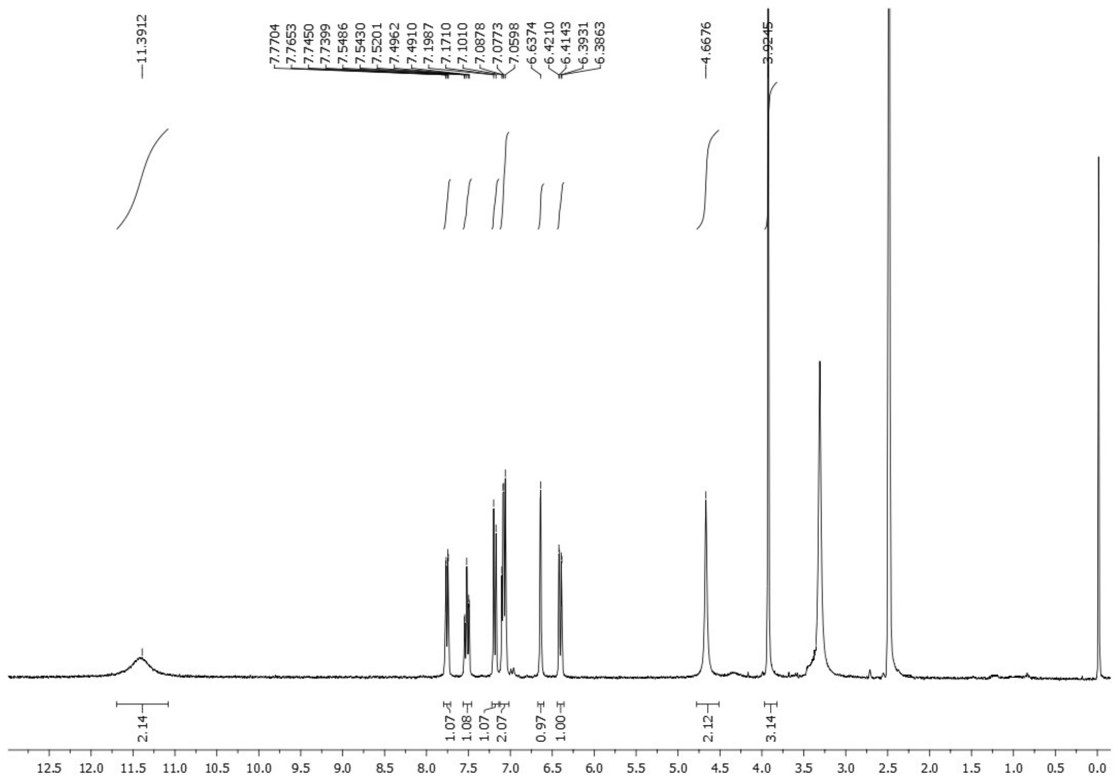

Figure S13. ^{1}H NMR spectrum (DMSO-d_6, 300 MHz) of ***N-[5(6)-aminobenzimidazol-2-yl]-2-methoxybenzamide 12***.

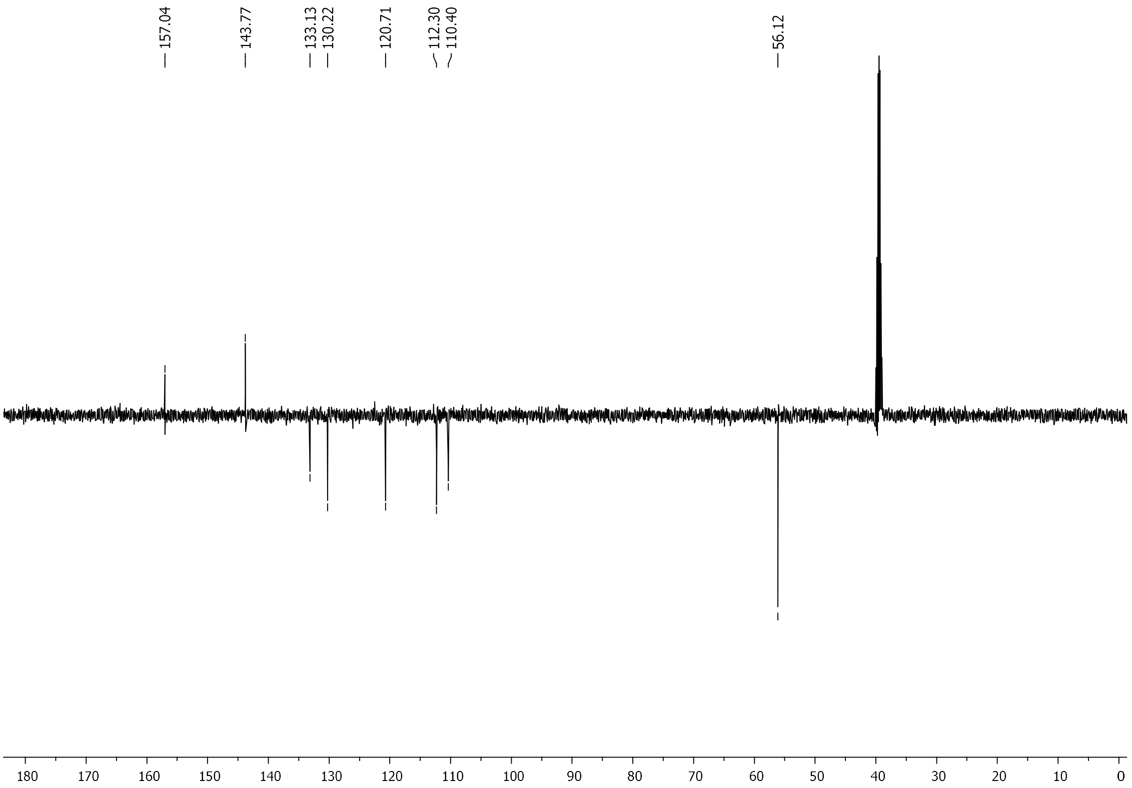

Figure S14. ^{13}C NMR spectrum (DMSO-d_6, 125 MHz) of ***N-[5(6)-aminobenzimidazol-2-yl]-2-methoxybenzamide 12***.

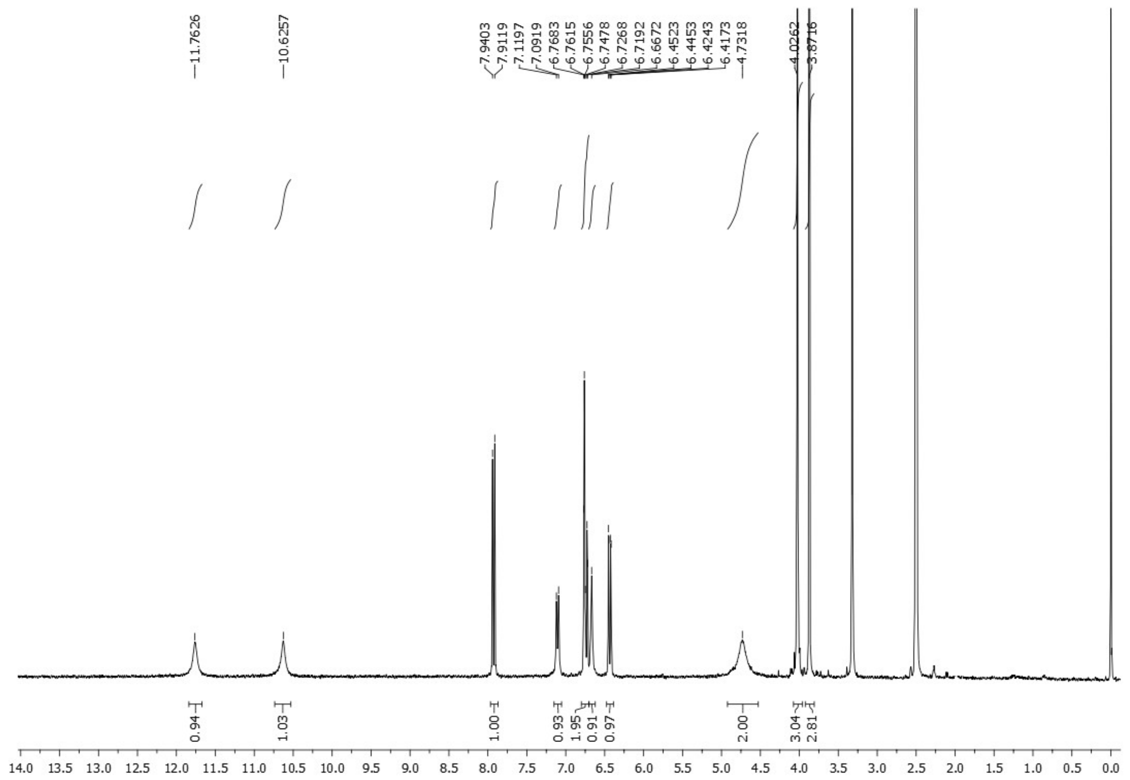

Figure S15. ^{1}H NMR spectrum (DMSO-d_6, 300 MHz) of ***N-[5(6)-aminobenzimidazol-2-yl]-2,4-dimethoxybenzamide 13***.

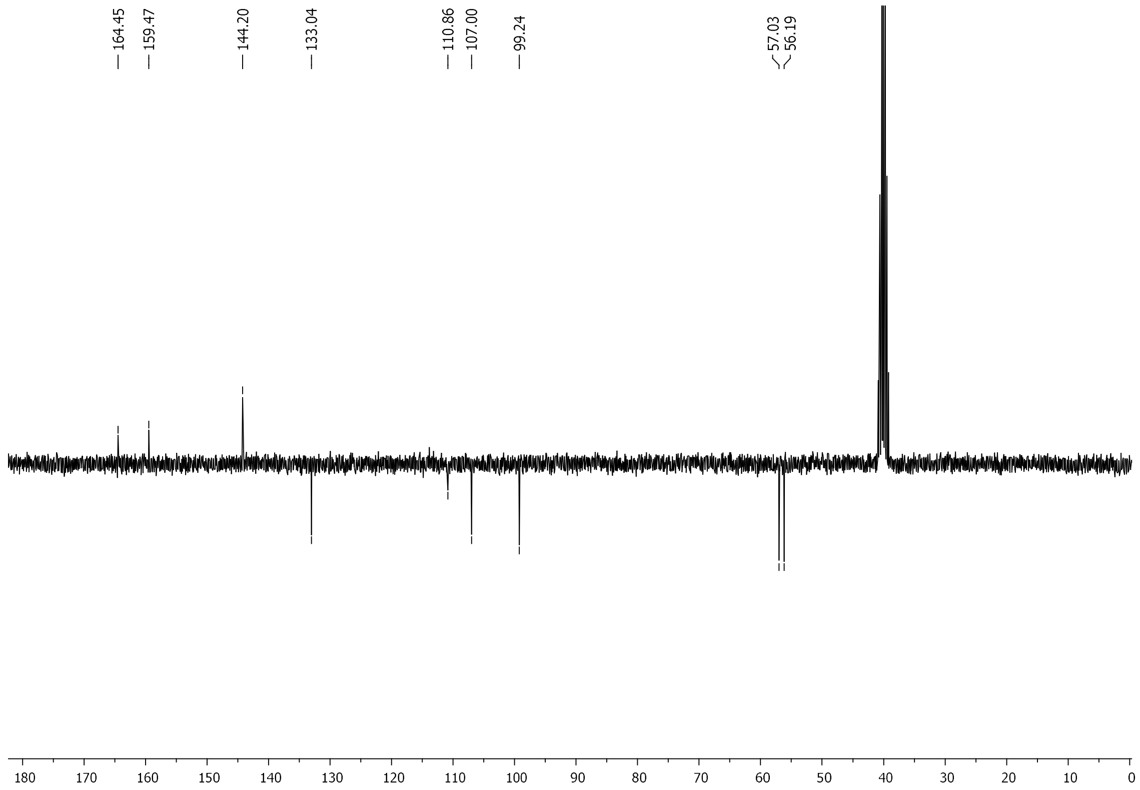

Figure S16. ^{13}C NMR spectrum (DMSO-d_6, 75 MHz) of ***N-[5(6)-aminobenzimidazol-2-yl]-2,4-dimethoxybenzamide 13***.

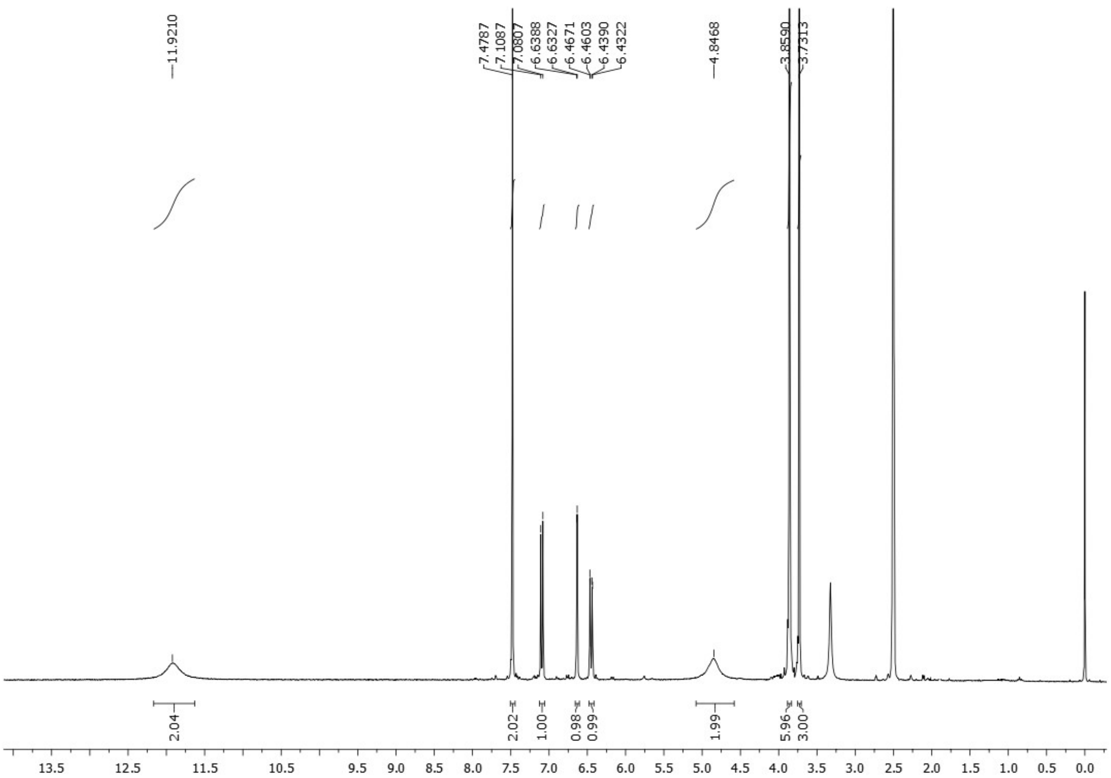

Figure S17. ^{1}H NMR spectrum (DMSO-d_6, 300 MHz) of ***N-[5(6)-aminobenzimidazol-2-yl]-3,4,5-trimethoxybenzamide 14***.

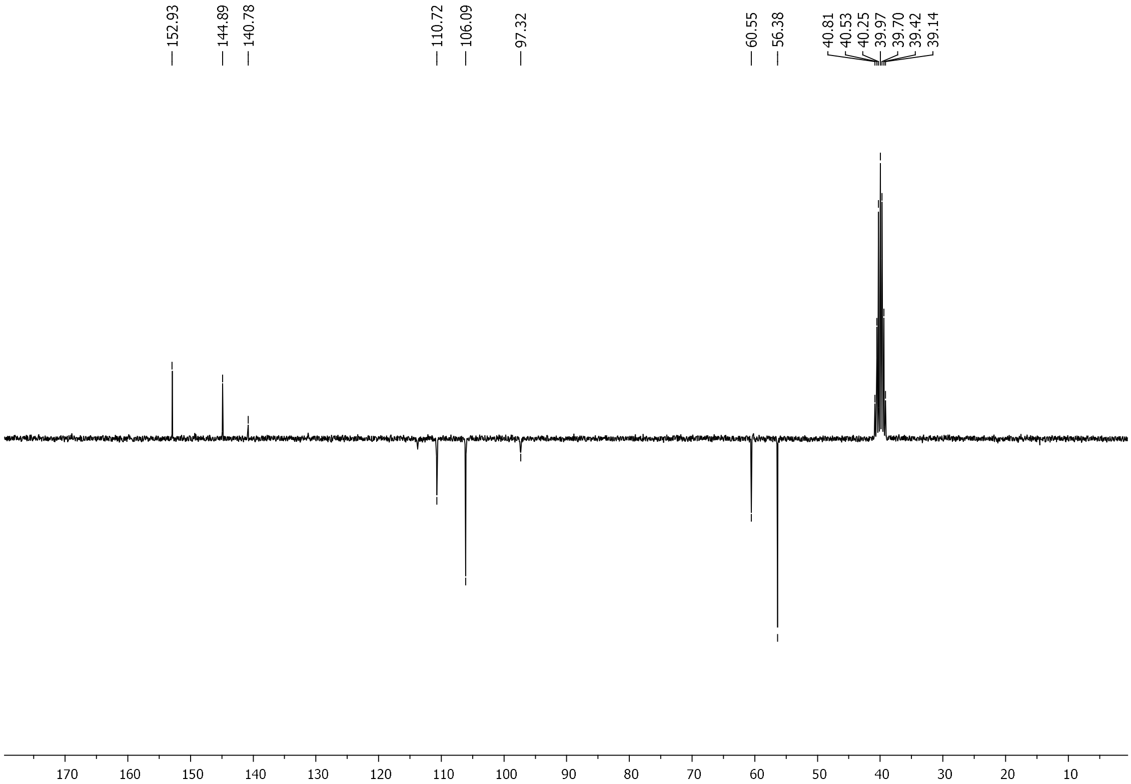

Figure S18. ^{13}C NMR spectrum (DMSO-d_6, 75 MHz) of ***N-[5(6)-aminobenzimidazol-2-yl]-3,4,5-trimethoxybenzamide 14***.

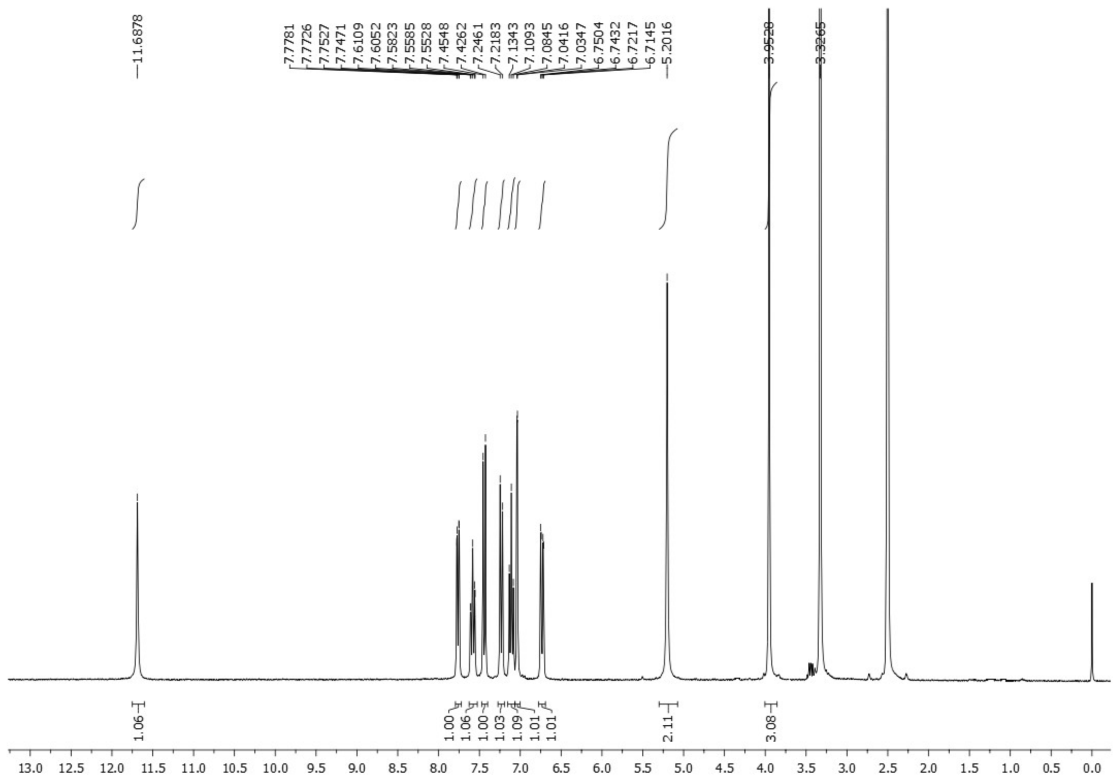

Figure S19. ^{1}H NMR spectrum (DMSO-d_6, 300 MHz) of ***N-(6-aminobenzothiazol-2-yl)-2-methoxybenzamide 15***.

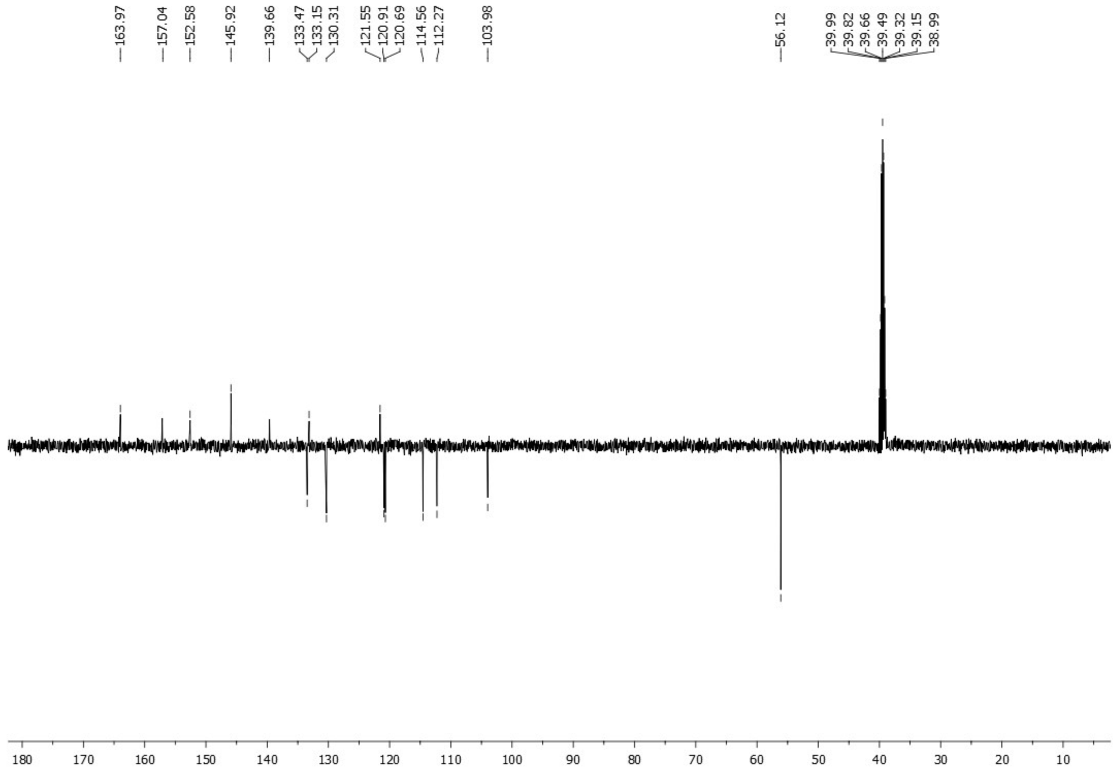

Figure S20. ^{13}C NMR spectrum (DMSO-d_6, 125 MHz) of ***N-(6-aminobenzothiazol-2-yl)-2-methoxybenzamide 15***.

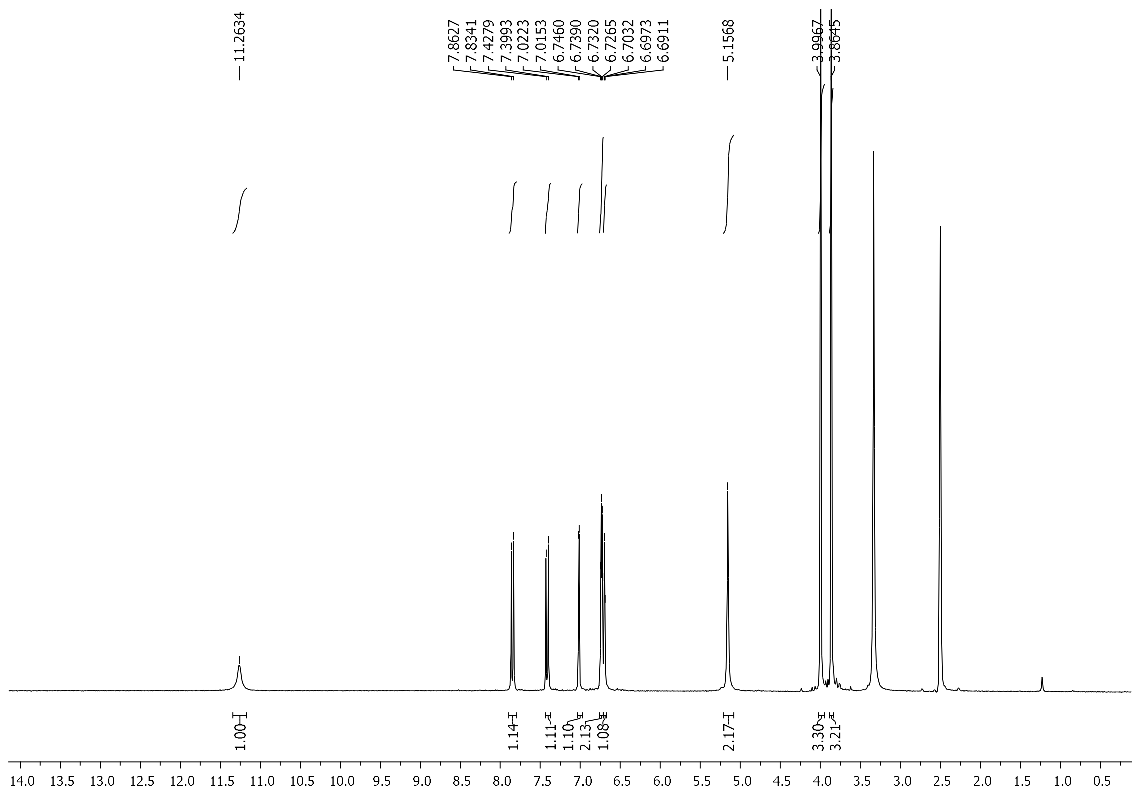

Figure S21. ^{1}H NMR spectrum (DMSO-d_6, 300 MHz) of ***N-(6-aminobenzothiazol-2-yl)-2,4-dimethoxybenzamide 16***.

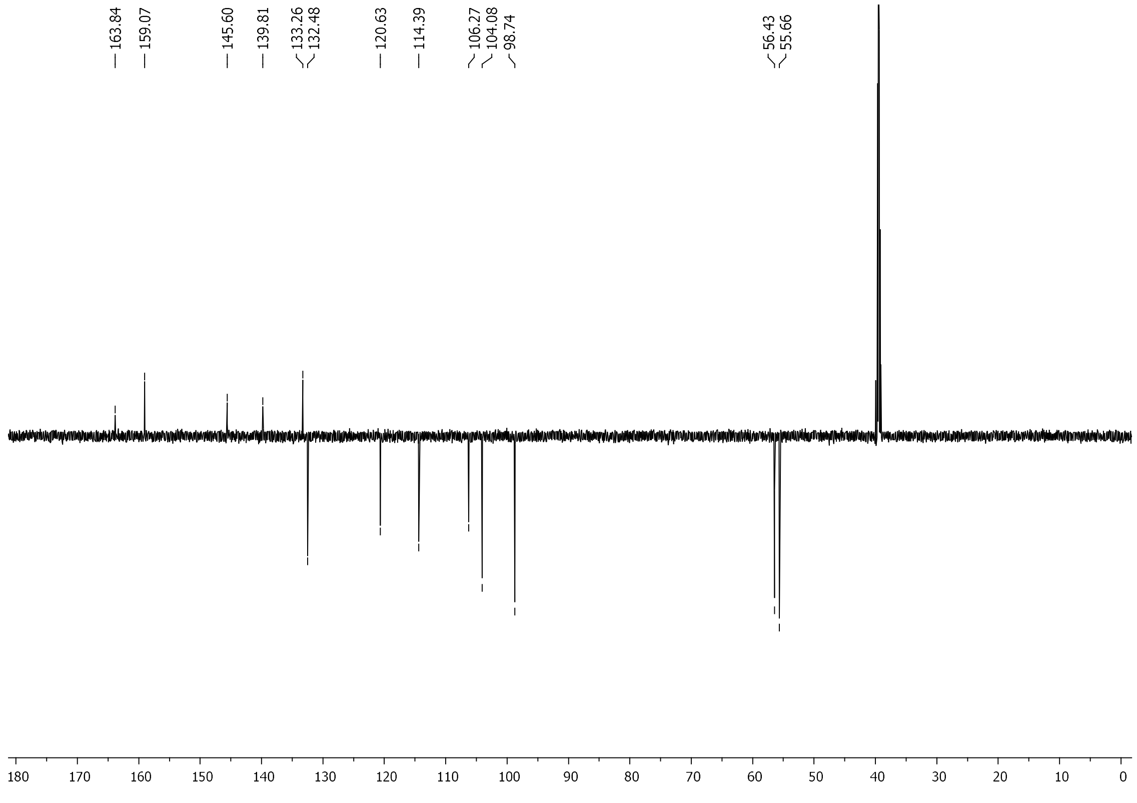

Figure S22. ^{13}C NMR spectrum (DMSO-d_6, 150 MHz) of ***N-(6-aminobenzothiazol-2-yl)-2,4-dimethoxybenzamide 16***.

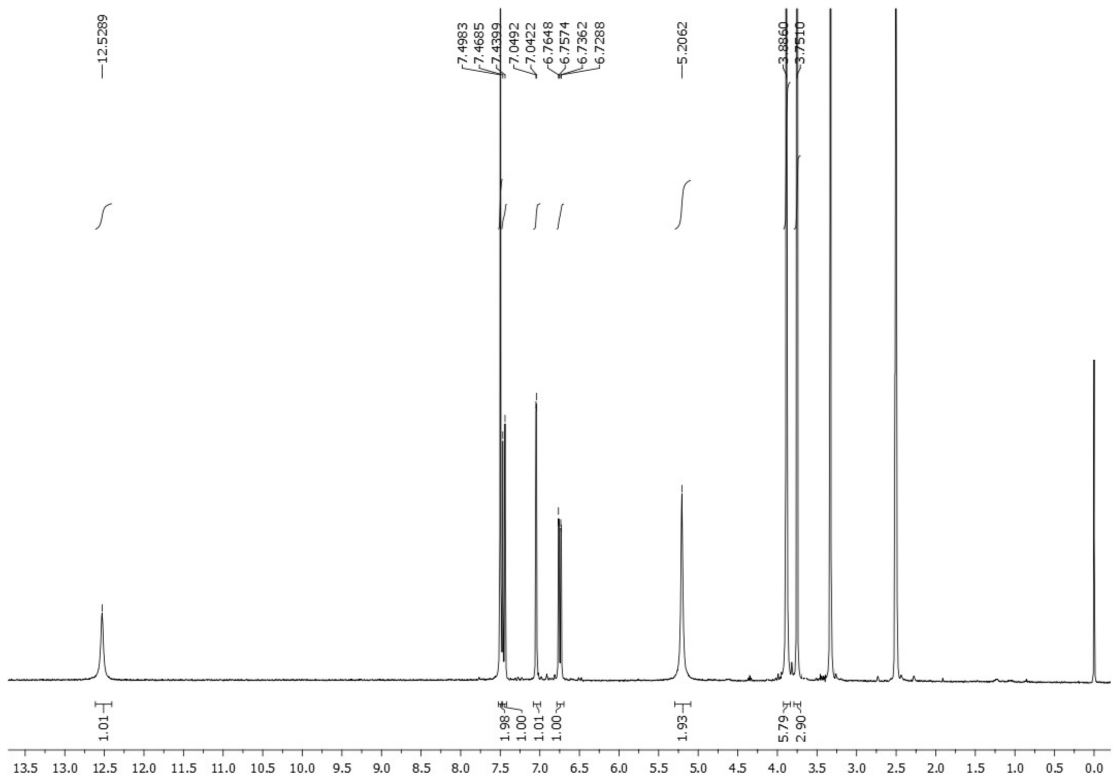

Figure S23. ^{1}H NMR spectrum (DMSO-d_6, 300 MHz) of ***N*-(6-aminobenzothiazol-2-yl)-3,4,5-trimethoxybenzamide 17**.

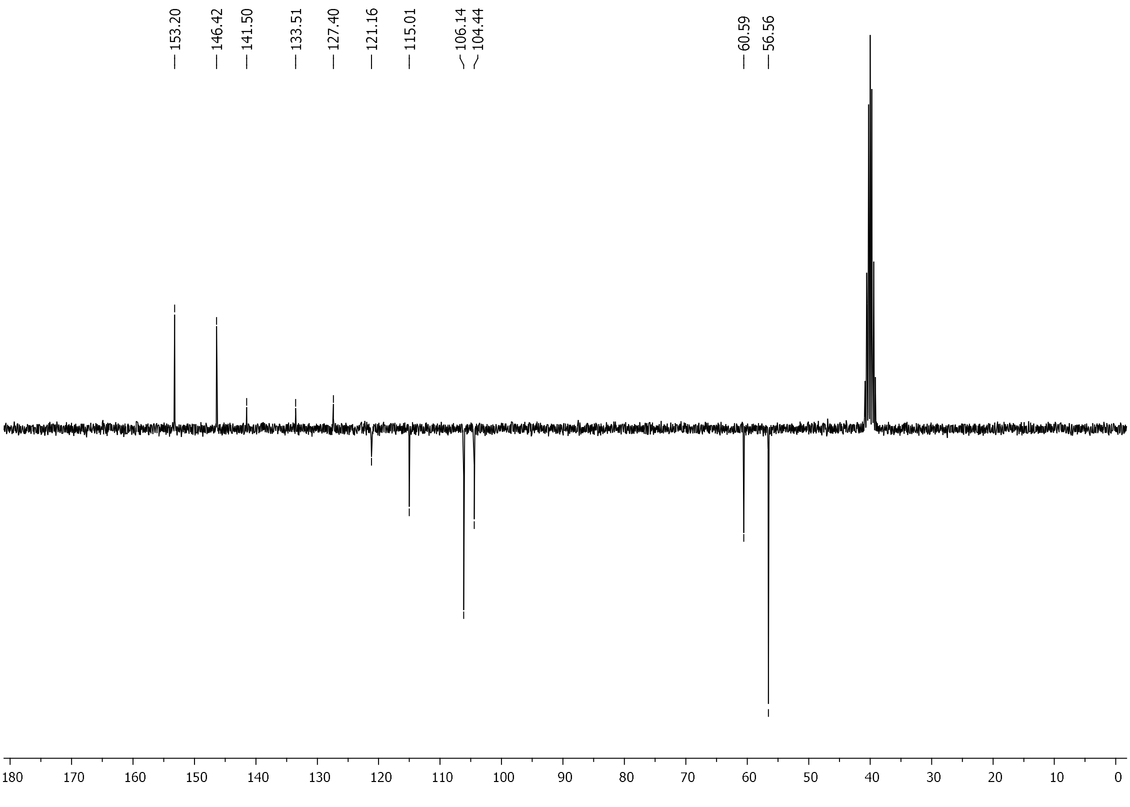

Figure S24. ^{13}C NMR spectrum (DMSO-d_6, 75 MHz) of ***N*-(6-aminobenzothiazol-2-yl)-3,4,5-trimethoxybenzamide 17**.

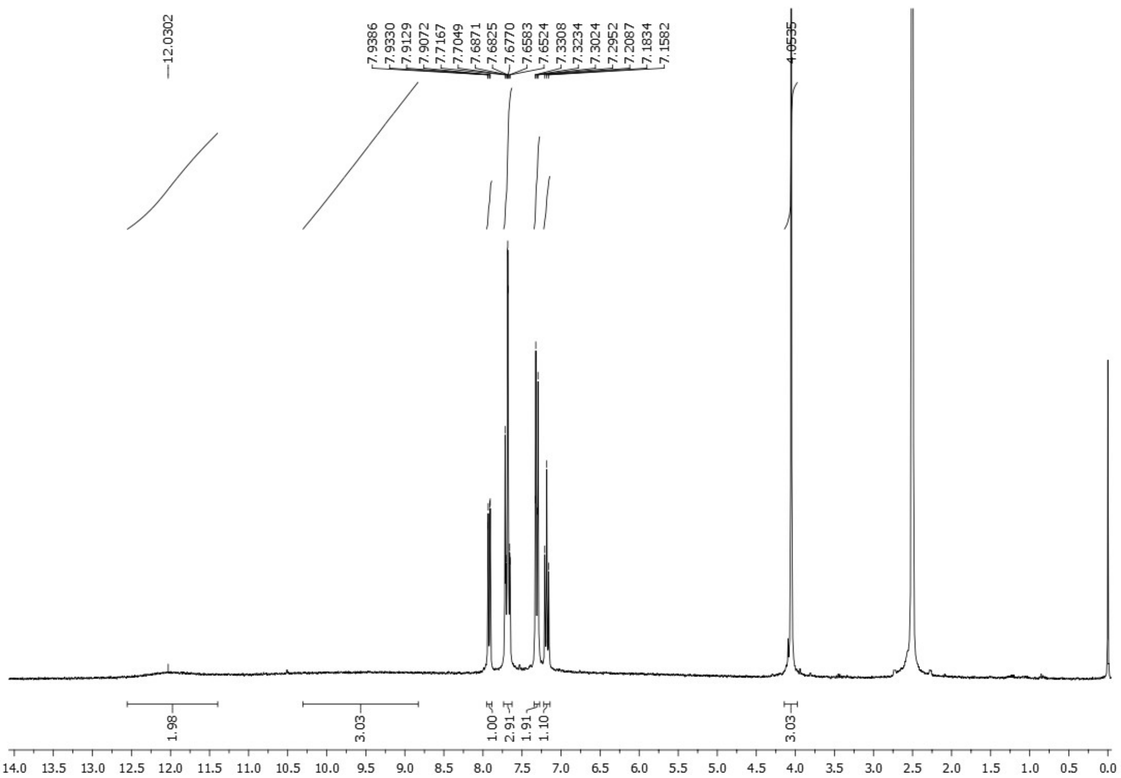

Figure S25. ^{1}H NMR spectrum (DMSO-d_6, 300 MHz) of ***N-[5(6)-aminobenzimidazol-2-yl]-2-methoxybenzamide hydrochloride 18***.

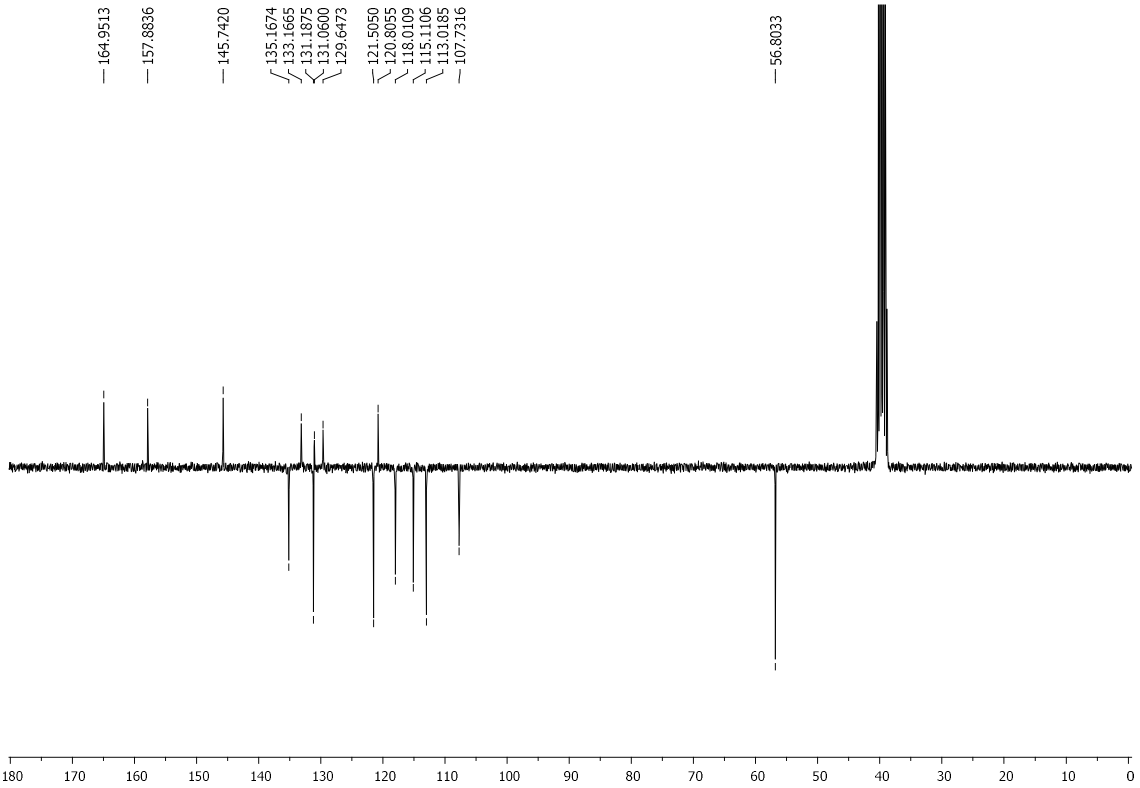

Figure S26. ^{13}C NMR spectrum (DMSO-d_6, 75 MHz) of ***N-[5(6)-aminobenzimidazol-2-yl]-2-methoxybenzamide hydrochloride 18***.

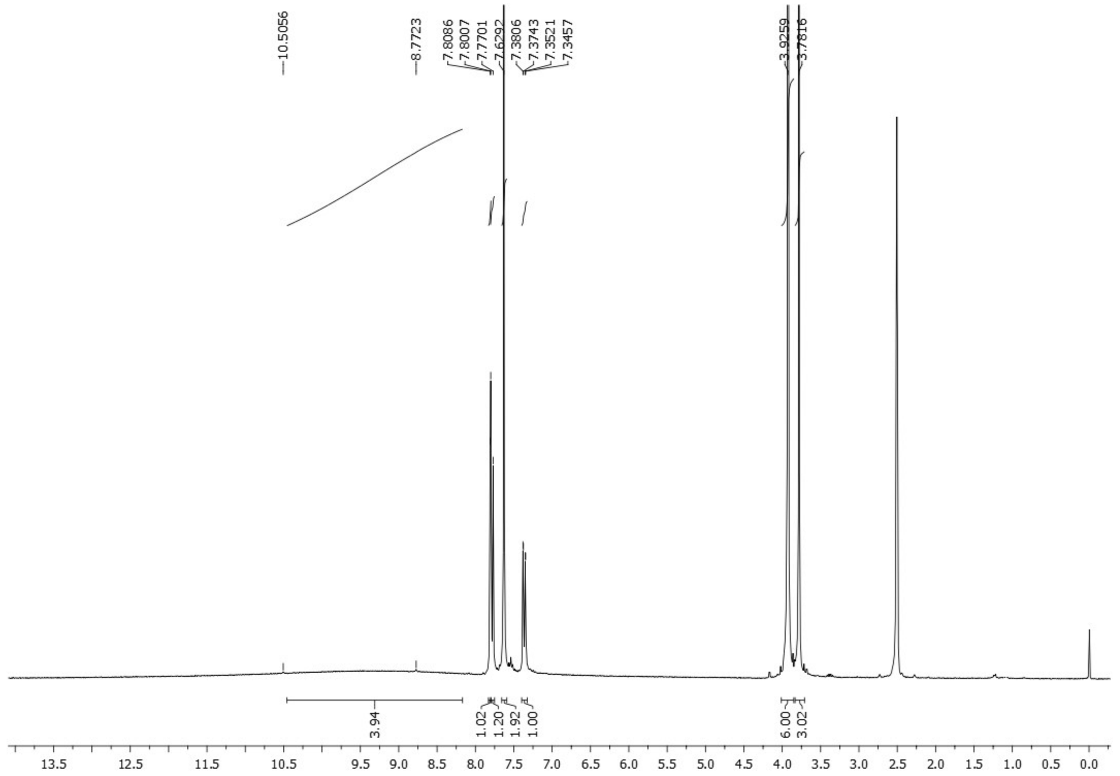

Figure S27. ^{1}H NMR spectrum (DMSO-d_6, 300 MHz) of ***N-(6-aminobenzimidazol-2-yl)-3,4,5-trimethoxybenzamide hydrochloride 20***.

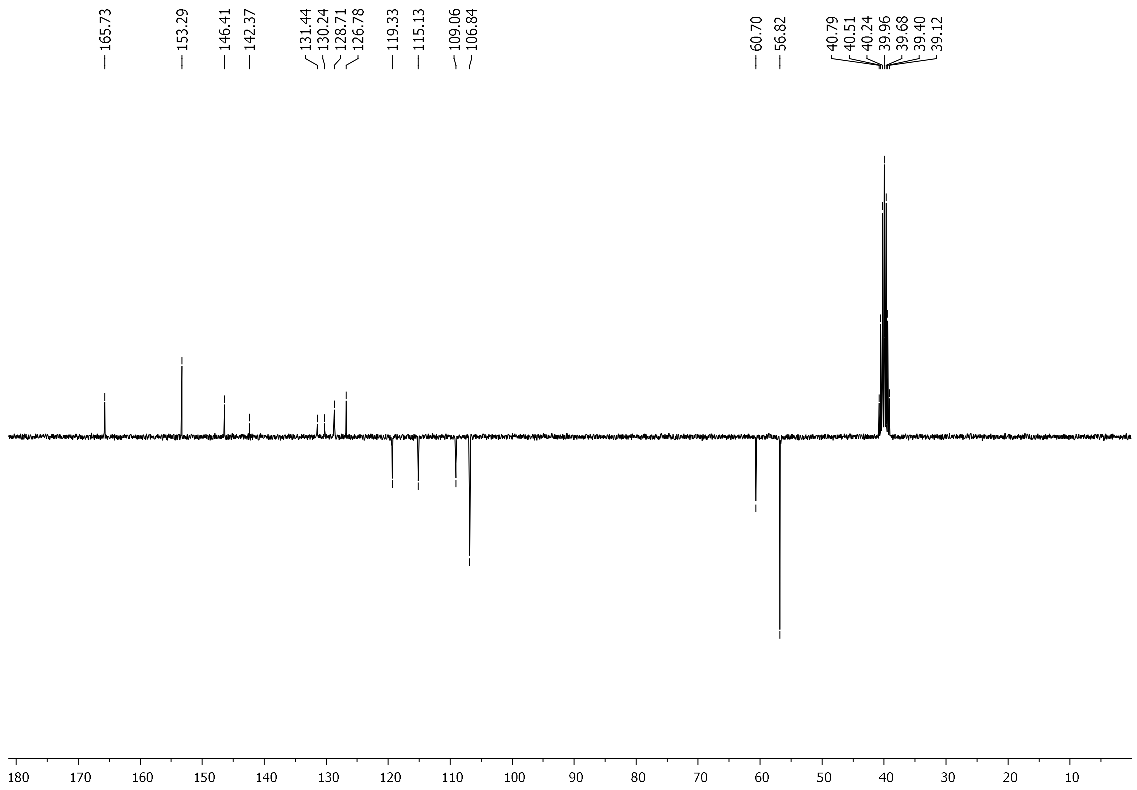

Figure S28. ^{13}C NMR spectrum (DMSO-d_6, 75 MHz) of ***N-(6-aminobenzimidazol-2-yl)-3,4,5-trimethoxybenzamide hydrochloride 20***.

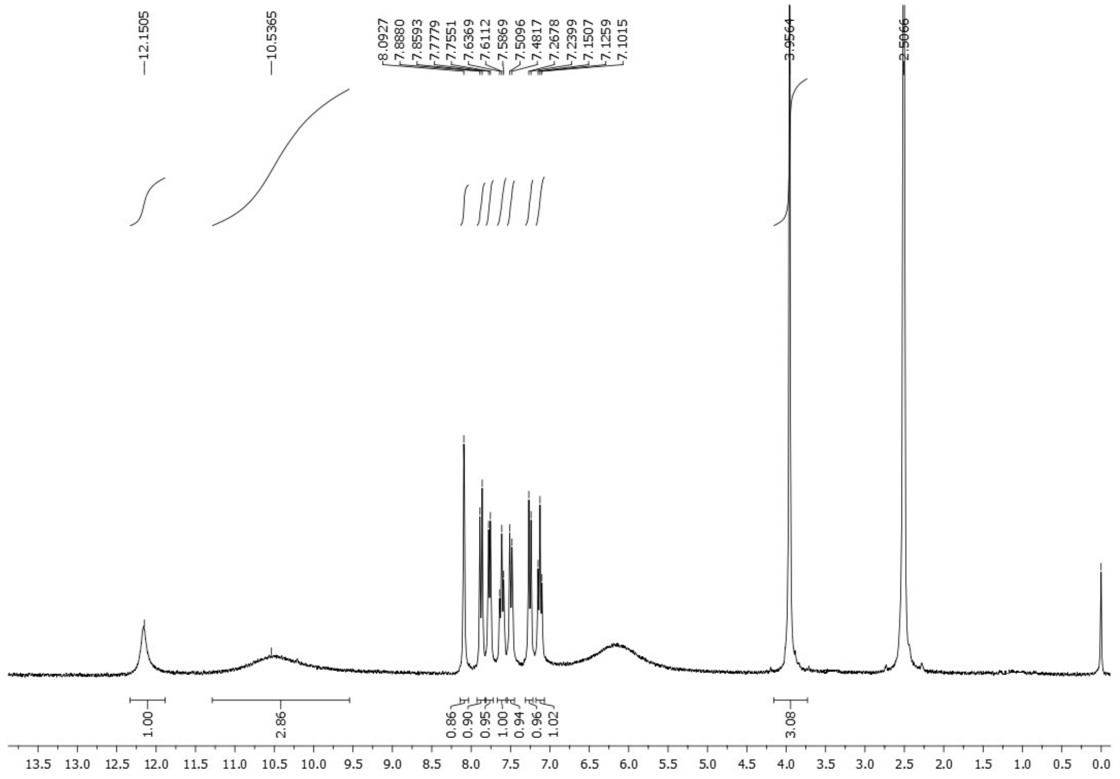

Figure S29. ^{1}H NMR spectrum (DMSO-d_6, 300 MHz) of ***N*-(6-aminobenzothiazol-2-yl)-2-methoxybenzamide hydrochloride 21**.

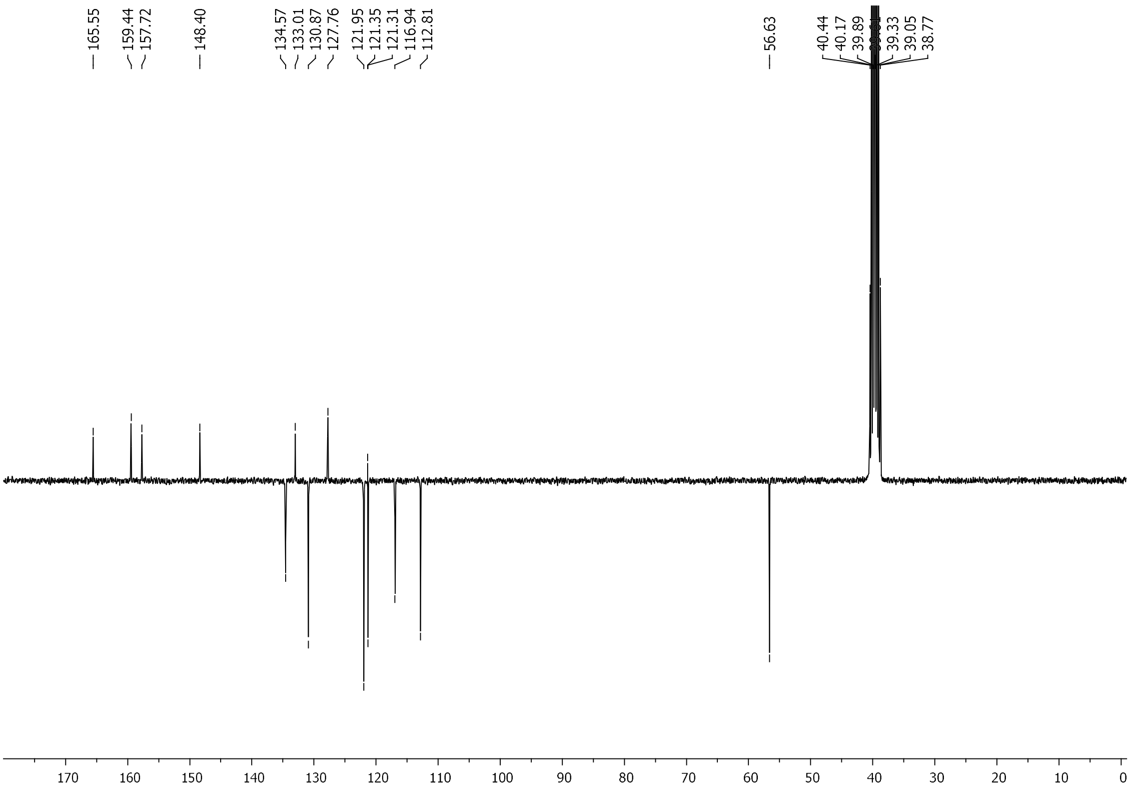

Figure S30. ^{13}C NMR spectrum (DMSO-d_6, 75 MHz) of ***N*-(6-aminobenzothiazol-2-yl)-2-methoxybenzamide hydrochloride 21**.

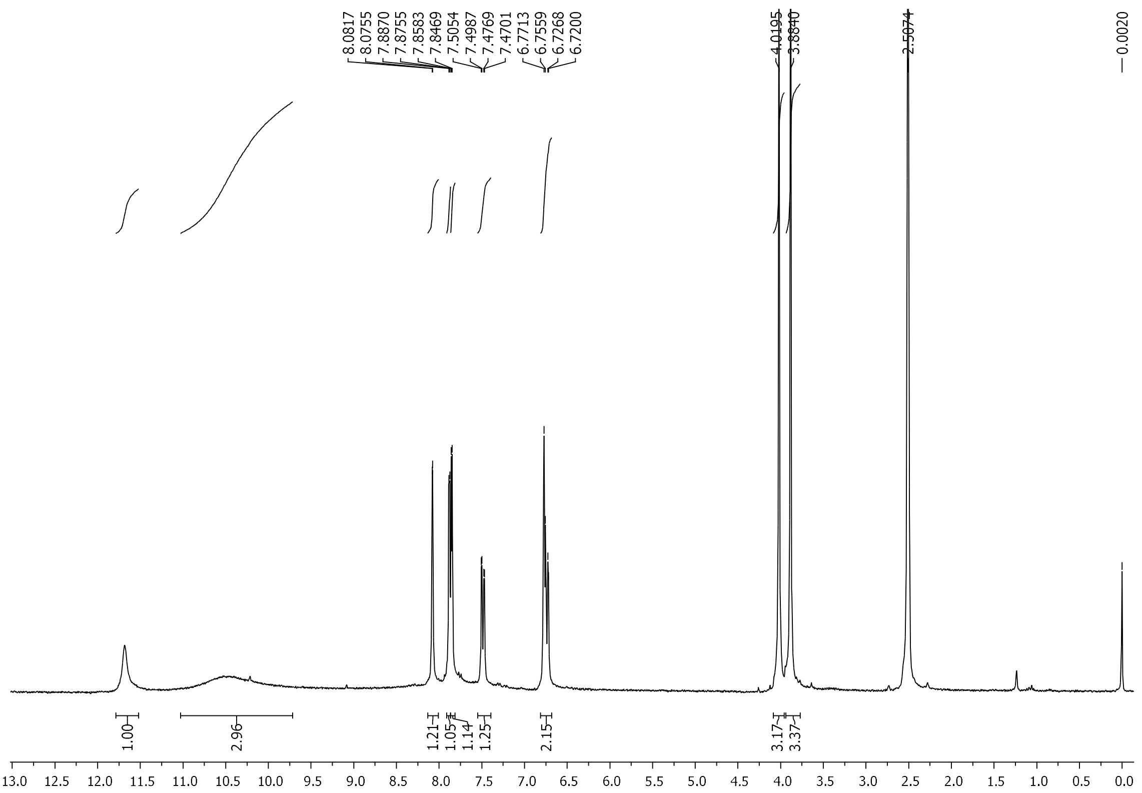

Figure S31. ^{1}H NMR spectrum (DMSO-d_6, 300 MHz) of ***N-(6-aminobenzothiazol-2-yl)-2,4-dimethoxybenzamide hydrochloride 22***.

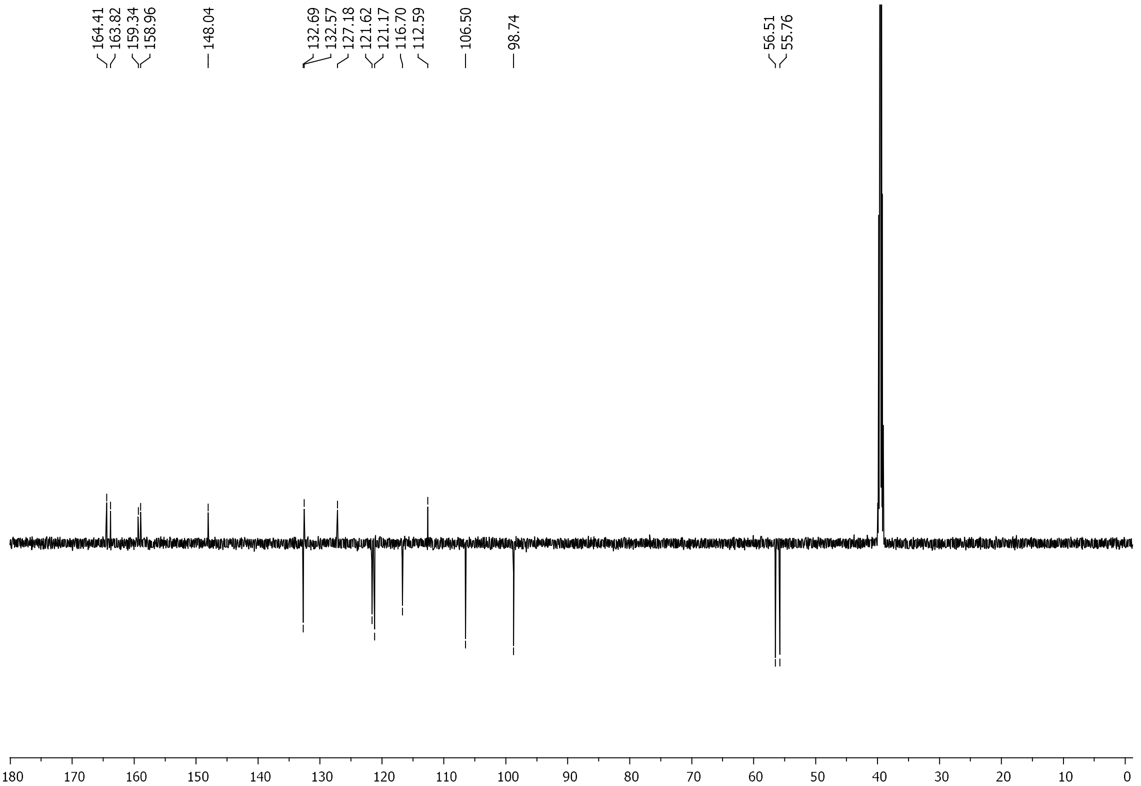

Figure S32. ^{13}C NMR spectrum (DMSO-d_6, 150 MHz) of ***N-(6-aminobenzothiazol-2-yl)-2,4-dimethoxybenzamide hydrochloride 22***.

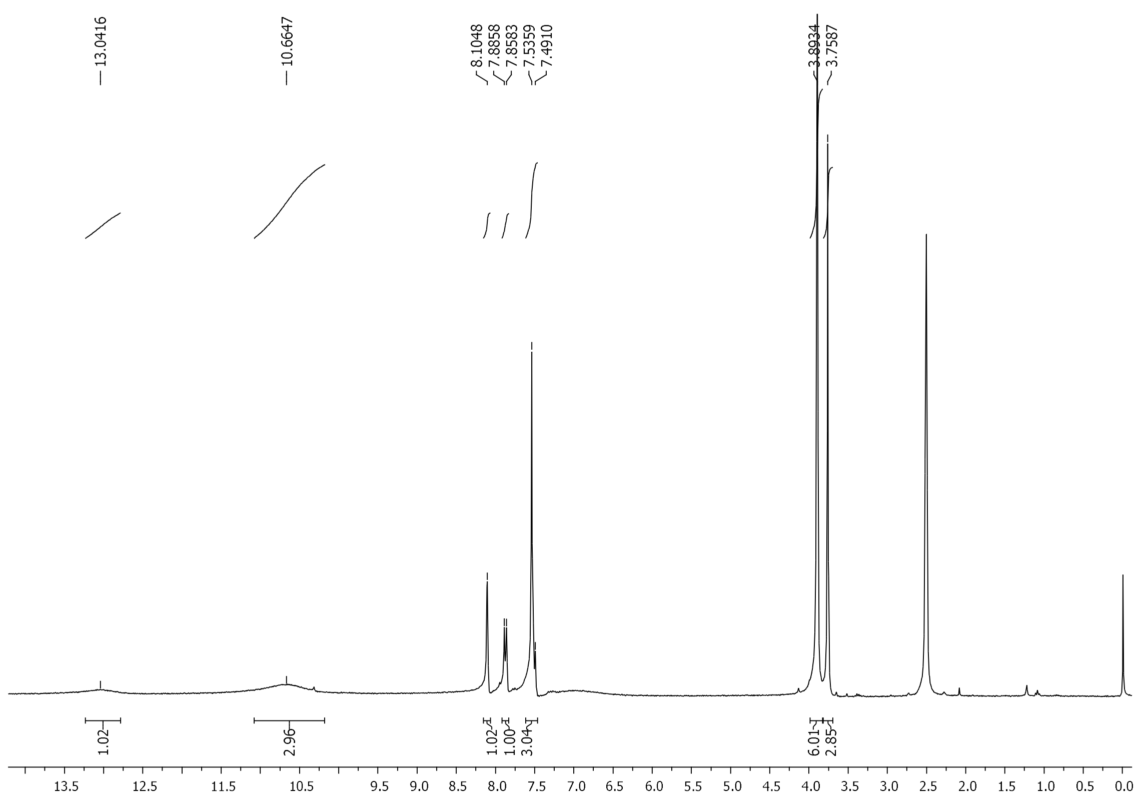

Figure S33. ^{1}H NMR spectrum (DMSO-d_6, 300 MHz) of ***N-(6-aminobenzothiazol-2-yl)-3,4,5-trimethoxybenzamide hydrochloride 23***.

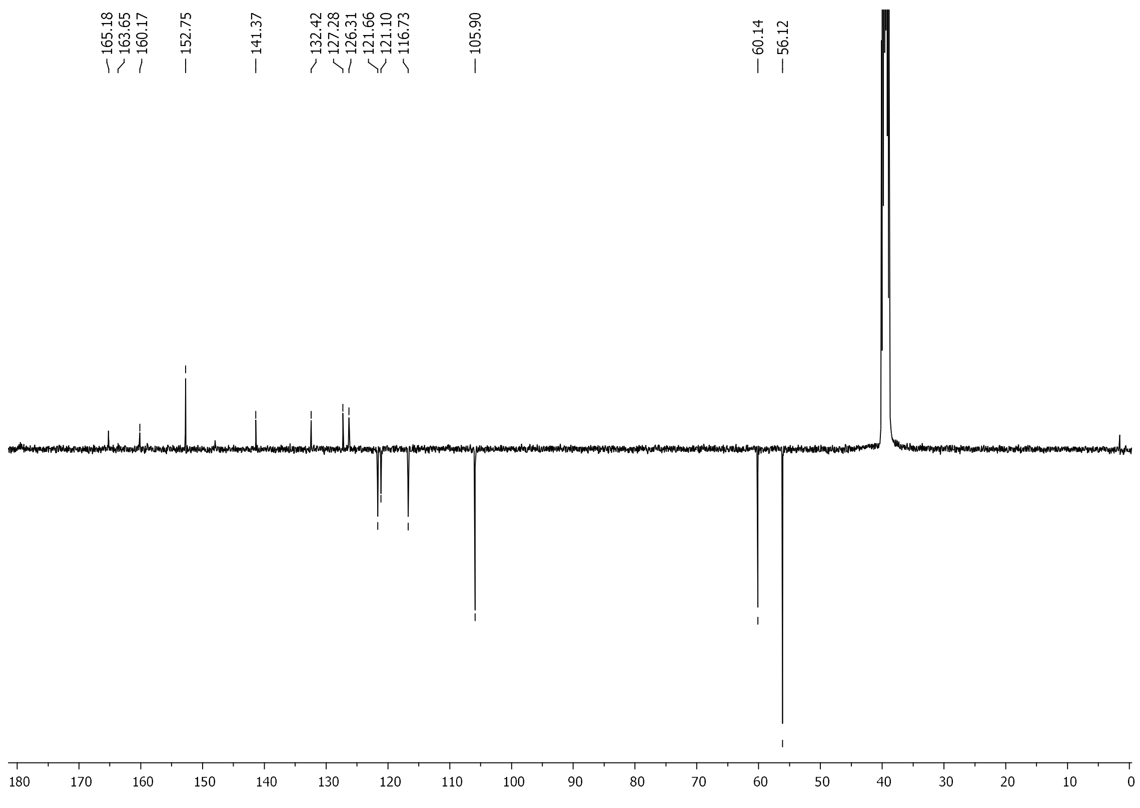

Figure S34. ^{13}C NMR spectrum (DMSO-d_6, 100 MHz) of ***N-(6-aminobenzothiazol-2-yl)-3,4,5-trimethoxybenzamide hydrochloride 23***.

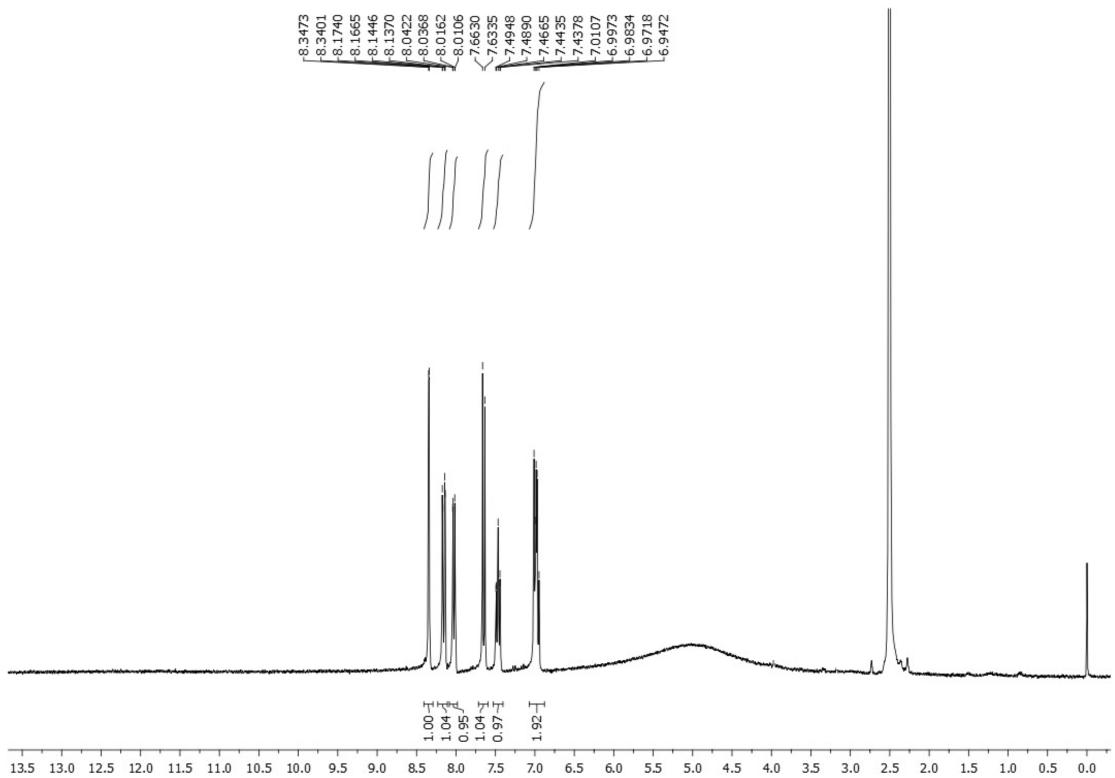

Figure S35. ^{1}H NMR spectrum (DMSO-d_6, 300 MHz) of ***2-hydroxy-N-[5(6)-nitrobenzimidazol-2-yl]benzamide 24***.

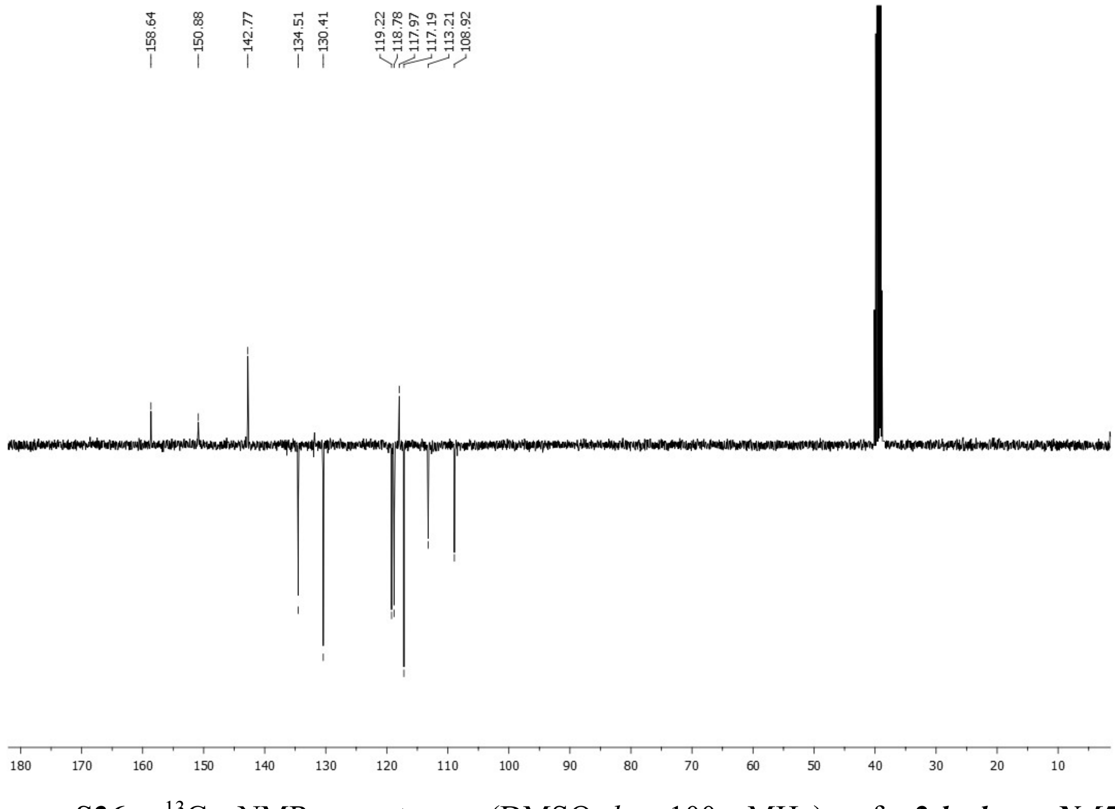

Figure S36. ^{13}C NMR spectrum (DMSO-d_6, 100 MHz) of ***2-hydroxy-N-[5(6)-nitrobenzimidazol-2-yl]benzamide 24***.

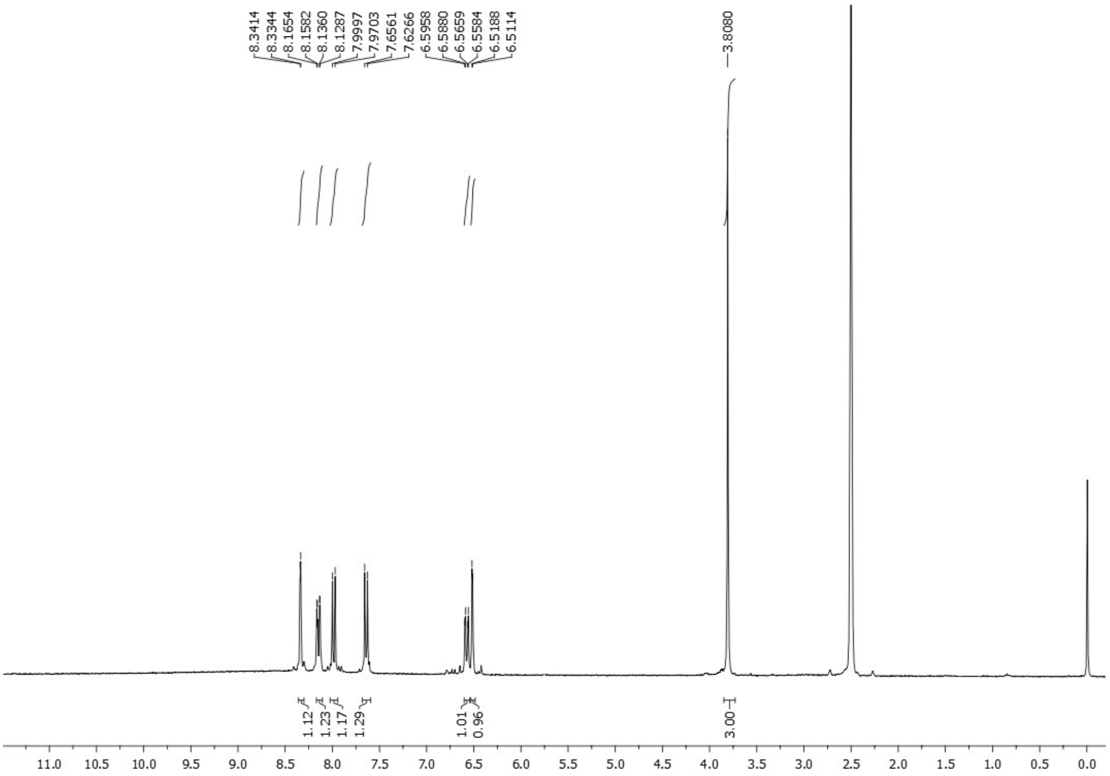

Figure S37. ^{1}H NMR spectrum (DMSO-d_6, 300 MHz) of ***2-hydroxy-4-methoxy-N-[5(6)-nitrobenzimidazol-2-yl]benzamide 25***.

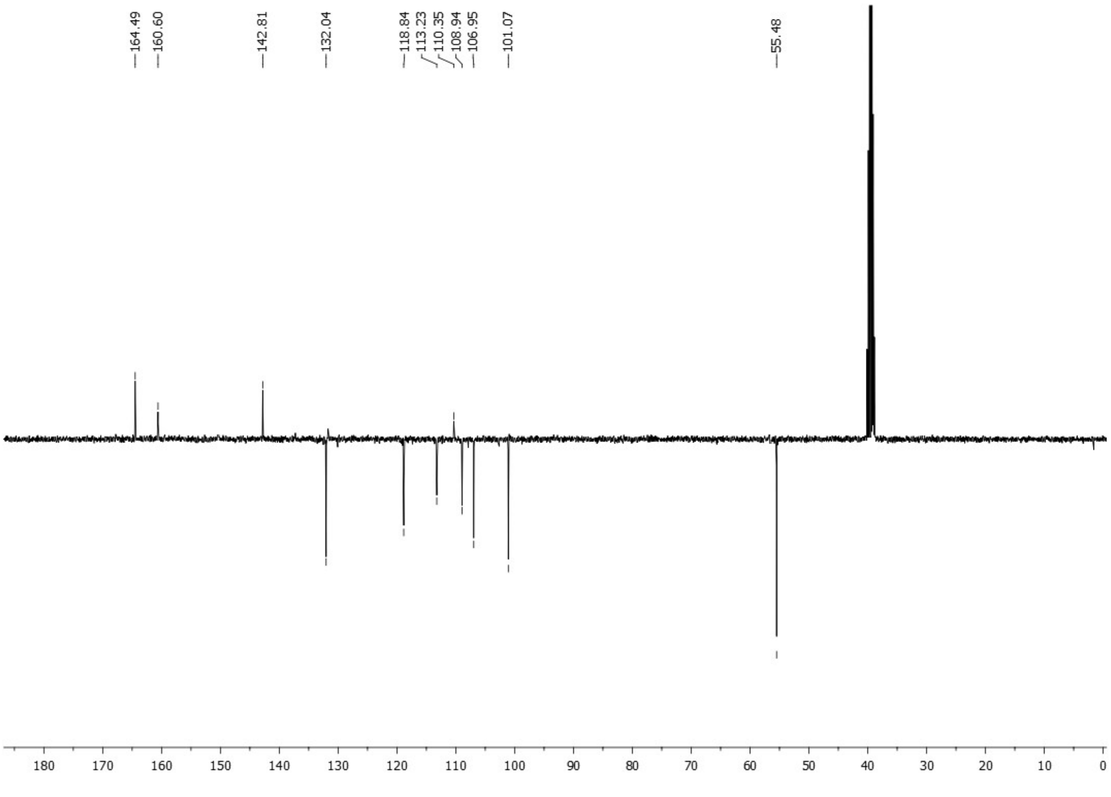

Figure S38. ^{13}C NMR spectrum (DMSO-d_6, 100 MHz) of ***2-hydroxy-4-methoxy-N-[5(6)-nitrobenzimidazol-2-yl]benzamide 25***.

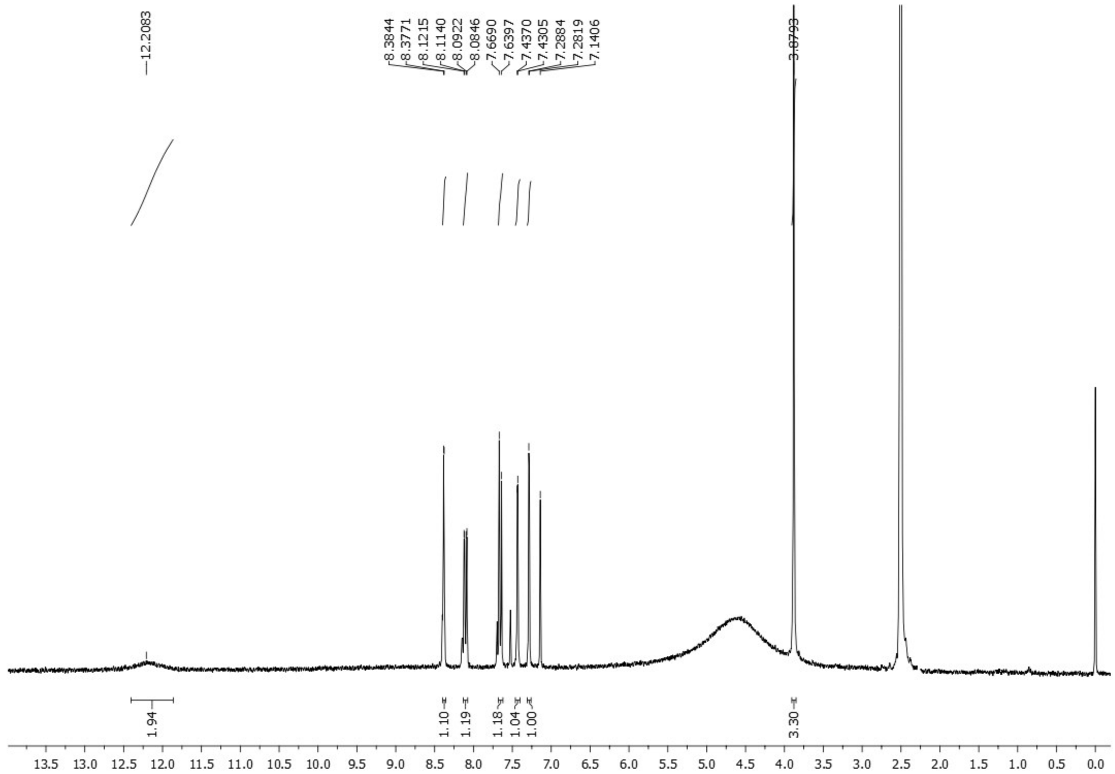

Figure S39. ^{1}H NMR spectrum (DMSO-d_6, 300 MHz) of ***3,5-dihydroxy-4-methoxy-N-[5(6)-nitrobenzimidazol-2-yl]benzamide 26***.

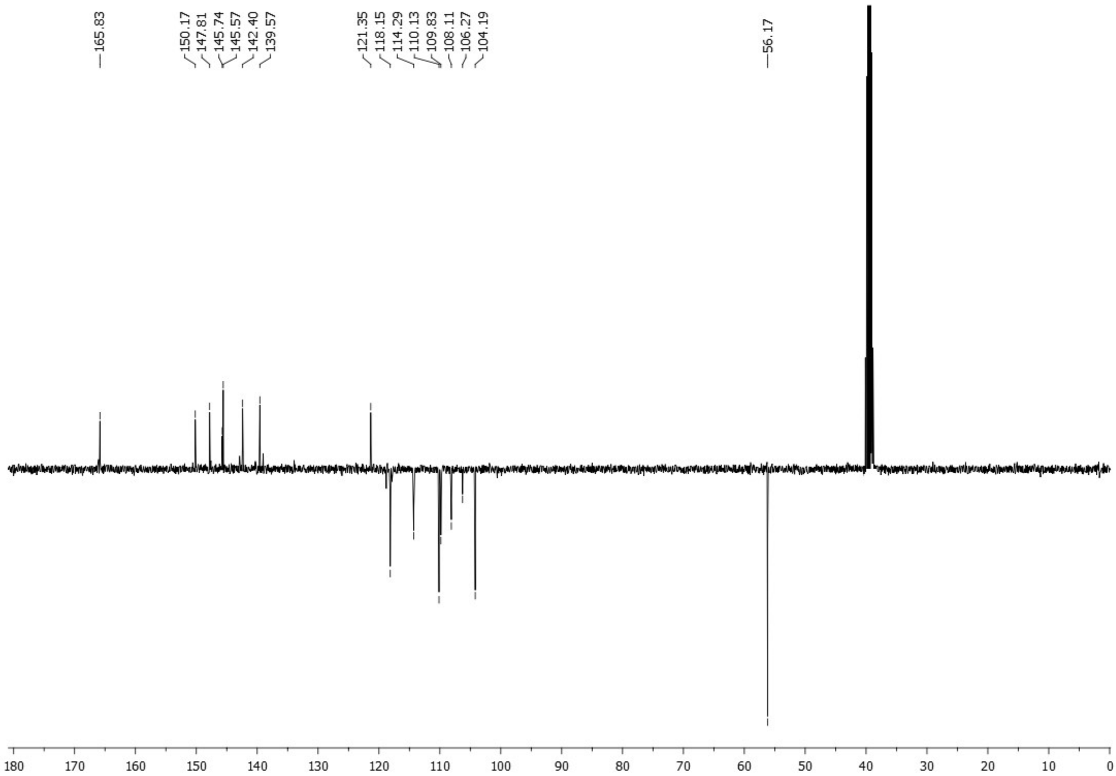

Figure S40. ^{13}C NMR spectrum (DMSO-d_6, 100 MHz) of ***3,5-dihydroxy-4-methoxy-N-[5(6)-nitrobenzimidazol-2-yl]benzamide 26***.

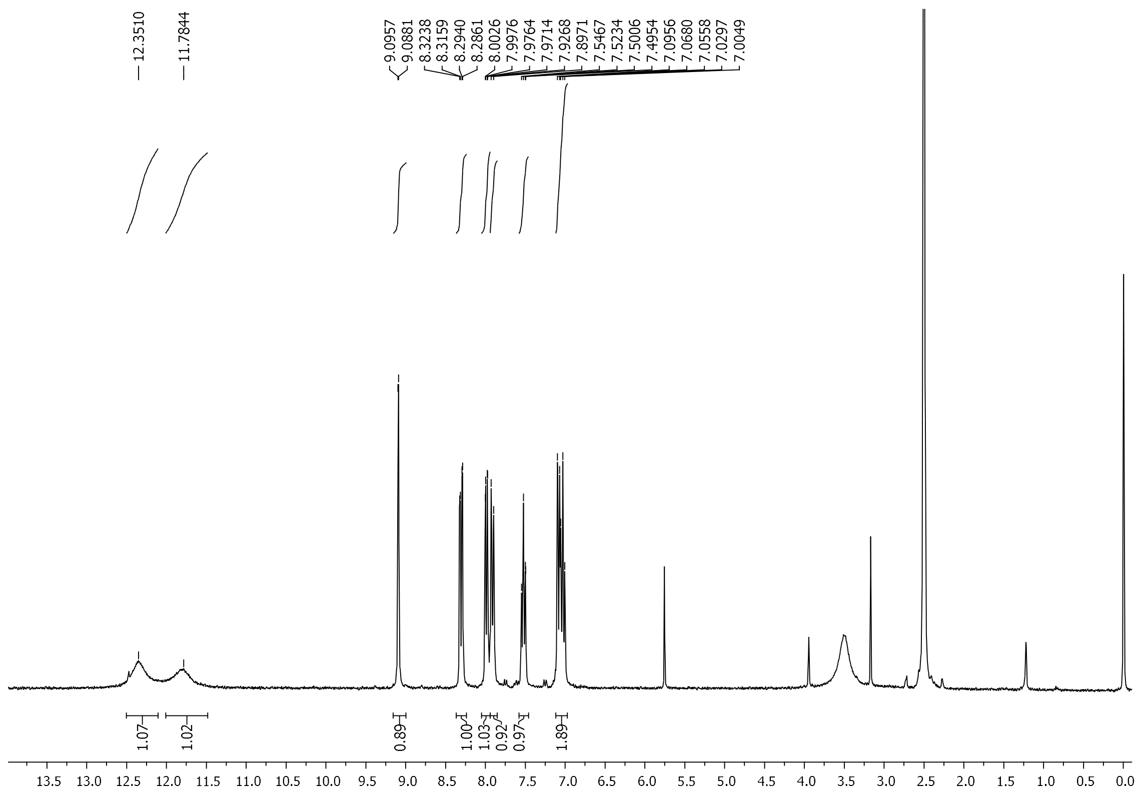

Figure S41. ^{1}H NMR spectrum (DMSO-d_6, 300 MHz) of ***2-hydroxy-N-(6-nitrobenzothiazol-2-yl)benzamide 27***.

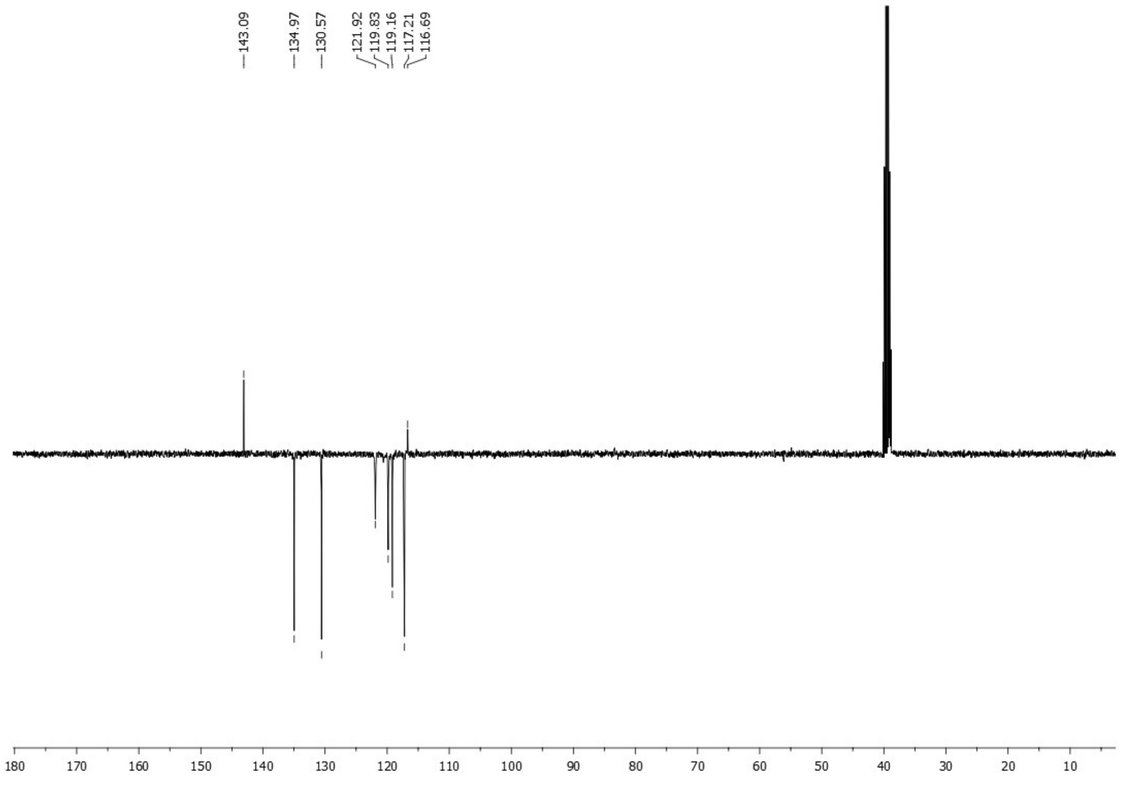

Figure S42. ^{13}C NMR spectrum (DMSO-d_6, 100 MHz) of ***2-hydroxy-N-(6-nitrobenzothiazol-2-yl)benzamide 27***.

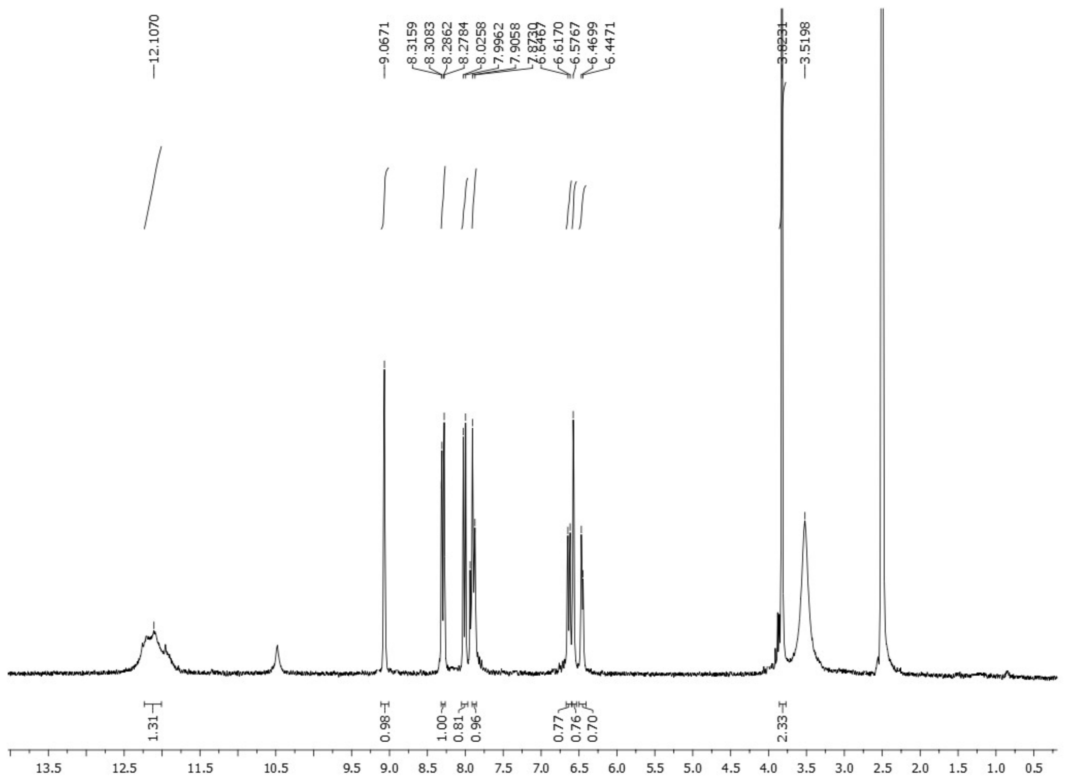

Figure S43. ^{1}H NMR spectrum (DMSO-d_6, 300 MHz) of ***2-hydroxy-4-methoxy-N-(6-nitrobenzothiazol-2-yl)benzamide 28***.

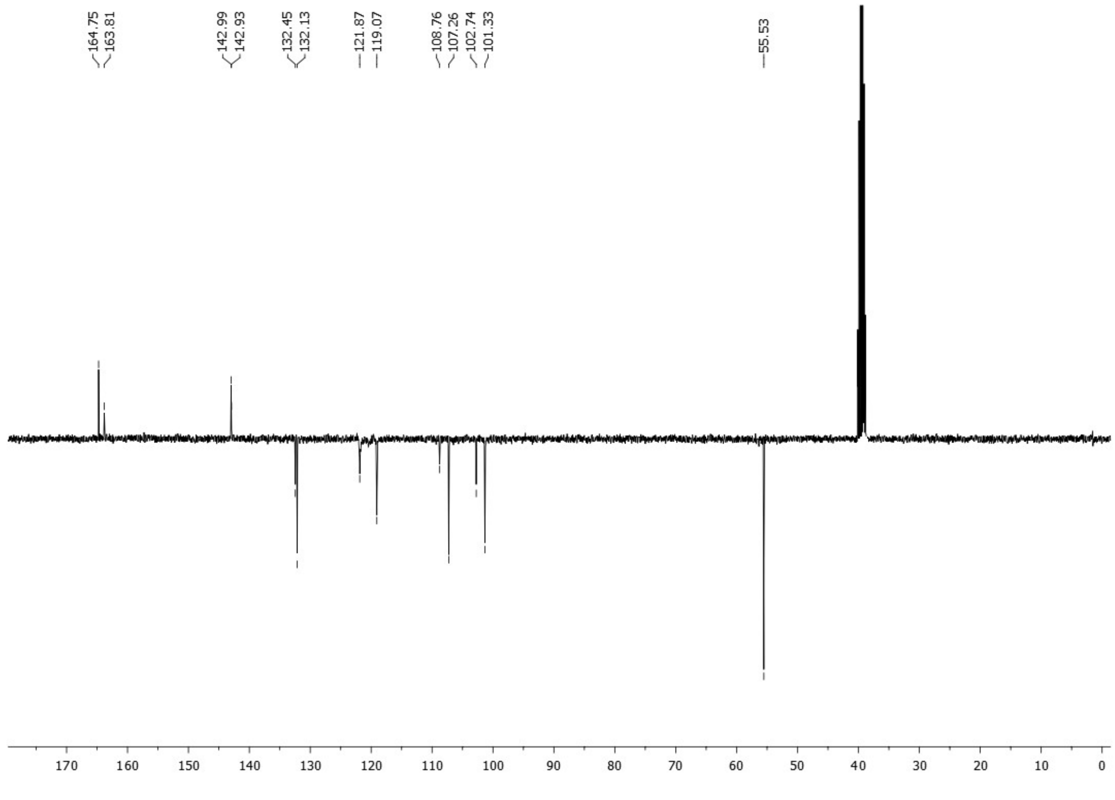

Figure S44. ^{13}C NMR spectrum (DMSO-d_6, 100 MHz) of ***2-hydroxy-4-methoxy-N-(6-nitrobenzothiazol-2-yl)benzamide 28***.

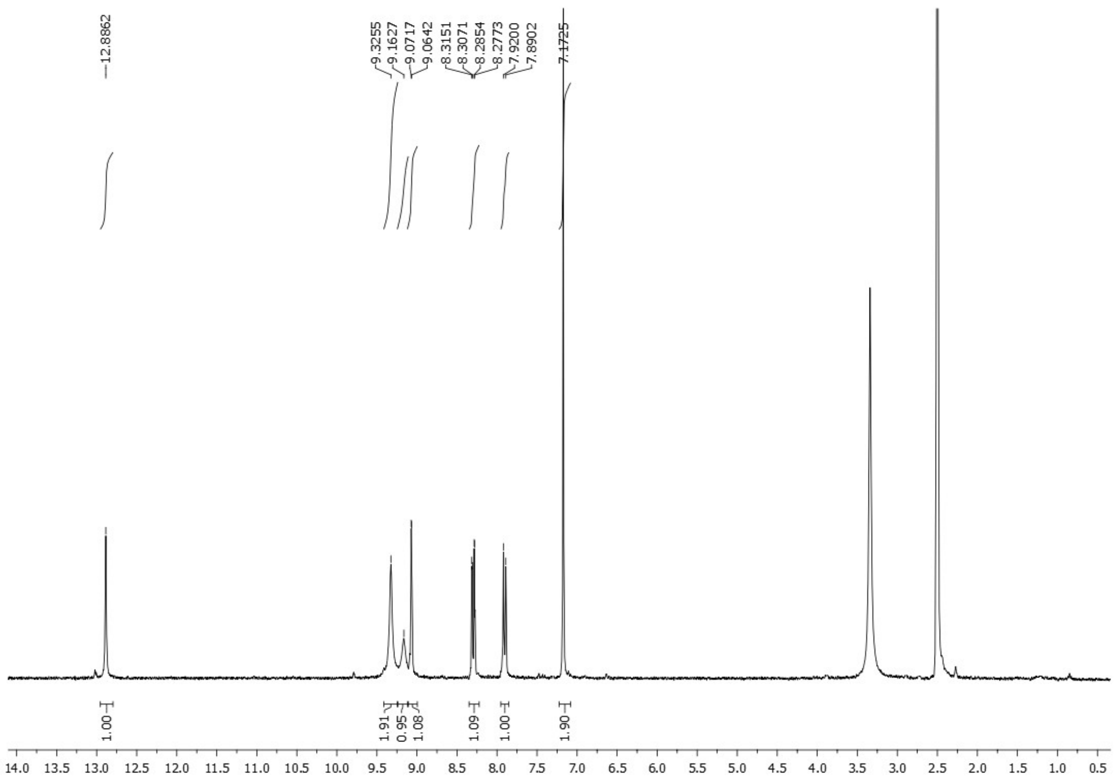

Figure S45. ^{1}H NMR spectrum (DMSO-d_6, 300 MHz) of ***3,4,5-trihydroxy-N-(6-nitrobenzothiazol-2-yl)benzamide 29***.

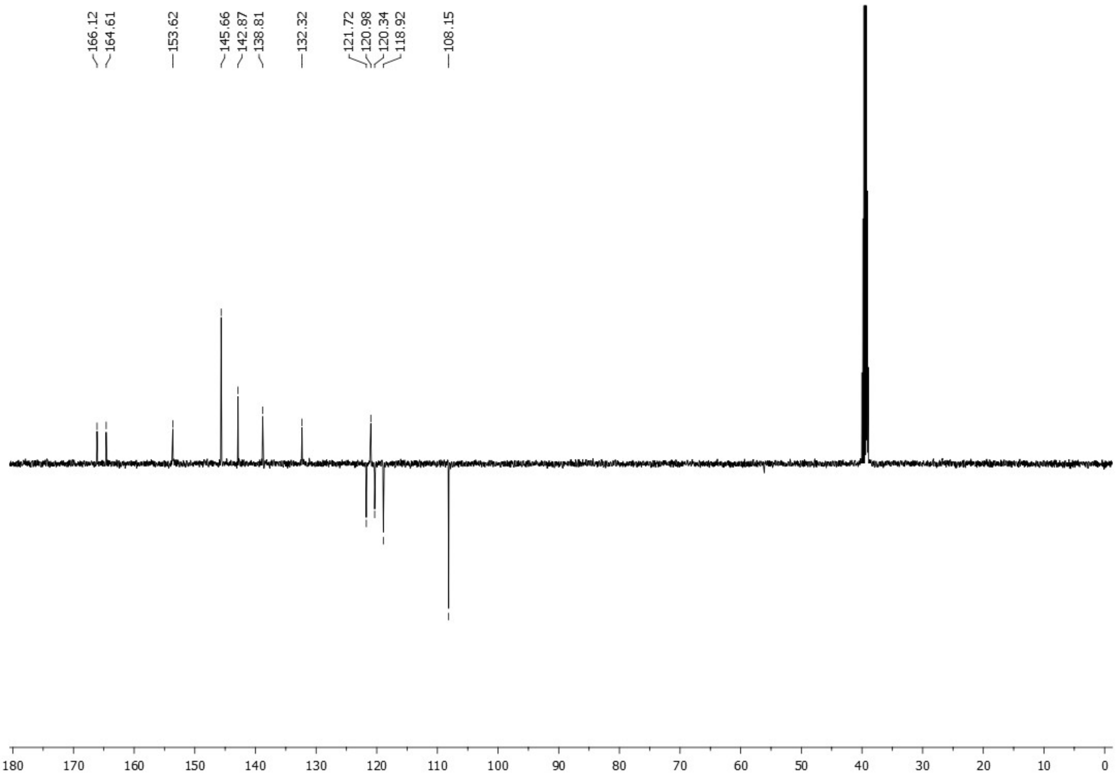

Figure S46. ^{13}C NMR spectrum (DMSO-d_6, 125 MHz) of ***3,4,5-trihydroxy-N-(6-nitrobenzothiazol-2-yl)benzamide 29***.

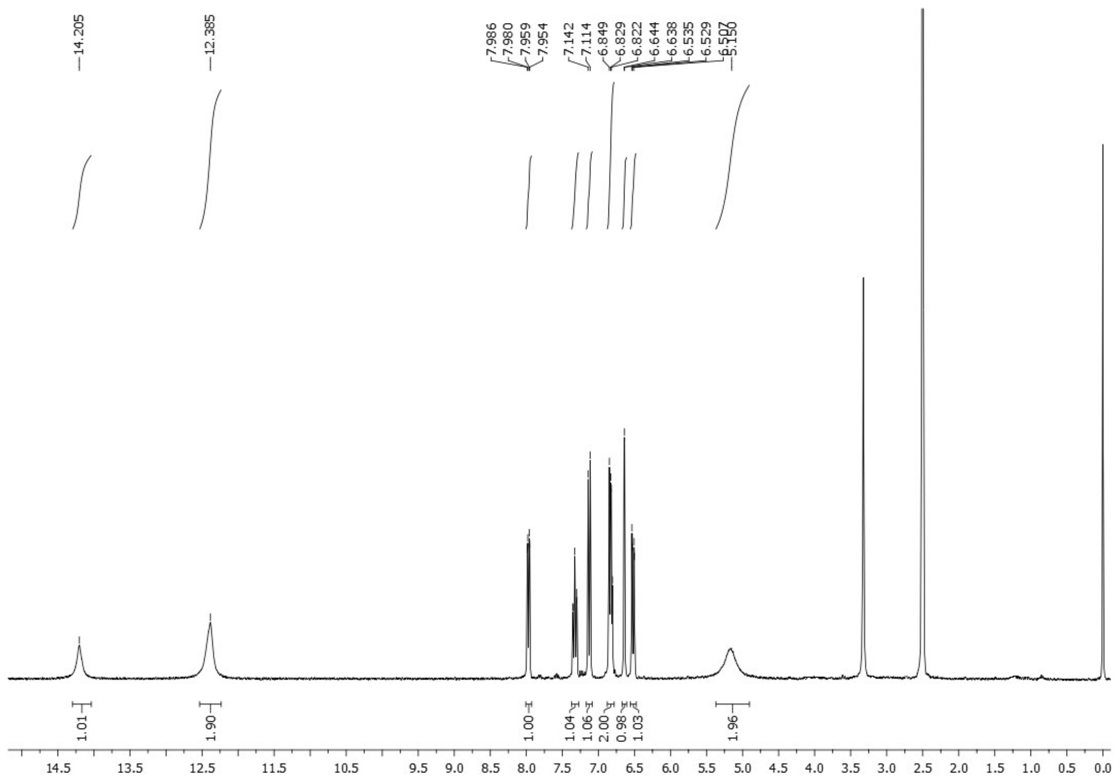

Figure S47. ^{1}H NMR spectrum (DMSO-d_6, 300 MHz) of ***N-[5(6)-aminobenzimidazol-2-yl]-2-hydroxybenzamide 30***.

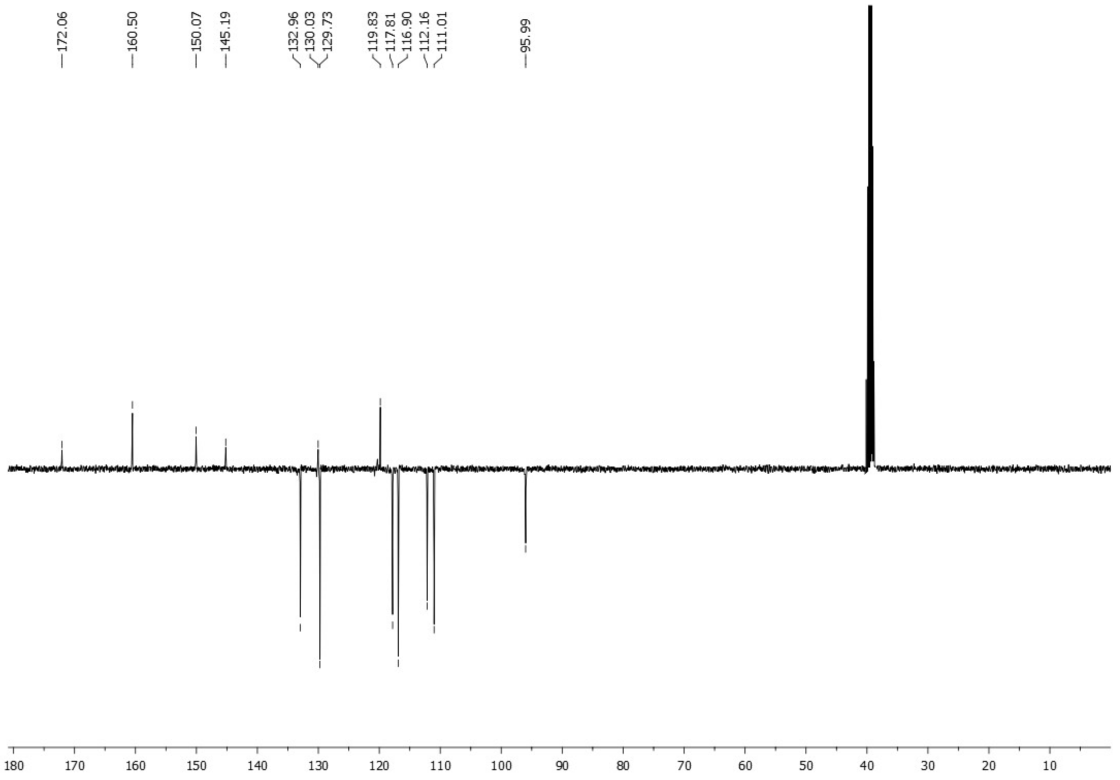

Figure S48. ^{13}C NMR spectrum (DMSO-d_6, 100 MHz) of ***N-[5(6)-aminobenzimidazol-2-yl]-2-hydroxybenzamide 30***.

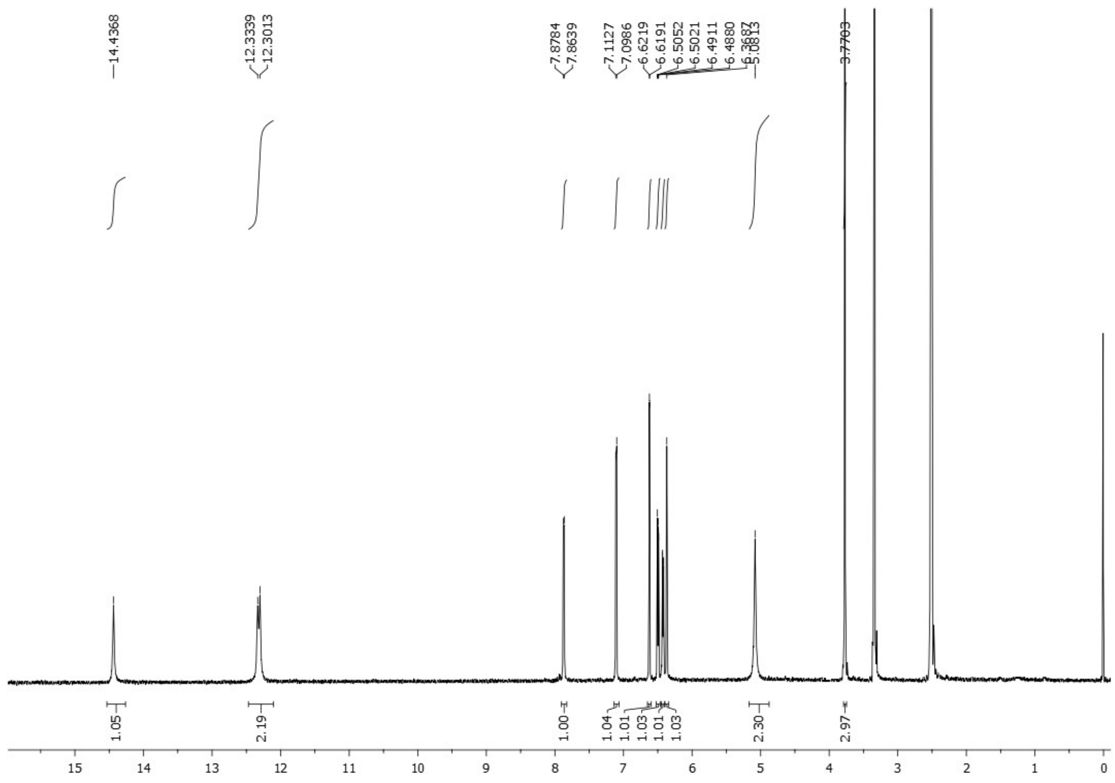

Figure S49. ^{1}H NMR spectrum (DMSO-d_6, 300 MHz) of ***N-[5(6)-aminobenzimidazol-2-yl]-2-hydroxy-4-methoxybenzamide 31***.

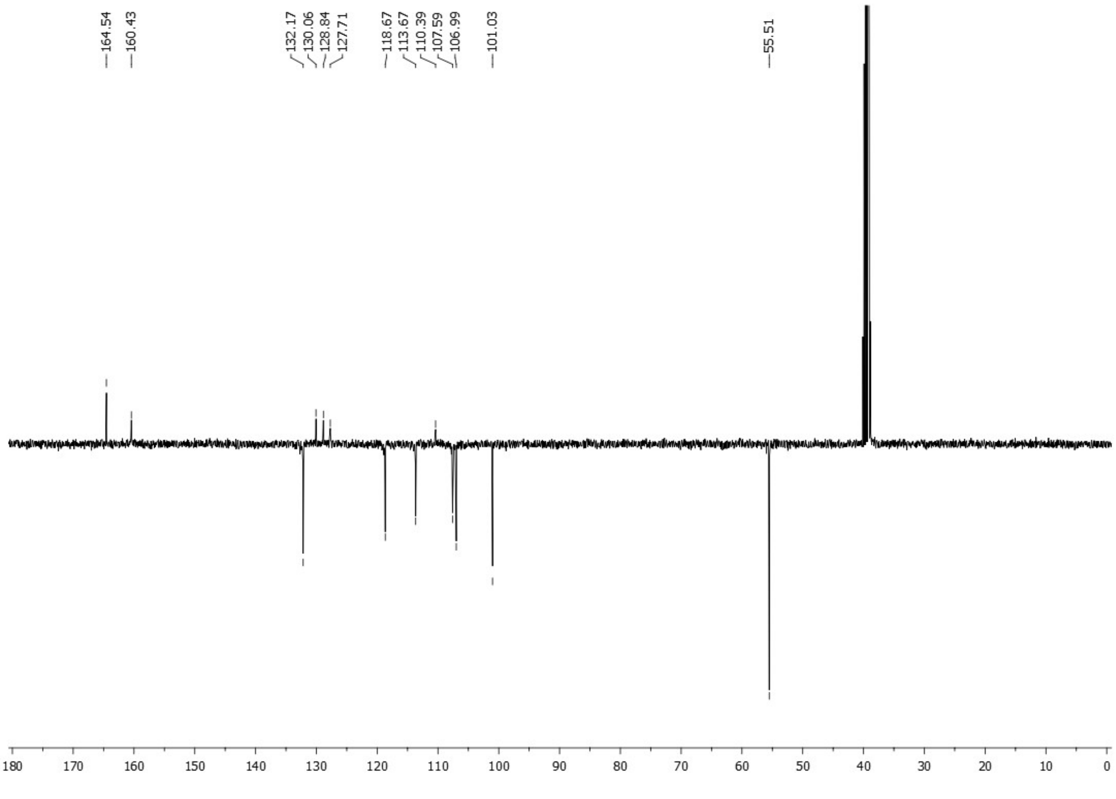

Figure S50. ^{13}C NMR spectrum (DMSO-d_6, 100 MHz) of ***N-[5(6)-aminobenzimidazol-2-yl]-2-hydroxy-4-methoxybenzamide 31***.

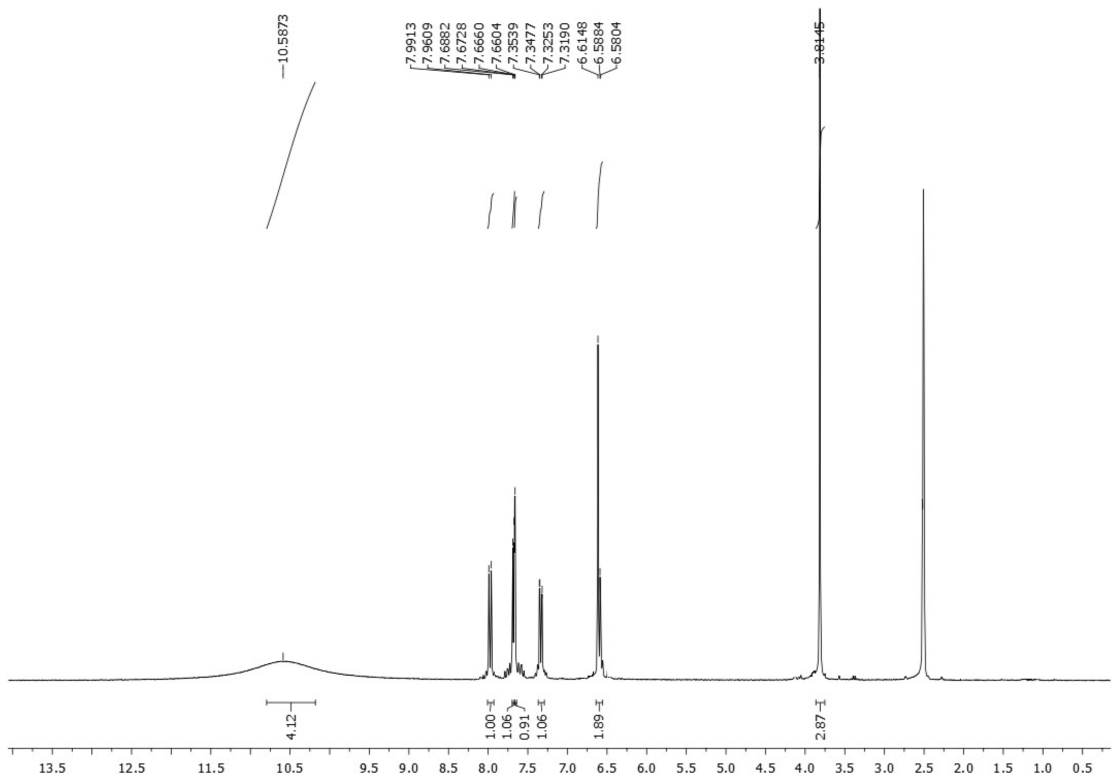

Figure S51. ^{1}H NMR spectrum (DMSO-d_6, 300 MHz) of ***N-[5(6)-aminobenzimidazol-2-yl]-2-hydroxy-4-methoxybenzamide 32***.

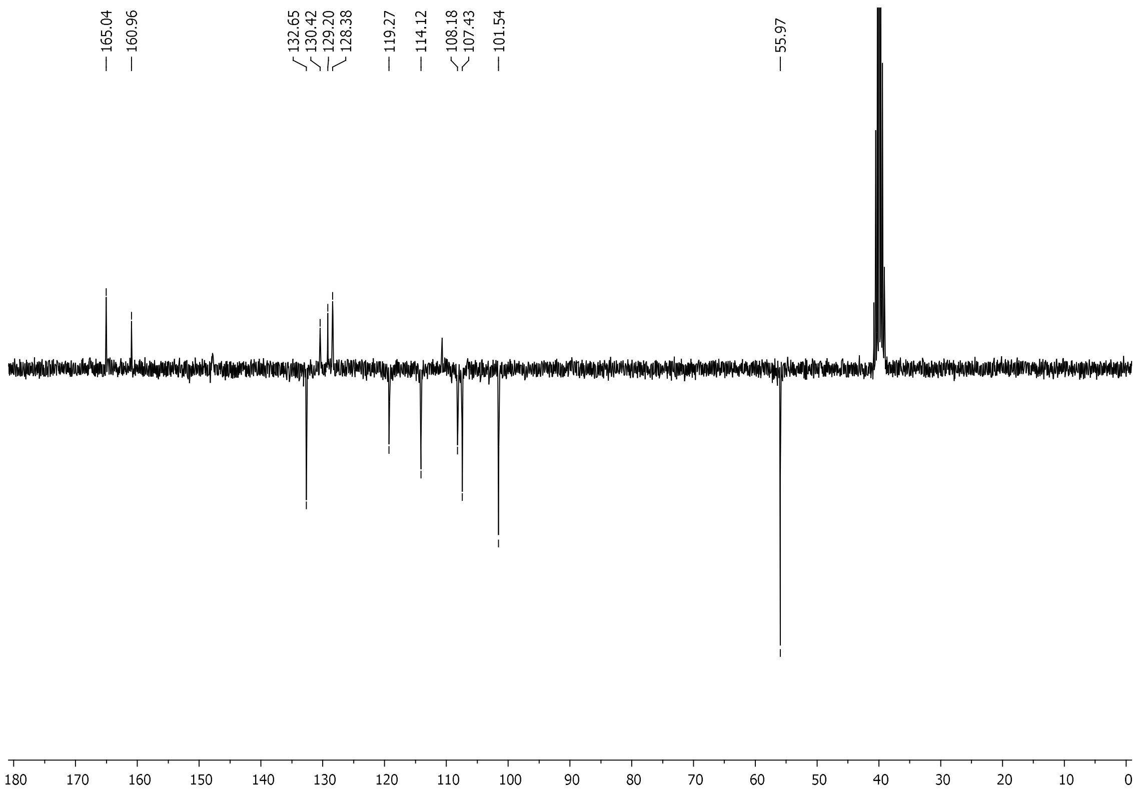

Figure S52. ^{13}C NMR spectrum (DMSO-d_6, 75 MHz) of ***N-[5(6)-aminobenzimidazol-2-yl]-2-hydroxy-4-methoxybenzamide 32***.

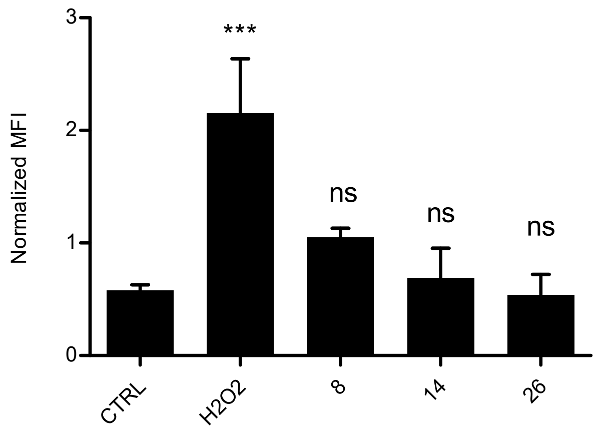

Figure S53. Impact of compounds on cellular ROS production was measured with fluorescent dye DCF-DA in HCT116 cell line, after the treatment with 10 μM compounds for 1h. H_2O_2 was used as a positive control. Data presented here are the results of 3 independent measurements, done in duplicates. One-way ANOVA with Tukey's post-hoc test was used for statistical analysis, ***- $p < 0.001$.

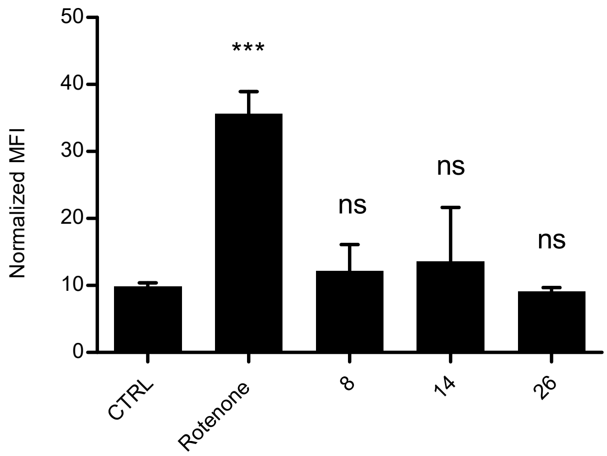

Figure S54. Impact of compounds on mitochondrial ROS production was measured with fluorescent dye MitoSOX in HCT116 cell line, after the treatment with 10 μM compounds for 1h. Rotenone, which interferes with electron transport chain in mitochondria and induces ROS formation was used as a positive control. Data presented here are the results of 3 independent measurements, done in duplicates. One-way ANOVA with Tukey's post-hoc test was used for statistical analysis, ***- $p < 0.001$.

Materials and methods

Cell culturing

Human colon carcinoma cell line HCT116 was grown in DMEM medium with the addition of 10% fetal bovine serum (FBS), 2 mM L-glutamine, 100 U/ml penicillin and 100 µg/ml streptomycin, and cultured as monolayers at 37°C in a humidified atmosphere with 5% CO2.

Cellular ROS measurement assay

For the cellular ROS measurement assay, 2.5×10^4 cells were seeded into 96-well microtiter plates 24h prior to experiment. Next day, cells were trypsinized and incubated in FBS-free DMEM medium with 20 µM DCFH-DA fluorescence dye for 45 minutes. After the incubation, compounds were added without washing and cells were treated with 2 mM H_2O_2 as a positive control and 10 µM compounds **8**, **14** and **26** for 1 hour. Cells were washed in PBS and DCFH-DA signal was measured by flow cytometry, in FL1 channel.

Mitochondrial ROS measurement assay

For the mitochonrial ROS measurement assay, 2.5×10^4 cells were seeded into 96-well microtiter plates 24h prior to experiment. Next day, cells were trypsinized and treated with 3 µM Rotenone as a positive control and 10 µM compounds **8**, **14** and **26** for 1 hour. After the treatment, cells were stained, without washing, with 5 µM MitoSOX fluorescent dye for 30 minutes. Cells were washed in PBS and MitoSOX signal was measured by flow citometry, in FL2 channel.
